# Impact of inflammatory and nutritional parameters on mortality in cardiovascular multimorbidity: a comprehensive prognostic analysis based on two datasets

**DOI:** 10.3389/fnut.2025.1702364

**Published:** 2025-11-21

**Authors:** Ziqi Chen, Aijing Zhu, Xu Zhu, Qiang Qu, Yang Ying, Sitong Chen, Haifeng Zhang, Iokfai Cheang, Xinli Li

**Affiliations:** 1 State Key Laboratory for Innovation and Transformation of Luobing Theory, Department of Cardiology, the First Affiliated Hospital with Nanjing Medical University, Jiangsu Province Hospital, Nanjing, China; 2 Department of Gastroenterology, Zhongda Hospital, School of Medicine, Southeast University, Nanjing, China; 3 Department of Cardiology, the Affiliated Suzhou Hospital of Nanjing Medical University, Suzhou Municipal Hospital, Gusu School, Suzhou, China

**Keywords:** cardiovascular multimorbidity (CMM), systemic inflammation response index (SIRI), nomogram prediction, risk stratification, machine learning (ML)

## Abstract

**Background:**

Cardiovascular multimorbidity (CMM), defined as the coexistence of multiple cardiometabolic diseases, has posed an escalating global health burden associated with premature mortality. Systemic inflammation has been increasingly recognized as a central mechanism linking cardiometabolic diseases, yet the prognostic implications of routine inflammatory and nutritional biomarkers in patients with CMM remained unclear.

**Methods:**

This cohort study analyzed 1,928 CMM patients from the National Health and Nutrition Examination Survey (NHANES) and 364 patients from a Chinese cohort (Gaoyou). Ten inflammatory and nutritional parameters were evaluated. Associations with all-cause and cardiovascular mortality were assessed using multivariable Cox regression and restricted cubic splines. Feature selection (SHAP, Boruta, and Lasso) was employed to identify optimal predictors, followed by construction and validation of nomogram and machine learning (ML) models.

**Results:**

The systemic inflammation response index (SIRI) emerged as the strongest independent predictor of mortality. Patients in the highest SIRI quartile exhibited significantly increased risks of all-cause mortality (HR = 2.34, 95% CI: 1.88–2.90) and cardiovascular mortality (HR = 2.09, 95% CI: 1.47–2.98), with consistent performance across various subgroups. Nomograms incorporating SIRI demonstrated excellent discrimination (AUCs > 0.7) and clinical utility. Among the ML models, XGBoost achieved the highest predictive efficiency at 60, 120, and 150 months.

**Conclusion:**

SIRI, reflecting the combined influence of inflammatory responses and nutritional status, provided an available and independent biomarker for mortality risk stratification in CMM patients. The validated nomograms and web-based prediction tool offered clinicians a practical approach for individualized prognosis and informed future strategies targeting systemic inflammation and nutrition in multimorbidity management.

## Introduction

Cardiovascular multimorbidity (CMM), defined as the coexistence of multiple cardiovascular and metabolic conditions such as stroke, diabetes mellitus (DM), and other cardiovascular diseases (CVDs), has emerged as a significant global public health challenge (1). The prevalence of CMM is increasing due to aging populations, sedentary lifestyles, and the growing burden of chronic diseases (2). This epidemic not only escalates healthcare costs but also severely impacts patient outcomes, leading to higher mortality rates and reduced quality of life (3). Understanding the underlying mechanisms driving CMM progression and mortality is essential for developing effective preventive and therapeutic strategies (4).

Inflammation and nutrition have been identified as central factors in the pathogenesis and progression of CMM. Chronic low-grade inflammation contributes to endothelial dysfunction, insulin resistance, and atherosclerosis, all of which are hallmark features of CMM (5–7). Furthermore, inflammatory and nutritional pathways are intricately linked to the development of complications such as heart failure, stroke, and renal dysfunction (8, 9). Emerging evidence suggests that systemic inflammation is a key driver of adverse outcomes in CMM, including all-cause and cardiovascular mortality (10). Therefore, identifying reliable inflammatory and nutritional biomarkers that can predict mortality risk in CMM patients is of critical importance.

In recent years, several parameters derived from complete blood counts have gained attention for their prognostic value in chronic diseases. These include the neutrophil-to-lymphocyte ratio (NLR), platelet-to-lymphocyte ratio (PLR), platelet-to-neutrophil ratio (PNR), systemic immune-inflammation index (SII), systemic inflammation response index (SIRI), neutrophil-to-high-density lipoprotein cholesterol ratio (NHR), monocyte-to-high-density lipoprotein cholesterol ratio (MHR), platelet-to-high-density lipoprotein cholesterol ratio (PHR), lymphocyte-to-high-density lipoprotein cholesterol ratio (LHR), and neutrophil-to-monocyte ratio (NMR). These parameters are cost-effective, easily accessible, and provide valuable insights into the inflammatory status of patients (11, 12). They have been widely used in research to predict outcomes in various conditions, including cancer, diabetes, and CVD. However, their collective role in predicting mortality in CMM remains underexplored.

This study aims to comprehensively evaluate the impact of 10 inflammatory and nutritional parameters (NLR, PLR, PNR, SII, SIRI, NHR, MHR, PHR, LHR, and NMR) on all-cause and cardiovascular mortality in patients with CMM. By leveraging a large public dataset, a self-constructed regional dataset, and advanced statistical methods, we seek to identify the most robust inflammatory and nutritional predictors of mortality. Additionally, we aim to develop nomograms validated by machine learning (ML) for clinical use. The rationale for this study lies in the urgent need for reliable tools to stratify mortality risk in CMM patients, enabling personalized interventions and improving outcomes. Our findings will contribute to the growing body of evidence on the role of inflammation in CMM and provide practical tools for clinicians to enhance patient care.

## Methods

### Study design and data sources

This study employed a retrospective cohort design, utilizing data from two sources: the National Health and Nutrition Examination Survey (NHANES), a large-scale, nationally representative dataset, and a cross-sectional community study conducted in Gaoyou County, Jiangsu Province, China. The study adhered to the principles of the Helsinki Declaration. These datasets were selected for their extensive health metrics, complete longitudinal follow-up, and inclusion of diverse populations, ensuring the generalizability of the findings.

For the NHANES dataset, ethical approval was obtained from the review boards, and informed consent was waived due to the use of de-identified, publicly available data. Data were accessed from https://wwwn.cdc.gov/nchs/nhanes/continuousnhanes.

The Gaoyou study protocol was approved by the independent ethics committee of the First Affiliated Hospital with Nanjing Medical University (13, 14). Participants were recruited using computerized random sampling from census lists provided by local authorities, stratified by gender and age. A total of 4,536 individuals (participation rate: 75.6%) signed the informed consent. Detailed fieldwork methods have been previously published (13, 14). The NHANES data and Gaoyou data was all collected before September 1, 2025.

### Study population

This study utilized NHANES data from 2003 to 2018, focusing on CMM participants. The analysis included 10 composite inflammatory and nutritional indicators, baseline information (e.g., physical examinations, blood tests, questionnaire surveys), and follow-up records, resulting in a sample size of 1,928 participants from eight survey cycles with a median follow-up of 70 months.

**Figure 1:**
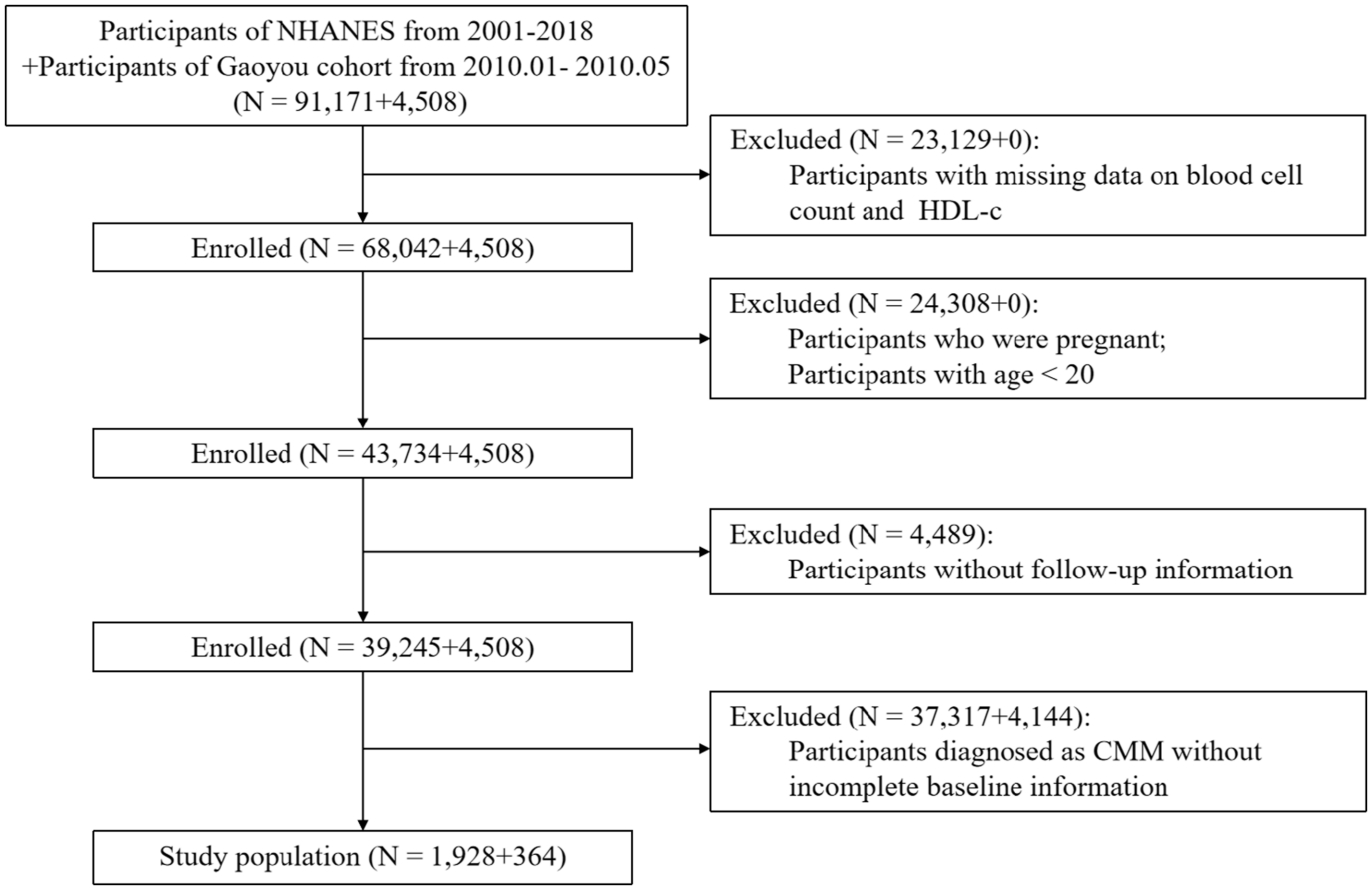
Study flowchart.

The Gaoyou dataset included analogous variables (excluding ethnicity and PIR) with reported medical history and was analyzed similarly, maintaining consistency in diagnostic criteria and follow-up procedures. Of the 4,508 participants from Gaoyou County, Jiangsu Province, China, 364 were included in the validation analysis, with a median follow-up of 52 months (Figure 1).

### Definition of CMM

CMM is defined as the coexistence of two or more cardiometabolic diseases, including diabetes mellitus (DM), stroke, and other cardiovascular diseases (e.g., coronary heart disease, angina, congestive heart failure, or any other heart problem). Diagnostic criteria for each condition were based on clinical guidelines, laboratory measurements, and self-reported medical histories (1).

DM was defined as fasting blood glucose (FBG) > 126 mg/dL, HbA1c ≥ 6.5%, medical diagnosis, or use of glucose-lowering medication (15). Chronic kidney disease (CKD) was diagnosed using an estimated glomerular filtration rate (eGFR) < 60 mL/min per 1.73 m^2^, derived from the 2021 race- and ethnicity-free Chronic Kidney Disease Epidemiology Collaboration creatinine equation (CKD-EPI) (16). Hypertension was characterized as systolic blood pressure (SBP) ≥ 140 mmHg, diastolic blood pressure (DBP) ≥ 90 mmHg, antihypertensive medication use, or clinical diagnosis (15). The presence of cardiovascular and cerebrovascular diseases was determined using standardized questionnaire-based surveys: “Has a doctor or other health professional ever told you that you had heart failure/coronary heart disease/angina/angina pectoris/heart attack?” and “Has a doctor or other health professional ever told you that you had stroke?”

### Measurement of inflammatory and nutritional indicators

We used listed formulas to calculate indicators in Supplementary Table S1. These parameters were calculated using complete blood count and laboratory results accordingly.

### Covariates

Demographic characteristics collected included age, gender, ethnicity, income, and educational level. Age was categorized using 60 years as the critical point. Ethnicity was specified as Non-Hispanic Black, Non-Hispanic White, Hispanic, or other races. A broader definition of Hispanics was applied, aggregating Mexican Americans and other Hispanic subgroups. Household income and poverty status were categorized using the poverty income ratio (PIR) as low (<1.3), middle (1.3–3.5), and high (≥3.5).

Drinking status was categorized into excessive, moderate, or light alcohol consumption. Excessive alcohol consumption was defined as three drinks per day for women and four drinks per day for men. Moderate consumption was defined as two drinks per day for women and three drinks per day for men. Remaining alcohol consumption was classified as light. Smoking status was divided into three categories: current smokers, former smokers, or never smokers. Never smokers were defined as having smoked fewer than 100 cigarettes in their lifetime, former smokers as having smoked more than 100 cigarettes but having quit at the time of the survey, and current smokers as having smoked more than 100 cigarettes and continuing to smoke occasionally or consistently (17).

### Outcome measures

Mortality outcomes were determined using the National Death Index (NDI). Follow-up time was calculated from the date of survey participation through 31 December, 2019. Vital status codes (0 = assumed alive, 1 = assumed deceased) were assigned to each participant. Cardiovascular deaths were identified based on underlying causes of death (UCOD): UCOD001 (Diseases of the heart) and UCOD005 (Cerebrovascular diseases).

### Statistical analysis

Statistical analysis began with baseline characterization. Continuous variables were presented as means (standard deviation, SD) or medians (interquartile range, IQR), depending on normality. Group comparisons were performed using Student’s *t*-test for normally distributed data or the Mann–Whitney *U* test for non-normally distributed data. Categorical variables were reported as counts (percentages) and compared using chi-square (*χ*^2^) tests. Missing data (6.72% for PIR) were addressed using multiple imputation via the “mice” package with a random forest model for optimal data imputation.

Prior to multivariable regression, variance inflation factor (VIF) analysis was conducted to verify collinearity, retaining variables with VIF < 10 to ensure model stability. Survival analysis involved stratifying each inflammatory and nutritional parameter into quartiles, constructing Kaplan–Meier (KM) curves to visualize cumulative survival differences across quartiles, and assessing significance using log-rank tests for all-cause and cardiovascular mortality. Cox proportional hazards models were used to estimate hazard ratios (HR) and 95% confidence intervals (CI), with three adjustment strategies: an unadjusted model, a partly adjusted model (incorporating gender, age, ethnicity, education, and PIR), and a fully adjusted model [further including SBP, DBP, eGFR, body mass index (BMI), uric acid (UA), glycated hemoglobin (HbA1c), total cholesterol (TC), high-density lipoprotein cholesterol (HDL-C), smoking, drinking, hypertension, DM, liver disease, cancer, and CVD].

Nonlinear associations between parameters and mortality were explored using restricted cubic spline (RCS) regression within the fully adjusted model framework, setting knots at the 5th, 50th, and 95th percentiles of variable distributions to visualize dose–response trends. Forest plots were generated based on fully adjusted Cox results to illustrate HRs and CIs for each parameter’s median (referencing the lower quartile), enabling intuitive comparison of effect sizes.

Single-variable logistic regression models were constructed for each of the 10 inflammatory and nutritional variables, with predictive power compared via receiver operating characteristic (ROC) curves, calculating area under the curve (AUC), optimal cutoff values, sensitivity, specificity, and other metrics. Feature selection involved building a model with all baseline variables and 10 markers, followed by SHAP value analysis, Boruta algorithm, and Lasso regression to identify the optimal factor across all three methods.

Subsequently, a model integrating all baseline variables and the chosen factor underwent variable selection using Boruta, Lasso regression, and stepwise regression, with variables appearing in ≥2 methods highlighted in red in tables. Predictive models for all-cause and cardiovascular mortality were developed using Cox analysis, calculating AUCs at 60-month, 120-month, and 150-month time points. Nomograms were constructed for these intervals, and corresponding C-indices were calculated. For internal validation, the dataset was divided into training and validation sets in a 7:3 ratio, with validation performed using calibration curves and decision curve analysis (DCA) at the same time points in both sets.

In addition to Cox analysis, prognostic models were developed using eight machine learning (ML) approaches, including logistic classification, XGBoost, LightGBM, random forest, AdaBoost, decision tree (DT), neural network, and support vector machine (SVM), with 70% of the samples allocated to the training set and the remaining 30% reserved for the validation set. We assessed predictive efficacy through AUCs, cutoff values, accuracy, sensitivity, specificity, positive and negative predictive values, F1 scores, and Kappa coefficients at 60, 120, and 150 months. The performance of all pattern parameters was systematically evaluated through ROC analysis of the prognostic models, with stability demonstrated by calibration curves and DCA. Finally, an online prediction website for all-cause and cardiovascular mortality was developed based on the validated effective models.

Especially for Gaoyou cohort, all laboratory and anthropometric variables were standardized prior to analyses. Continuous variables were converted to uniform measurement units (e.g., 10^9^/L for cell counts, mg/dL for HDL-C), and distributions were z-score normalized within each dataset to eliminate scale differences. Meanwhile, the same diagnostic and inclusion criteria for hypertension/diabetes/chronic kidney disease/stroke/cardiometabolic diseases were applied across cohorts to minimize classification bias. The variable “Ethnicity” and “PIR” were not recorded.

All analyses were performed using R Project and Python (version 3.8) for Statistical Computing (version 4.4.5). A two-sided *p*-value < 0.05 was considered statistically significant.

## Results

### Baseline characteristics

Baseline demographic and clinical characteristics of the cohort are summarized in Table 1. The study included 1,928 individuals, of whom 1,095 (56.8%) were alive and 833 (43.2%) had died. Significant differences were observed between the alive and deceased groups across multiple variables, including target parameters and a range of comorbidities. Variance inflation factor (VIF) analysis for 10 parameters and covariates confirmed the absence of severe collinearity, with all VIF values <10 (Supplementary Tables S1, S2), ensuring the stability of subsequent regression analyses.

**Table 1:** Baseline characteristics.

Variables	Total (*n* = 1,928)	Alive (*n* = 1,095)	Death (*n* = 833)	*p*
Age	67.76 ± 10.98	64.61 ± 11.20	71.91 ± 9.16	**<0.001**
Gender				0.137
Male	1,104 (57.26)	611 (55.80)	493 (59.18)	
Female	824 (42.74)	484 (44.20)	340 (40.82)	
BMI	31.84 ± 7.28	32.68 ± 7.26	30.74 ± 7.16	**<0.001**
WAIST	101.36 ± 32.15	105.20 ± 27.77	96.31 ± 36.54	**<0.001**
SBP	134.94 ± 22.31	133.19 ± 20.63	137.24 ± 24.16	**<0.001**
DBP	66.57 ± 15.96	68.41 ± 14.82	64.15 ± 17.04	**<0.001**
UA	6.14 ± 1.74	5.88 ± 1.55	6.48 ± 1.90	**<0.001**
HbA1c	6.95 ± 1.59	7.00 ± 1.58	6.88 ± 1.60	0.086
HDL-C	47.60 ± 14.89	47.46 ± 14.03	47.79 ± 15.95	0.624
TC	178.00 ± 45.98	177.75 ± 45.42	178.32 ± 46.72	0.790
Ethnicity				**<0.001**
Hispanic	372 (19.29)	258 (23.56)	114 (13.69)	
Non-hispanic White	967 (50.16)	461 (42.10)	506 (60.74)	
Non-hispanic Black	459 (23.81)	280 (25.57)	179 (21.49)	
Other race	130 (6.74)	96 (8.77)	34 (4.08)	
Education				**<0.001**
Below high school	735 (38.12)	375 (34.25)	360 (43.22)	
High school	468 (24.27)	271 (24.75)	197 (23.65)	
Above high school	725 (37.60)	449 (41.00)	276 (33.13)	
PIR				**0.006**
<1.3	746 (38.69)	439 (40.09)	307 (36.85)	
1.3–3.5	796 (41.29)	419 (38.26)	377 (45.26)	
≥3.5	386 (20.02)	237 (21.64)	149 (17.89)	
Hypertension				0.699
No	294 (15.25)	170 (15.53)	124 (14.89)	
Yes	1,634 (84.75)	925 (84.47)	709 (85.11)	
eGFR				**0.027**
< 60	800 (41.49)	478 (43.65)	322 (38.66)	
≥ 60	1,128 (58.51)	617 (56.35)	511 (61.34)	
Diabetes				0.141
No	304 (15.77)	161 (14.70)	143 (17.17)	
Yes	1,624 (84.23)	934 (85.30)	690 (82.83)	
Smoking				**0.007**
Never	751 (38.95)	444 (40.55)	307 (36.85)	
Former	826 (42.84)	436 (39.82)	390 (46.82)	
Current	351 (18.21)	215 (19.63)	136 (16.33)	
Alcohol Users				**<0.001**
Light	1,660 (86.10)	904 (82.56)	756 (90.76)	
Moderate	120 (6.22)	87 (7.95)	33 (3.96)	
Excessive	148 (7.68)	104 (9.50)	44 (5.28)	
Cardiovascular disease				**0.005**
No	291 (15.09)	187 (17.08)	104 (12.48)	
Yes	1,637 (84.91)	908 (82.92)	729 (87.52)	
Stroke				0.272
No	1,099 (57.00)	636 (58.08)	463 (55.58)	
Yes	829 (43.00)	459 (41.92)	370 (44.42)	
Liver disease				0.728
No	1,830 (94.92)	1,041 (95.07)	789 (94.72)	
Yes	98 (5.08)	54 (4.93)	44 (5.28)	
Cancer				**<0.001**
No	1,526 (79.15)	902 (82.37)	624 (74.91)	
Yes	402 (20.85)	193 (17.63)	209 (25.09)	
NLR	2.65 ± 1.61	2.42 ± 1.39	2.97 ± 1.82	**<0.001**
PLR	128.99 ± 61.18	122.59 ± 53.75	137.39 ± 68.90	**<0.001**
PNR	55.61 ± 26.88	57.83 ± 28.71	52.70 ± 23.97	**<0.001**
SII	608.39 ± 477.68	559.94 ± 368.07	672.07 ± 585.83	**<0.001**
SIRI	1.62 ± 1.16	1.45 ± 1.02	1.85 ± 1.29	**<0.001**
NHR	0.11 ± 0.06	0.11 ± 0.06	0.11 ± 0.06	**0.015**
MHR	0.01 ± 0.01	0.01 ± 0.01	0.01 ± 0.01	**0.007**
PHR	5.27 ± 2.45	5.29 ± 2.24	5.24 ± 2.69	0.636
LHR	0.05 ± 0.04	0.05 ± 0.02	0.05 ± 0.05	0.153
NMR	8.25 ± 4.15	8.17 ± 3.94	8.37 ± 4.41	0.287

BMI, body mass index; SBP, systolic blood pressure; DBP, diastolic blood pressure; UA, uric acid; HbA1c, hemoglobin A1c; HDL-C, high-density lipoprotein cholesterol; TC, total cholesterol; PIR, poverty income ratio; eGFR, estimated glomerular filtration rate; NLR, neutrophil-to-lymphocyte ratio; PLR, platelet-to-lymphocyte ratio; PNR, platelet-to-neutrophil ratio; SII, systemic immune-inflammation index; SIRI, systemic inflammation response index; NHR, neutrophil-to-high-density lipoprotein ratio; MHR, monocyte-to-high-density lipoprotein ratio; PHR, platelet-to-high-density lipoprotein ratio; LHR, lymphocyte-to-high-density lipoprotein ratio; NMR, neutrophil-to-monocyte ratio. The significant differences in the table (defined as *p* < 0.05) are highlighted in bold.

### Quartile stratification and cox proportional hazards models

Kaplan–Meier survival curves stratified by quartiles of each parameter revealed significant differences in cumulative survival for both all-cause and cardiovascular mortality (log-rank *p* < 0.001 for NLR, SIRI, and other parameters; Supplementary Figures S1A–J, S2A–J). In unadjusted and partly-adjusted models, the highest quartiles of NLR and SIRI demonstrated statistically significant associations with all-cause and cardiovascular mortality, highlighting their robust predictive value (Supplementary Tables S3, S4). After comprehensive adjustment for demographic characteristics, comorbid conditions, and biochemical biomarkers (Table 2), the highest quartile of SIRI was associated with the most pronounced risk of all-cause mortality (HR = 2.34, 95% CI: 1.88–2.90, *p* < 0.001) and cardiovascular mortality (HR = 2.09, 95% CI: 1.47–2.98, *p* < 0.001). A consistent dose–response relationship was observed for both mortality endpoints, with *p* for trend < 0.001, further substantiating the predictive strength of SIRI.

**Table 2:** Cox proportional hazards analysis of inflammatory and nutritional parameters for all-cause and cardiovascular mortality in fully-adjusted model.

Subgroup	Quartile				*p* for trend
	Q1	Q2	Q3	Q4	
All-cause mortality					
NLR	Reference	1.08 (0.87 ~ 1.34)	1.25 (1.01 ~ 1.56)	2.10 (1.70 ~ 2.60)	<0.001
PLR	Reference	1.05 (0.85 ~ 1.29)	1.13 (0.92 ~ 1.38)	1.23 (1.01 ~ 1.49)	0.026
PNR	Reference	0.67 (0.55 ~ 0.81)	0.63 (0.52 ~ 0.76)	0.52 (0.42 ~ 0.64)	<0.001
SII	Reference	1.07 (0.87 ~ 1.32)	1.15 (0.94 ~ 1.42)	1.64 (1.34 ~ 2.00)	<0.001
SIRI	Reference	1.16 (0.93 ~ 1.46)	1.47 (1.19 ~ 1.83)	2.34 (1.88 ~ 2.90)	<0.001
NHR	Reference	1.17 (0.94 ~ 1.46)	1.45 (1.15 ~ 1.84)	1.85 (1.43 ~ 2.40)	<0.001
MHR	Reference	1.12 (0.90 ~ 1.39)	1.50 (1.19 ~ 1.88)	1.38 (1.07 ~ 1.78)	<0.001
PHR	Reference	0.85 (0.70 ~ 1.04)	0.70 (0.56 ~ 0.88)	0.67 (0.52 ~ 0.87)	<0.001
LHR	Reference	0.74 (0.61 ~ 0.89)	0.59 (0.48 ~ 0.73)	0.55 (0.43 ~ 0.70)	<0.001
NMR	Reference	1.11 (0.91 ~ 1.35)	1.01 (0.83 ~ 1.23)	1.22 (1.00 ~ 1.48)	0.129
Cardiovascular mortality					
NLR	Reference	1.15 (0.82 ~ 1.61)	1.10 (0.78 ~ 1.56)	1.87 (1.34 ~ 2.62)	<0.001
PLR	Reference	0.99 (0.71 ~ 1.37)	1.17 (0.85 ~ 1.61)	1.37 (1.01 ~ 1.85)	0.020
PNR	Reference	0.79 (0.58 ~ 1.08)	0.74 (0.55 ~ 1.01)	0.75 (0.54 ~ 1.04)	0.070
SII	Reference	1.04 (0.76 ~ 1.43)	1.02 (0.74 ~ 1.41)	1.40 (1.02 ~ 1.91)	0.045
SIRI	Reference	1.47 (1.04 ~ 2.07)	1.45 (1.03 ~ 2.06)	2.09 (1.47 ~ 2.98)	<0.001
NHR	Reference	1.00 (0.72 ~ 1.39)	1.18 (0.83 ~ 1.68)	1.03 (0.68 ~ 1.56)	0.662
MHR	Reference	1.17 (0.84 ~ 1.62)	1.21 (0.84 ~ 1.73)	1.10 (0.74 ~ 1.64)	0.691
PHR	Reference	0.85 (0.62 ~ 1.16)	0.60 (0.42 ~ 0.87)	0.63 (0.42 ~ 0.94)	0.008
LHR	Reference	0.74 (0.55 ~ 0.99)	0.57 (0.41 ~ 0.80)	0.48 (0.33 ~ 0.70)	<0.001
NMR	Reference	1.12 (0.83 ~ 1.50)	0.96 (0.71 ~ 1.31)	0.95 (0.69 ~ 1.31)	0.573

Adjusted for gender, age, ethnicity, education, and poverty income ratio, systolic blood pressure, diastolic blood pressure, eGFR, BMI, uric acid, glycated hemoglobin (HbA1c), total cholesterol, HDL-C, smoking, drinking, hypertension, diabetes, liver disease, cancer, and CVD.

NLR, neutrophil-to-lymphocyte ratio; PLR, platelet-to-lymphocyte ratio; PNR, platelet-to-neutrophil ratio; SII, systemic immune-inflammation index; SIRI, systemic inflammation response index; NHR, neutrophil-to-high-density lipoprotein ratio; MHR, monocyte-to-high-density lipoprotein ratio; PHR, platelet-to-high-density lipoprotein ratio; LHR, lymphocyte-to-high-density lipoprotein ratio; NMR, neutrophil-to-monocyte ratio.

### Restricted cubic splines (RCS)

**Figure 2:**
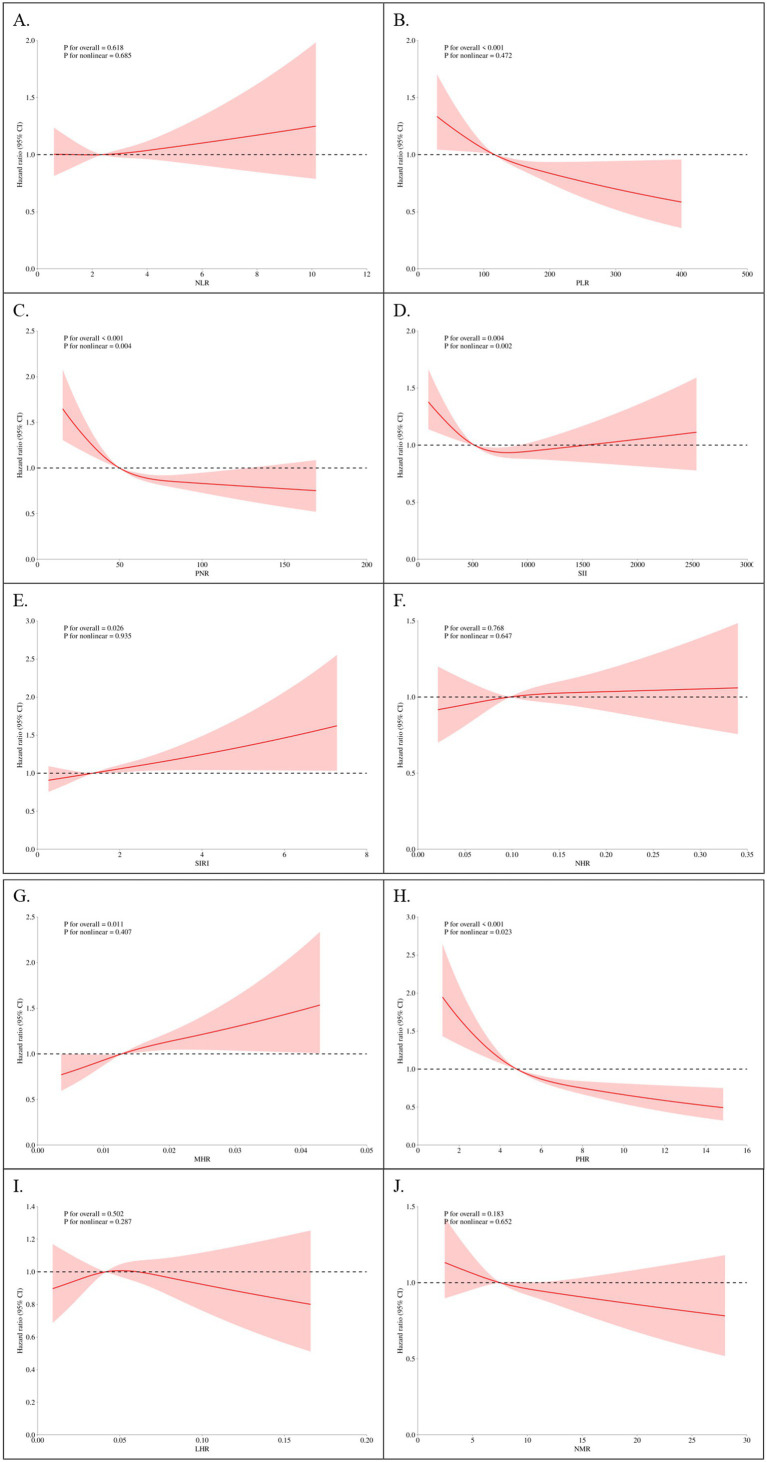
Restricted cubic spline plots of parameters and all-cause mortality. **(A)** NLR, Neutrophil-to-Lymphocyte Ratio; **(B)** PLR, Platelet-to-Lymphocyte Ratio; **(C)** PNR, Platelet-to-Neutrophil Ratio; **(D)** SII, Systemic Immune-Inflammation Index; **(E)** SIRI, systemic inflammation response index; **(F)** NHR, neutrophil-to-high-density lipoprotein ratio; **(G)** MHR, monocyte-to-high-density lipoprotein ratio; **(H)** PHR, platelet-to-high-density lipoprotein ratio; **(I)** LHR, lymphocyte-to-high-density lipoprotein ratio; **(J)** NMR, neutrophil-to-monocyte ratio. Knots set at 5th, 50th, and 95th percentiles of parameter distributions. Solid line = adjusted HR; shaded area = 95% CI. *X*-axis = standardized parameter values; *Y*-axis = HR relative to the median value of the variable (HR = 1 at the median).

**Figure 3:**
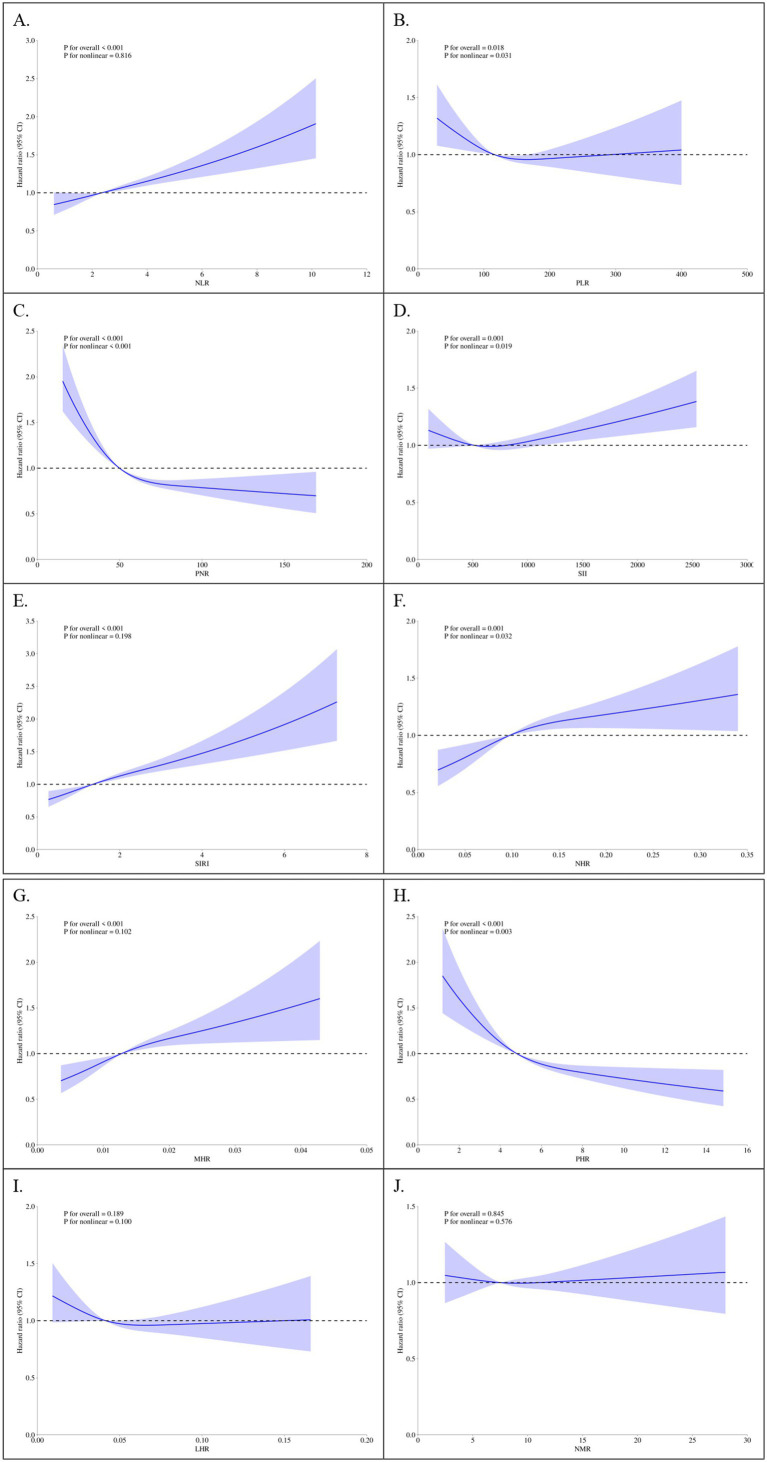
Restricted Cubic Spline Plots of Parameters and Cardiovascular Mortality. **(A)** NLR, neutrophil-to-lymphocyte ratio; **(B)** PLR, platelet-to-lymphocyte ratio; **(C)** PNR, platelet-to-neutrophil ratio; **(D)** SII, systemic immune-inflammation index; **(E)** SIRI, systemic inflammation response index; **(F)** NHR, neutrophil-to-high-density lipoprotein ratio; **(G)** MHR, monocyte-to-high-density lipoprotein ratio; **(H)** PHR, platelet-to-high-density lipoprotein ratio; **(I)** LHR, lymphocyte-to-high-density lipoprotein ratio; **(J)** NMR, neutrophil-to-monocyte ratio. Knots set at 5th, 50th, and 95th percentiles of parameter distributions. Solid line = adjusted HR; shaded area = 95% CI. *X*-axis = standardized parameter values; *Y*-axis = HR relative to the median value of the variable (HR = 1 at the median).

RCS regression was employed to explore nonlinear relationships between parameters and mortality outcomes. Under the fully-adjusted model, SIRI exhibited a clear dose–response relationship with all-cause mortality, with risk increasing significantly at higher quartiles (*p* for nonlinearity < 0.001; Figure 2E). Similar results were observed for cardiovascular mortality (Figure 3E). NLR and PLR demonstrated nonlinear associations, with risk escalating sharply beyond specific thresholds. These findings underscore the importance of considering nonlinear trends when evaluating the prognostic value of markers in CMM patients.

### Subgroup analysis

**Figure 4:**
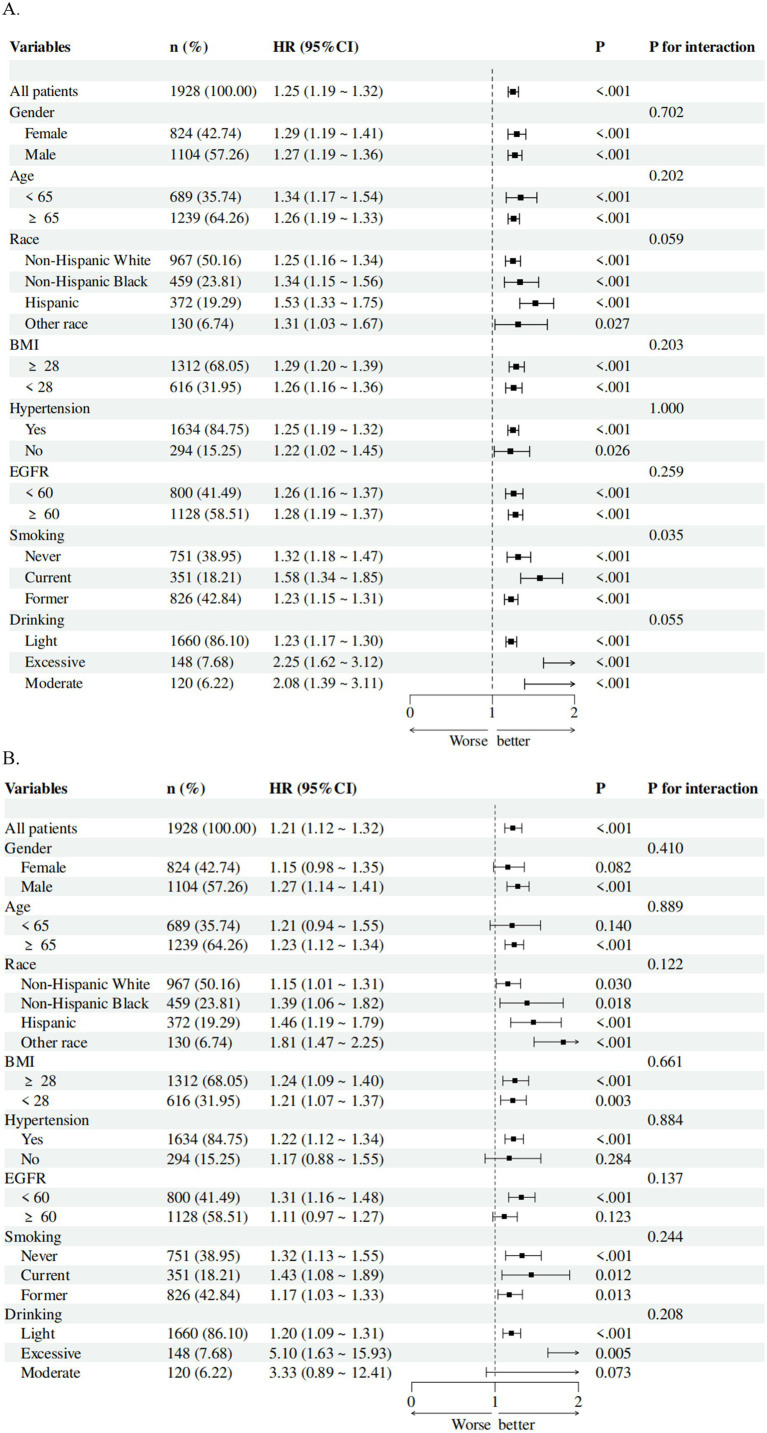
Subgroup analysis of the associations of SIRI with **(A)** all-cause mortality and **(B)** cardiovascular mortality in patients with CMM. The model was adjusted for gender, age, ethnicity, education, and poverty income ratio, systolic blood pressure, diastolic blood pressure, eGFR, BMI, uric acid, glycated hemoglobin, total cholesterol, HDL-C, smoking, drinking, hypertension, diabetes, liver disease, cancer, and CVD.

Subgroup analyses were conducted to assess the consistency of associations between parameters and mortality across different demographic and clinical subgroups. Forest plots based on fully adjusted Cox models revealed that SIRI remained a stable predictor of all-cause mortality (Figure 4A) and cardiovascular mortality (Figure 4B) across all subgroups, including age, gender, BMI, and comorbidity status. For instance, in patients aged ≥65 years, higher SIRI levels were associated with a 2.5-fold increased risk of all-cause mortality (HR = 1.26, 95% CI: 1.19–1.33, *p* < 0.001). Similarly, among patients with baseline kidney failure, SIRI demonstrated comparable prognostic utility (HR = 1.26, 95% CI: 1.13–1.37, *p* < 0.001).

### Single-variable predictive performance

Single-variable logistic regression models were evaluated using ROC curves (Supplementary Figure S3). SIRI and NLR exhibited remarkable independent predictive effects for all-cause mortality (AUC = 0.605). Conversely, LHR showed an advantage in forecasting cardiovascular mortality events (AUC = 0.558), followed by SIRI (AUC = 0.550) and NLR (AUC = 0.555). Optimal cutoff values varied widely (e.g., SIRI threshold for predicting all-cause mortality: 1.435; cardiovascular mortality: 0.917), with sensitivity/specificity trade-offs reflecting the clinical utility of each marker.

### Feature selection and model optimization

To identify crucial factors, SHAP value analysis, the Boruta algorithm, and Lasso regression were applied to a model incorporating all baseline variables and 10 markers. SIRI emerged as the most consistent predictor, demonstrating significance across all three methods (Supplementary Figures S4, S5). Subsequent variable selection for models integrating SIRI and baseline covariates (via Boruta, Lasso, and stepwise regression) identified key predictors for all-cause mortality (age, BMI, ethnicity, DBP, education, drinking, SIRI, uric acid, eGFR) and cardiovascular mortality (age, gender, BMI, ethnicity, CVD, drinking, SIRI, uric acid, eGFR), with variables substantiated in ≥2 methods highlighted (Supplementary Figures S6, S7 and Table S5).

### Nomogram development and dynamic model website

**Figure 5:**
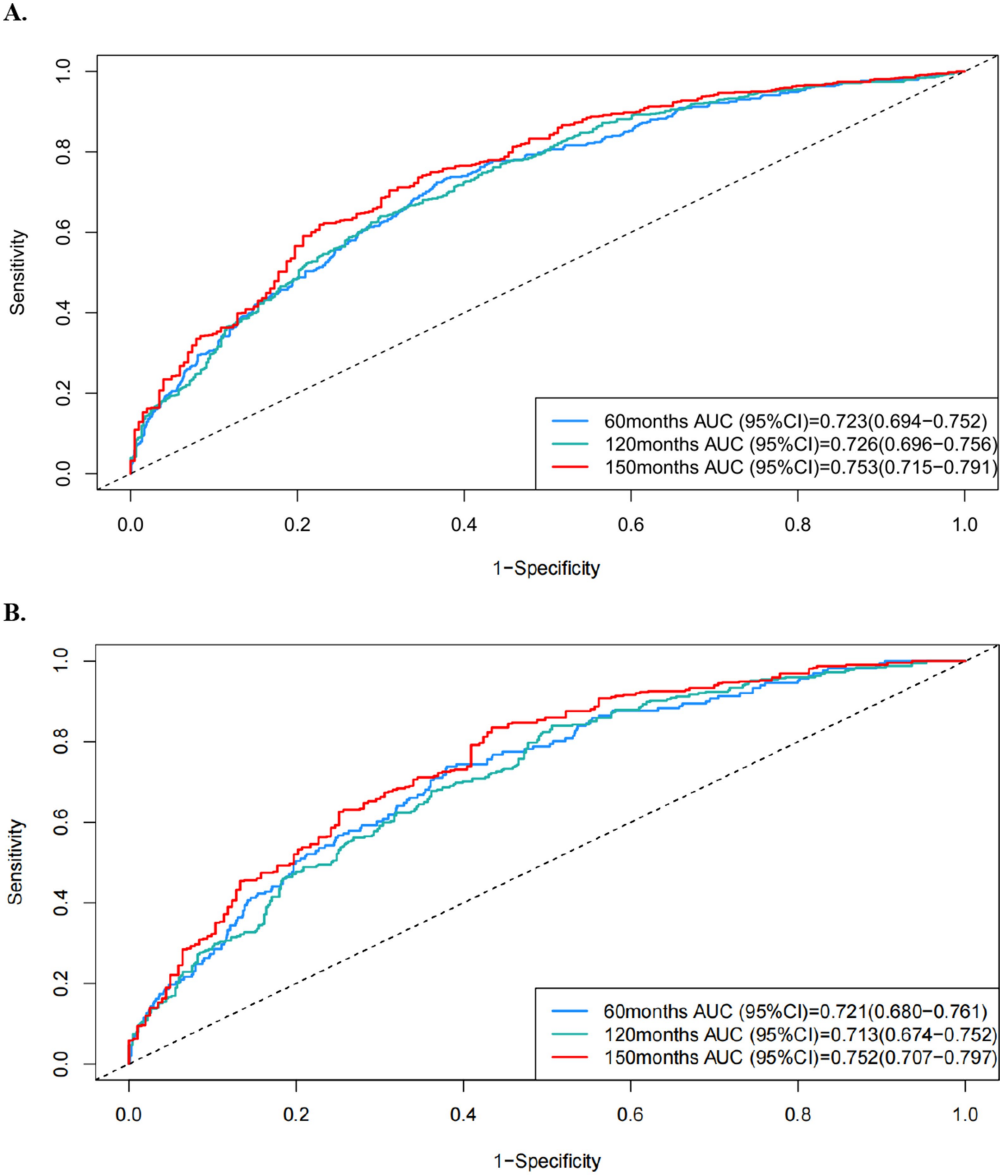
ROC curves of the formed prognostic model in predicting **(A)** all-cause and **(B)** cardiovascular mortality at 60-month, 120-month, and 150-month intervals.

**Figure 6:**
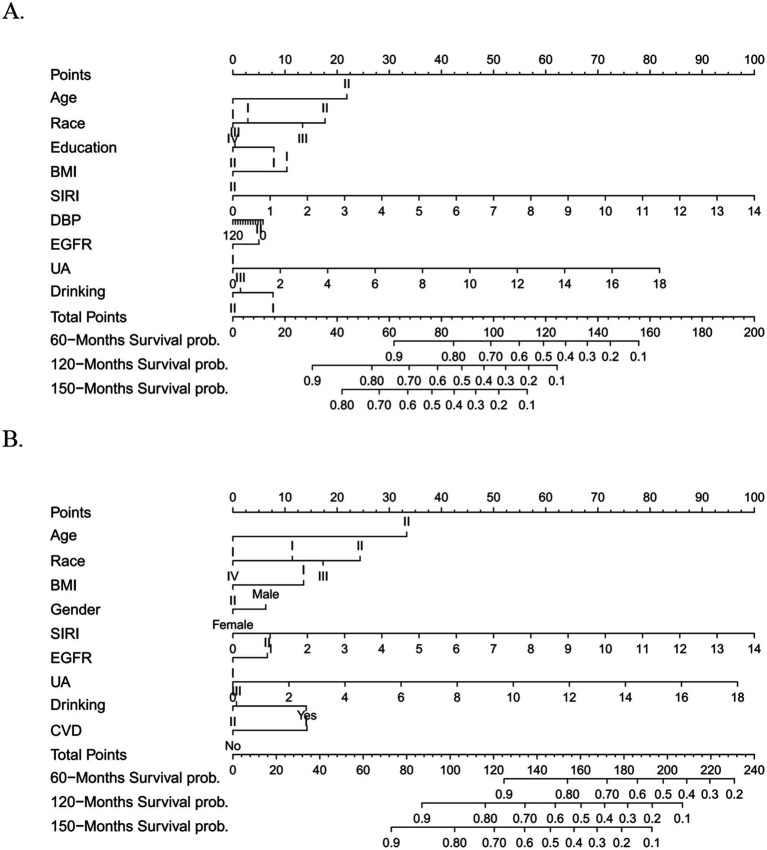
**(A)** Nomogram of the formed prognostic model in predicting all-cause mortality at 60 months, 120 months, 150 months time points. **(B)** Nomogram of the formed prognostic model in predicting cardiovascular mortality at 60 months, 120 months, 150 months time points.

Selected factors were synthesized to construct Cox analysis and comprehensive prognostic models, achieving AUC scores of 0.723, 0.726, and 0.753 for predicting all-cause mortality, and 0.721, 0.713, and 0.752 for predicting cardiovascular events at 60, 120, and 150 months, respectively (Figure 5). The C-index values through 1,000 resampling iterations were recorded as 0.6922 for all-cause mortality and 0.6911 for cardiovascular mortality at these time points. Nomograms were developed for 60-month, 120-month, and 150-month intervals to facilitate clinical application (for all-cause mortality: Figure 6A; for cardiovascular mortality: Figure 6B). Each nomogram assigned weighted points to individual predictors, enabling clinicians to calculate cumulative mortality risk for a given patient. The nomograms demonstrated excellent calibration via Hosmer–Lemeshow tests (*p* > 0.05 for all timepoints) and alignment between predicted and observed probabilities across validation and testing datasets. Decision curve analysis (DCA) of the predictive model for all-cause mortality displayed a broader threshold interval that surpassed the reference baseline, reflecting enhanced sensitivity in distinguishing risk levels and supporting the utility of the nomogram in risk stratification (Supplementary Figures S8, S9).

Two models were incorporated into eight machine learning methods to compare their predictive capabilities. The XGBoost method consistently achieved the highest AUC values and exhibited the most stable performance for both all-cause mortality (AUC = 0.796, 0.760, 0.747 at 60, 120, 150 months) and cardiovascular mortality (AUC = 0.732, 0.693, 0.693 at 60, 120, 150 months), with superior calibration across all evaluations (Supplementary Tables S6–S17 and Figures S10–S15).

An online prediction website was developed to provide real-time mortality risk assessment for CMM patients. The underlying algorithm will be dynamically refined and updated based on ongoing feedback. The tool is accessible at:

*All-cause mortality*: https://chenziq1.shinyapps.io/dynnomapp/;

*Cardiovascular mortality*: https://zhuaijing.shinyapps.io/dynnomapp/.

### Sensitivity analysis and validation

To confirm the robustness and generalizability of our findings, sensitivity analyses were conducted across three distinct cohorts. Participants who died within 2 years of follow-up were excluded, leaving 1,688 individuals in *Cohort 1* to reduce potential bias. Additionally, 145 participants from *NHANES (cycle 1999–2000, Cohort 2)* and a stratified random sample of 364 CMM patients from the Gaoyou population (*Cohort 3*) were selected to validate the predictive performance of the prognostic model.

In the fully adjusted Model 3, results from *Cohort 1* and *Cohort 2* confirmed that the differences in all-cause mortality between the highest SIRI group (Q4) and the reference group (Q1) persisted. The significant discrepancy in cardiovascular mortality observed in Cohort 1 was consistent with findings from the entire population (Supplementary Table S18, all *p* < 0.05). However, SIRI failed to independently predict specific mortality in Cohort 2 (Supplementary Table S19, *p* = 0.625), partly due to variations in follow-up time.

After an average follow-up period of 122 months, 125 deaths occurred among 145 patients in *Cohort 2*, half of which were attributed to cardiovascular events. In Cohort 3, with an average follow-up duration of 51.45 ± 7.02 months, 16 out of 364 CMM patients experienced death events. The survival group exhibited a mean SIRI value of 0.67 ± 0.61, significantly lower than the mean value of 0.88 ± 0.62 observed in the deceased group (Supplementary Table S20). Given the heterogeneity of the Gaoyou population, the variables “Ethnicity” and “PIR” were removed from the Cox analysis and predictive model. Despite differences in questionnaire variables and follow-up intervals between *Cohort 2* and *Cohort 3*, the SIRI exerted significant influence across quartiles (Supplementary Table S21) and model frameworks demonstrated remarkable predictive efficacy in both trials (all AUC > 0.7), achieving superior performance in predicting 150-month overall mortality [AUC = 0.775, 95% CI: 0.700–0.850] and 50-month cardiovascular mortality [AUC = 0.862, 95% CI: 0.795–0.928] (Supplementary Figure S16). Summary tables consolidating main performance metrics (C-index, AUC) across models with different indicators in three cohorts (NHANES 2001–2018, NHANES 1999–2000, Gaoyou cohort) were illustrated in Supplementary Tables S22–S24.

## Discussion

This study evaluated the prognostic impact of ten inflammatory and nutritional parameters—NLR, PLR, PNR, SII, SIRI, NHR, MHR, PHR, LHR, and NMR—on all-cause and cardiovascular mortality in patients with chronic multimorbidity (CMM). Among these indices, SIRI emerged as the most significant predictor across multiple variable selection methodologies, demonstrating a linear and significant association with increased mortality risk and consistent predictive value across various subgroups. The nomograms developed using Cox analysis, which incorporated SIRI and other accessible baseline covariates, achieved high discriminative performance and accuracy. Additionally, the XGBoost machine learning model provided superior applicability for predicting both mortality outcomes. Following rigorous internal and external validation, we confirmed the stability of these models and substantiated their clinical utility, offering a practical tool for individualized risk assessment.

Recent epidemiological surveys indicate that the prevalence of CMM in the United States increased from 9.4% in 1999 to 14.4% in 2018 (18). Combined studies from the United States and the European Union suggest that approximately 10 million adults suffer from CMM, highlighting the significant economic and health burdens associated with coexisting chronic diseases (19). A longitudinal study of 1,189,108 individuals found that a history of any two chronic conditions was associated with a 12-year reduction in life expectancy, while a history of all three conditions reduced life expectancy by 15 years (1). Despite the growing prevalence of CMM, our understanding of the complex interplay of risk factors, diagnostic paradigms, therapeutic strategies, and prognostic evaluations remains limited. This is partly due to the systematic exclusion of individuals with comorbid conditions in clinical trials and the predominant focus of observational research on isolated diseases (20–22). Furthermore, the physiological processes underlying CMM remain poorly understood, leaving a significant gap in mechanistic insights.

Inflammation and nutrition play a central role in CMM by promoting endothelial dysfunction, atherosclerosis, and endocrine imbalance. Inflammatory and nutritional factors bind to endothelial cell surface receptors, disrupting normal function through mechanisms such as impaired vasodilation, increased vascular permeability, procoagulant and antifibrinolytic states, leukocyte adhesion, and endothelial cell apoptosis (23, 24). Dysfunctional endothelial cells, in turn, become a platform for the recruitment, activation, and amplification of inflammatory cells, exacerbating tissue damage and microcirculatory disorders (25). Under inflammatory conditions, low-density lipoprotein accumulates in the arterial intima, undergoing modifications such as oxidation, lipolysis, proteolysis, and aggregation, which release phospholipids and induce hemodynamic strain (26). Inflammatory mediators activate multiple signaling pathways, interfere with the tyrosine phosphorylation of insulin receptor substrate (IRS), block insulin signal transduction, and reduce the sensitivity of muscle, adipose, and liver tissues to circulating insulin (27). These processes lead to pancreatic islet *β*-cell dysfunction and insulin resistance (28). Conversely, hyperglycemia itself promotes an inflammatory and nutritional state (29). Dietary nutrition, as modifiable and quantifiable lifestyle factors exerted influence on regulating systemic inflammation and immune function. A UK-based community longitudinal study verified that the low-inflammatory categorization derived from baseline inflammatory diet index (IDI) and empirical dietary inflammatory pattern (EDIP) determined reduced multimorbidity, slower accumulation of chronic diseases and prolonged chronic disease-free and multimorbidity-free survival time (30). Data from South Korean nutritional surveillance further indicated that CMM was inversely correlated with adequate intake of calcium, potassium, and fruits (31). In addition, another large-scale analysis of the NHANES database involving 13,178 participants revealed that antioxidant-rich dietary interventions attenuated the heightened risk of obesity-related CMM, particularly among older adults (32). Notably, inflammation has been proposed as a significant mediator in these relationships. Oxidative stress triggers a cascade of pathological changes, characterized by the aberrant proliferation of adipocytokines such as adiponectin, plasminogen activator-1, interleukin-6, and monocyte chemoattractant protein-1. In summary, nutritional status and inflammatory processes interacted synergistically to mediate disease onset and progression, thereby establishing a self-perpetuating pathological cycle (33–35).

The indicator SIRI, formulated from the quantification of neutrophils, monocytes, and lymphocytes in peripheral blood, emerges as an accessible biomarker for evaluating systemic inflammatory and nutritional dynamics (36). The reflection of a biologically meaningful balance between innate pro-inflammatory cells and adaptive immune competence may explain its strong association with adverse prognostic events in patients with cardiometabolic multimorbidity (CMM). Neutrophils are responsible for phagocytosing and eliminating pathogenic bacteria (37, 38). Circulating monocytes can infiltrate solid tissues and differentiate into diverse phenotypes, including dendritic cells, M1 macrophages, and M2 macrophages, in response to specific environmental cues (39). These specialized cells modulate immune activation, inflammatory regulation, and tissue repair. Meanwhile, lymphocytes play a critical role in adaptive immune responses, collaborating with other immune cells to orchestrate cell-mediated immunity (40). Mechanistically, elevated neutrophil and monocyte activity promotes atherogenesis and plaque instability through secretion of proteases, reactive oxygen species, and pro-inflammatory cytokines, and facilitates thrombogenic processes (41). In contrast, relative lymphopenia reflects impaired immunomodulation and inefficient resolution of inflammation, predisposing to chronic low-grade inflammation that contributes to insulin resistance, endothelial dysfunction, myocardial remodeling, and cerebrovascular injury (42). These processes collectively provide a plausible biological basis linking SIRI to the pathophysiology and deteriorated outcomes of CMM.

Numerous studies have explored the correlation between SIRI and common diseases. SIRI has been associated with the diagnosis of coronary artery disease (CAD), with significantly higher values observed in patients with acute coronary syndrome (ACS) compared to those with stable CAD (43). A study of 42,875 individuals found that elevated SIRI was associated with increased cardiovascular and all-cause mortality (44). Additionally, SIRI has been identified as an independent predictor of complication incidence and mortality in patients with DM (45, 46). However, many of these studies were conducted in homogeneous populations with a single cardiovascular disease diagnosis, limiting their generalizability to CMM, where cumulative inflammatory and nutritional burden and complex interactions between conditions may amplify prognostic significance.

Meantime, researches on the prognostic indicators and the development of risk prediction models for CMM patients remained limited. Dietary fish oil supplementation had been associated with the reduced mortality (47) and minimized cadmium exposure in CMM patients might contribute to the prevention of premature mortality (48). In terms of nutritional status, both the prevalence of CMM and the related mortality varied significantly according to the severity of abdominal obesity, independent of age, sex, and ethnicity (49). Insulin resistance serve as vital intermediate pathophysiological mechanism, and representative circulating biomarkers such as triglyceride-glucose (TyG) demonstrated predictive value for risk stratification (50). However, current studies frequently incorporated a limited number of covariates in survival analyses and lack validation across diverse cohorts. Although various anthropometric and nutritional factors had been clarified as independent effects, comprehensive variable selection through multiple analytical methods with adequately adjusted for a range of baseline characteristics had not been systematically implemented to confirm the robustness of these variables.

As the first prognostic models for CMM patients, the integrating SIRI into a nomogram complements traditional risk scores that focus on static cardiovascular risk factors (e.g., age, blood pressure, diabetes status) by adding a dynamic signal of systemic immune activation in practical application. This may improve risk discrimination and patient-level prognostication, particularly for individuals with similar traditional risk factor profile but diverse inflammatory burden such as exposing to daily high-inflammatory diet or suffering exhaustion after long-term work. And the nomogram is intended as a supplemental clinical tool utilized in (i) identifying high-risk patients who could benefit from more intensive monitoring or anti-inflammatory strategies, (ii) improving individualized shared-decision discussions, and (iii) serving as a simple trigger for further biomarker or imaging evaluation (51, 52). Future research should focus on validating these findings in larger, multicenter cohorts and exploring the mechanistic links between specific pathways and CMM progression (53, 54).

This study has several limitations. First, its retrospective design may introduce selection bias and limit the generalizability of the findings. And included two cohorts differed in ethnicity, healthcare systems, and data collection protocols, which might introduce residual heterogeneity despite standardized definitions and calibration procedures. Second, potential confounders such as health insurance status may have been omitted due to incomplete data and lack of clear definition standards. Third, the analyses relied on the single baseline measurement of inflammatory and nutritional markers on account of the cross-sectional attributes of databases without repeated measurements during follow-up. Because inflammatory biomarkers would fluctuate over time due to transient physiological, pharmacological or environmental effects, this potentially lead to exposure misclassification and underestimation consequently. Finally, while the nomograms demonstrated strong predictive efficacy, more external validation is required to confirm their applicability across diverse populations. And we the relatively small number of participants of the external validation cohort (*n* = 364) might limit the statistical efficacy and increase uncertainty of the cox regression-based models. Future studies with harmonized data collection, longitudinal designs or repeated-measures analyses are warranted to validate the temporal stability of these associations and to clarify potential causal pathways.

## Conclusion

This study underscores the prognostic significance of inflammatory and nutritional parameters, particularly the SIRI, in patients with CMM. The developed nomograms and dynamic online model provide a practical and accessible tool for individualized risk assessment and clinical decision-making, highlighting the critical role of systemic inflammation in CMM prognosis. Future research should focus on validating these findings in larger, diverse cohorts and exploring targeted therapeutic strategies to improve outcomes in this high-risk population.

## Data Availability

The raw data supporting the conclusions of this article will be made available by the authors, without undue reservation.
