# Supplementary material for: Impact of inflammatory and nutritional parameters on mortality in cardiovascular multimorbidity: a comprehensive prognostic analysis based on two datasets
Source: Front Nutr. 2025 Nov 21;12:1702364. doi: 10.3389/fnut.2025.1702364 (PMC12679892; doi:10.3389/fnut.2025.1702364)
Supplement: Supplementary file 1 [file Supplementary_file_1.docx]

**SUPPLEMENTARY MATERIALS**

**TABLE OF CONTENTS**

Supplementary Table S1...........................................................................................................................2

Supplementary Table S2...........................................................................................................................3

Supplementary Table S3...........................................................................................................................5

Supplementary Table S4...........................................................................................................................6

Supplementary Table S5...........................................................................................................................7

Supplementary Table S6...........................................................................................................................8

Supplementary Table S7...........................................................................................................................8

Supplementary Table S8...........................................................................................................................9

Supplementary Table S9...........................................................................................................................9

Supplementary Table S10........................................................................................................................10

Supplementary Table S11........................................................................................................................11

Supplementary Table S12........................................................................................................................11

Supplementary Table S13........................................................................................................................12

Supplementary Table S14........................................................................................................................12

Supplementary Table S15........................................................................................................................13

Supplementary Table S16........................................................................................................................14

Supplementary Table S17........................................................................................................................14

Supplementary Table S18........................................................................................................................16

Supplementary Table S19........................................................................................................................17

Supplementary Table S20........................................................................................................................18

Supplementary Table S21........................................................................................................................20

Supplementary Table S22........................................................................................................................20

Supplementary Table S23........................................................................................................................21

Supplementary Table S24........................................................................................................................22

Supplementary Table S25........................................................................................................................23

Supplementary Figure S1........................................................................................................................24

Supplementary Figure S2........................................................................................................................27

Supplementary Figure S3........................................................................................................................28

Supplementary Figure S4........................................................................................................................29

Supplementary Figure S5........................................................................................................................30

Supplementary Figure S6........................................................................................................................31

Supplementary Figure S7........................................................................................................................24

Supplementary Figure S8........................................................................................................................30

Supplementary Figure S9........................................................................................................................34

Supplementary Figure S10......................................................................................................................35

Supplementary Figure S11......................................................................................................................36

Supplementary Figure S12......................................................................................................................37

Supplementary Figure S13......................................................................................................................38

Supplementary Figure S14......................................................................................................................39

Supplementary Figure S15......................................................................................................................40

Supplementary Figure S16......................................................................................................................41

| **Table S1:** Measurement formulas of Inflammatory and Nutritional Indicators. | |
| --- | --- |
| Neutrophil-to-Lymphocyte Ratio (NLR) | Neutrophil count (10^9^/L) / Lymphocyte count (10^9^/L) |
| Platelet-to-Lymphocyte Ratio (PLR) | Platelet count (10^9^/L) / Lymphocyte count (10^9^/L) |
| Platelet-to-Neutrophil Ratio (PNR) | Platelet count (10^9^/L) / Neutrophil count (10^9^/L) |
| Systemic Immune-Inflammation Index (SII) | Neutrophil count (10^9^/L) / Lymphocyte count (10^9^/L) |
| Systemic Inflammation Response Index (SIRI) | Neutrophil count (10^9^/L) × Monocyte count (10^9^/L) / Lymphocyte count (10^9^/L) |
| Neutrophil-to-High-Density  Lipoprotein Cholesterol Ratio (NHR) | Neutrophil count (10^9^/L) / HDL-C (mg/dL) |
| Monocyte-to-High-Density  Lipoprotein Cholesterol Ratio (MHR) | Monocyte count (10^9^/L) / HDL-C (mg/dL) |
| Platelet-to-High-Density  Lipoprotein Cholesterol Ratio (PHR) | Platelet count (10^9^/L) / HDL-C (mg/dL) |
| Lymphocyte-to-High-Density  Lipoprotein Cholesterol Ratio (LHR) | Lymphocyte count (10^9^/L) / HDL-C (mg/dL) |
| Neutrophil-to-Monocyte Ratio (NMR) | Neutrophil count (10^9^/L) / Monocyte count (10^9^/L) |

| **Table S2:** Variance inflation factor analysis of ten inflammatory and nutritional indicators with selected covariates for all-cause mortality. | | | | | | | | | |
| --- | --- | --- | --- | --- | --- | --- | --- | --- | --- |
|  | **VIF** |  | **VIF** |  | **VIF** |  | **VIF** |  | **VIF** |
| NLR | 1.07 | PLR | 1.02 | PNR | 1.09 | SII | 1.04 | SIRI | 1.10 |
| Gender | 1.28 | Gender | 1.28 | Gender | 1.29 | Gender | 1.27 | Gender | 1.28 |
| Age | 1.33 | Age | 1.32 | Age | 1.32 | Age | 1.32 | Age | 1.32 |
| Ethnicity | 1.07 | Ethnicity | 1.08 | Ethnicity | 1.09 | Ethnicity | 1.07 | Ethnicity | 1.06 |
| Education | 1.21 | Education | 1.20 | Education | 1.19 | Education | 1.20 | Education | 1.20 |
| PIR | 1.23 | PIR | 1.22 | PIR | 1.22 | PIR | 1.22 | PIR | 1.22 |
| SBP | 1.25 | SBP | 1.25 | SBP | 1.23 | SBP | 1.25 | SBP | 1.24 |
| DBP | 1.23 | DBP | 1.21 | DBP | 1.21 | DBP | 1.21 | DBP | 1.22 |
| eGFR | 1.06 | eGFR | 1.05 | eGFR | 1.05 | eGFR | 1.05 | eGFR | 1.05 |
| BMI | 1.12 | BMI | 1.12 | BMI | 1.12 | BMI | 1.12 | BMI | 1.12 |
| Uric acid | 1.09 | Uric acid | 1.09 | Uric acid | 1.09 | Uric acid | 1.09 | Uric acid | 1.09 |
| HbA1c | 1.23 | HbA1c | 1.23 | HbA1c | 1.23 | HbA1c | 1.23 | HbA1c | 1.23 |
| HDL-C | 1.23 | HDL-C | 1.28 | HDL-C | 1.24 | HDL-C | 1.23 | HDL-C | 1.22 |
| TC | 1.20 | TC | 1.20 | TC | 1.21 | TC | 1.19 | TC | 1.19 |
| Smoking | 1.16 | Smoking | 1.16 | Smoking | 1.16 | Smoking | 1.17 | Smoking | 1.17 |
| Drinking | 1.11 | Drinking | 1.12 | Drinking | 1.10 | Drinking | 1.11 | Drinking | 1.12 |
| Hypertension | 1.17 | Hypertension | 1.18 | Hypertension | 1.17 | Hypertension | 1.17 | Hypertension | 1.17 |
| Diabetes | 1.26 | Diabetes | 1.26 | Diabetes | 1.26 | Diabetes | 1.26 | Diabetes | 1.26 |
| Liver disease | 1.04 | Liver disease | 1.04 | Liver disease | 1.04 | Liver disease | 1.04 | Liver disease | 1.03 |
| Cancer | 1.05 | Cancer | 1.04 | Cancer | 1.05 | Cancer | 1.05 | Cancer | 1.04 |
| CVD | 1.06 | CVD | 1.05 | CVD | 1.06 | CVD | 1.05 | CVD | 1.07 |

|  | **VIF** |  | **VIF** |  | **VIF** |  | **VIF** |  | **VIF** |
| --- | --- | --- | --- | --- | --- | --- | --- | --- | --- |
| NHR | 1.68 | MHR | 1.71 | PHR | 1.75 | LHR | 1.58 | NMR | 1.06 |
| Gender | 1.27 | Gender | 1.29 | Gender | 1.33 | Gender | 1.31 | Gender | 1.30 |
| Age | 1.32 | Age | 1.31 | Age | 1.33 | Age | 1.33 | Age | 1.32 |
| Ethnicity | 1.08 | Ethnicity | 1.07 | Ethnicity | 1.08 | Ethnicity | 1.08 | Ethnicity | 1.08 |
| Education | 1.20 | Education | 1.20 | Education | 1.19 | Education | 1.20 | Education | 1.19 |
| PIR | 1.22 | PIR | 1.22 | PIR | 1.22 | PIR | 1.22 | PIR | 1.23 |
| SBP | 1.24 | SBP | 1.24 | SBP | 1.25 | SBP | 1.26 | SBP | 1.25 |
| DBP | 1.21 | DBP | 1.21 | DBP | 1.21 | DBP | 1.22 | DBP | 1.21 |
| eGFR | 1.05 | eGFR | 1.05 | eGFR | 1.05 | eGFR | 1.05 | eGFR | 1.05 |
| BMI | 1.13 | BMI | 1.13 | BMI | 1.12 | BMI | 1.12 | BMI | 1.12 |
| Uric acid | 1.10 | Uric acid | 1.09 | Uric acid | 1.09 | Uric acid | 1.10 | Uric acid | 1.09 |
| HbA1c | 1.23 | HbA1c | 1.23 | HbA1c | 1.23 | HbA1c | 1.23 | HbA1c | 1.23 |
| HDL-C | 1.79 | HDL-C | 1.85 | HDL-C | 1.98 | HDL-C | 1.76 | HDL-C | 1.24 |
| TC | 1.20 | TC | 1.20 | TC | 1.21 | TC | 1.19 | TC | 1.20 |
| Smoking | 1.21 | Smoking | 1.17 | Smoking | 1.18 | Smoking | 1.17 | Smoking | 1.18 |
| Drinking | 1.12 | Drinking | 1.12 | Drinking | 1.11 | Drinking | 1.11 | Drinking | 1.11 |
| Hypertension | 1.17 | Hypertension | 1.17 | Hypertension | 1.18 | Hypertension | 1.18 | Hypertension | 1.17 |
| Diabetes | 1.27 | Diabetes | 1.26 | Diabetes | 1.11 | Diabetes | 1.25 | Diabetes | 1.26 |
| Liver disease | 1.04 | Liver disease | 1.04 | Liver disease | 1.04 | Liver disease | 1.04 | Liver disease | 1.04 |
| Cancer | 1.05 | Cancer | 1.05 | Cancer | 1.05 | Cancer | 1.05 | Cancer | 1.05 |
| CVD | 1.06 | CVD | 1.05 | CVD | 1.06 | CVD | 1.06 | CVD | 1.05 |

| **Table S3:** Variance inflation factor analysis of ten inflammatory and nutritional indicators with selected covariates for cardiovascular mortality. | | | | | | | | | |
| --- | --- | --- | --- | --- | --- | --- | --- | --- | --- |
|  | **VIF** |  | **VIF** |  | **VIF** |  | **VIF** |  | **VIF** |
| NLR | 1.08 | PLR | 1.02 | PNR | 1.09 | SII | 1.04 | SIRI | 1.11 |
| Gender | 1.28 | Gender | 1.28 | Gender | 1.30 | Gender | 1.28 | Gender | 1.30 |
| Age | 1.29 | Age | 1.28 | Age | 1.28 | Age | 1.28 | Age | 1.28 |
| Ethnicity | 1.08 | Ethnicity | 1.08 | Ethnicity | 1.09 | Ethnicity | 1.08 | Ethnicity | 1.07 |
| Education | 1.22 | Education | 1.21 | Education | 1.21 | Education | 1.21 | Education | 1.21 |
| PIR | 1.23 | PIR | 1.22 | PIR | 1.22 | PIR | 1.23 | PIR | 1.22 |
| SBP | 1.26 | SBP | 1.29 | SBP | 1.24 | SBP | 1.25 | SBP | 1.25 |
| DBP | 1.22 | DBP | 1.21 | DBP | 1.21 | DBP | 1.21 | DBP | 1.22 |
| eGFR | 1.06 | eGFR | 1.05 | eGFR | 1.05 | eGFR | 1.05 | eGFR | 1.05 |
| BMI | 1.12 | BMI | 1.12 | BMI | 1.12 | BMI | 1.12 | BMI | 1.12 |
| Uric acid | 1.09 | Uric acid | 1.09 | Uric acid | 1.09 | Uric acid | 1.09 | Uric acid | 1.09 |
| HbA1c | 1.25 | HbA1c | 1.28 | HbA1c | 1.25 | HbA1c | 1.25 | HbA1c | 1.26 |
| HDL-C | 1.23 | HDL-C | 1.23 | HDL-C | 1.24 | HDL-C | 1.23 | HDL-C | 1.22 |
| TC | 1.21 | TC | 1.20 | TC | 1.22 | TC | 1.20 | TC | 1.20 |
| Smoking | 1.14 | Smoking | 1.15 | Smoking | 1.15 | Smoking | 1.15 | Smoking | 1.15 |
| Drinking | 1.09 | Drinking | 1.08 | Drinking | 1.09 | Drinking | 1.09 | Drinking | 1.10 |
| Hypertension | 1.18 | Hypertension | 1.18 | Hypertension | 1.18 | Hypertension | 1.18 | Hypertension | 1.18 |
| Diabetes | 1.28 | Diabetes | 1.28 | Diabetes | 1.28 | Diabetes | 1.28 | Diabetes | 1.29 |
| Liver disease | 1.02 | Liver disease | 1.03 | Liver disease | 1.02 | Liver disease | 1.02 | Liver disease | 1.02 |
| Cancer | 1.04 | Cancer | 1.04 | Cancer | 1.04 | Cancer | 1.04 | Cancer | 1.04 |
| CVD | 1.05 | CVD | 1.05 | CVD | 1.05 | CVD | 1.05 | CVD | 1.06 |

|  | **VIF** |  | **VIF** |  | **VIF** |  | **VIF** |  | **VIF** |
| --- | --- | --- | --- | --- | --- | --- | --- | --- | --- |
| NHR | 1.67 | MHR | 1.70 | PHR | 1.74 | LHR | 1.55 | NMR | 1.06 |
| Gender | 1.28 | Gender | 1.29 | Gender | 1.34 | Gender | 1.32 | Gender | 1.30 |
| Age | 1.28 | Age | 1.28 | Age | 1.29 | Age | 1.30 | Age | 1.28 |
| Ethnicity | 1.09 | Ethnicity | 1.08 | Ethnicity | 1.08 | Ethnicity | 1.08 | Ethnicity | 1.08 |
| Education | 1.21 | Education | 1.21 | Education | 1.20 | Education | 1.21 | Education | 1.21 |
| PIR | 1.22 | PIR | 1.22 | PIR | 1.22 | PIR | 1.23 | PIR | 1.23 |
| SBP | 1.25 | SBP | 1.25 | SBP | 1.26 | SBP | 1.26 | SBP | 1.25 |
| DBP | 1.21 | DBP | 1.20 | DBP | 1.21 | DBP | 1.21 | DBP | 1.20 |
| eGFR | 1.05 | eGFR | 1.04 | eGFR | 1.05 | eGFR | 1.05 | eGFR | 1.05 |
| BMI | 1.12 | BMI | 1.12 | BMI | 1.12 | BMI | 1.12 | BMI | 1.12 |
| Uric acid | 1.10 | Uric acid | 1.09 | Uric acid | 1.09 | Uric acid | 1.09 | Uric acid | 1.09 |
| HbA1c | 1.25 | HbA1c | 1.25 | HbA1c | 1.25 | HbA1c | 1.23 | HbA1c | 1.25 |
| HDL-C | 1.77 | HDL-C | 1.85 | HDL-C | 1.98 | HDL-C | 1.74 | HDL-C | 1.24 |
| TC | 1.21 | TC | 1.21 | TC | 1.23 | TC | 1.21 | TC | 1.21 |
| Smoking | 1.19 | Smoking | 1.15 | Smoking | 1.15 | Smoking | 1.15 | Smoking | 1.16 |
| Drinking | 1.10 | Drinking | 1.10 | Drinking | 1.09 | Drinking | 1.10 | Drinking | 1.09 |
| Hypertension | 1.18 | Hypertension | 1.18 | Hypertension | 1.18 | Hypertension | 1.18 | Hypertension | 1.18 |
| Diabetes | 1.28 | Diabetes | 1.28 | Diabetes | 1.28 | Diabetes | 1.27 | Diabetes | 1.28 |
| Liver disease | 1.02 | Liver disease | 1.02 | Liver disease | 1.02 | Liver disease | 1.02 | Liver disease | 1.02 |
| Cancer | 1.04 | Cancer | 1.04 | Cancer | 1.04 | Cancer | 1.04 | Cancer | 1.04 |
| CVD | 1.05 | CVD | 1.05 | CVD | 1.05 | CVD | 1.05 | CVD | 1.05 |

**Table S4:** Cox proportional hazards analysis of inflammatory and nutritional parameters for all-cause and cardiovascular mortality in unadjusted model.

| **Subgroup** | **Quartile** | | | | **P for trend** |
| --- | --- | --- | --- | --- | --- |
|  | Q1 | Q2 | Q3 | Q4 |  |
| **All-cause mortality** | | | | | |
| **NLR** | Reference | 1.26 (1.02 ~ 1.55) | 1.52 (1.23 ~ 1.87) | 2.70 (2.22 ~ 3.29) | <.001 |
| **PLR** | Reference | 1.01 (0.82 ~ 1.23) | 1.05 (0.86 ~ 1.28) | 1.32 (1.09 ~ 1.59) | 0.003 |
| **PNR** | Reference | 0.62 (0.51 ~ 0.75) | 0.58 (0.48 ~ 0.70) | 0.43 (0.36 ~ 0.52) | <.001 |
| **SII** | Reference | 1.15 (0.94 ~ 1.42) | 1.29 (1.06 ~ 1.58) | 1.79 (1.48 ~ 2.18) | <.001 |
| **SIRI** | Reference | 1.33 (1.07 ~ 1.64) | 1.84 (1.50 ~ 2.26) | 3.08 (2.53 ~ 3.75) | <.001 |
| **NHR** | Reference | 1.08 (0.89 ~ 1.32) | 1.25 (1.02 ~ 1.52) | 1.49 (1.23 ~ 1.81) | <.001 |
| **MHR** | Reference | 1.13 (0.93 ~ 1.38) | 1.46 (1.20 ~ 1.77) | 1.43 (1.18 ~ 1.74) | <.001 |
| **PHR** | Reference | 0.94 (0.78 ~ 1.13) | 0.69 (0.57 ~ 0.84) | 0.67 (0.56 ~ 0.82) | <.001 |
| **LHR** | Reference | 0.76 (0.63 ~ 0.91) | 0.58 (0.48 ~ 0.70) | 0.58 (0.48 ~ 0.70) | <.001 |
| **NMR** | Reference | 1.05 (0.87 ~ 1.28) | 0.91 (0.75 ~ 1.10) | 1.02 (0.85 ~ 1.24) | 0.812 |
| **Cardiovascular mortality** | | | | | |
| **NLR** | Reference | 1.39 (1.01 ~ 1.92) | 1.39 (1.00 ~ 1.93) | 2.46 (1.80 ~ 3.37) | <.001 |
| **PLR** | Reference | 0.92 (0.66 ~ 1.27) | 1.05 (0.76 ~ 1.44) | 1.42 (1.06 ~ 1.90) | 0.009 |
| **PNR** | Reference | 0.69 (0.51 ~ 0.93) | 0.65 (0.49 ~ 0.88) | 0.57 (0.42 ~ 0.77) | <.001 |
| **SII** | Reference | 1.16 (0.85 ~ 1.59) | 1.16 (0.85 ~ 1.59) | 1.51 (1.11 ~ 2.04) | 0.011 |
| **SIRI** | Reference | 1.73 (1.25 ~ 2.40) | 1.88 (1.35 ~ 2.62) | 2.87 (2.08 ~ 3.97) | <.001 |
| **NHR** | Reference | 1.05 (0.77 ~ 1.41) | 1.22 (0.91 ~ 1.65) | 1.08 (0.79 ~ 1.48) | 0.396 |
| **MHR** | Reference | 1.26 (0.93 ~ 1.70) | 1.32 (0.97 ~ 1.80) | 1.35 (0.99 ~ 1.84) | 0.055 |
| **PHR** | Reference | 0.99 (0.74 ~ 1.31) | 0.62 (0.45 ~ 0.85) | 0.67 (0.50 ~ 0.91) | <.001 |
| **LHR** | Reference | 0.76 (0.58 ~ 1.01) | 0.56 (0.41 ~ 0.75) | 0.54 (0.40 ~ 0.73) | <.001 |
| **NMR** | Reference | 1.10 (0.82 ~ 1.47) | 0.87 (0.65 ~ 1.17) | 0.80 (0.59 ~ 1.09) | 0.068 |

Unadjusted

Note: NLR, Neutrophil-to-Lymphocyte Ratio; PLR, Platelet-to-Lymphocyte Ratio; PNR, Platelet-to-Neutrophil Ratio; SII, Systemic Immune-Inflammation Index; SIRI, Systemic Inflammation Response Index; NHR, Neutrophil-to-High-Density Lipoprotein Ratio; MHR, Monocyte-to-High-Density Lipoprotein Ratio; PHR, Platelet-to-High-Density Lipoprotein Ratio; LHR, Lymphocyte-to-High-Density Lipoprotein Ratio; NMR, Neutrophil-to-Monocyte Ratio.

**Table S5:** Cox proportional hazards analysis of inflammatory and nutritional parameters for all-cause and cardiovascular mortality in partly-adjusted model.

| **Subgroup** | **Quartile** | | | | **P for trend** |
| --- | --- | --- | --- | --- | --- |
|  | Q1 | Q2 | Q3 | Q4 |  |
| **All-cause mortality** | | | | | |
| **NLR** | Reference | 1.08 (0.87 ~ 1.34) | 1.25 (1.01 ~ 1.56) | 2.10 (1.70 ~ 2.60) | <.001 |
| **PLR** | Reference | 1.05 (0.85 ~ 1.29) | 1.13 (0.92 ~ 1.38) | 1.23 (1.01 ~ 1.49) | 0.026 |
| **PNR** | Reference | 0.67 (0.55 ~ 0.81) | 0.63 (0.52 ~ 0.76) | 0.52 (0.42 ~ 0.64) | <.001 |
| **SII** | Reference | 1.07 (0.87 ~ 1.32) | 1.15 (0.94 ~ 1.42) | 1.64 (1.34 ~ 2.00) | <.001 |
| **SIRI** | Reference | 1.16 (0.93 ~ 1.46) | 1.47 (1.19 ~ 1.83) | 2.34 (1.88 ~ 2.90) | <.001 |
| **NHR** | Reference | 1.17 (0.94 ~ 1.46) | 1.45 (1.15 ~ 1.84) | 1.85 (1.43 ~ 2.40) | <.001 |
| **MHR** | Reference | 1.12 (0.90 ~ 1.39) | 1.50 (1.19 ~ 1.88) | 1.38 (1.07 ~ 1.78) | <.001 |
| **PHR** | Reference | 0.85 (0.70 ~ 1.04) | 0.70 (0.56 ~ 0.88) | 0.67 (0.52 ~ 0.87) | <.001 |
| **LHR** | Reference | 0.74 (0.61 ~ 0.89) | 0.59 (0.48 ~ 0.73) | 0.55 (0.43 ~ 0.70) | <.001 |
| **NMR** | Reference | 1.11 (0.91 ~ 1.35) | 1.01 (0.83 ~ 1.23) | 1.22 (1.00 ~ 1.48) | 0.129 |
| **Cardiovascular mortality** | | | | | |
| **NLR** | Reference | 1.15 (0.82 ~ 1.61) | 1.10 (0.78 ~ 1.56) | 1.87 (1.34 ~ 2.62) | <.001 |
| **PLR** | Reference | 0.99 (0.71 ~ 1.37) | 1.17 (0.85 ~ 1.61) | 1.37 (1.01 ~ 1.85) | 0.020 |
| **PNR** | Reference | 0.79 (0.58 ~ 1.08) | 0.74 (0.55 ~ 1.01) | 0.75 (0.54 ~ 1.04) | 0.070 |
| **SII** | Reference | 1.04 (0.76 ~ 1.43) | 1.02 (0.74 ~ 1.41) | 1.40 (1.02 ~ 1.91) | 0.045 |
| **SIRI** | Reference | 1.47 (1.04 ~ 2.07) | 1.45 (1.03 ~ 2.06) | 2.09 (1.47 ~ 2.98) | <.001 |
| **NHR** | Reference | 1.00 (0.72 ~ 1.39) | 1.18 (0.83 ~ 1.68) | 1.03 (0.68 ~ 1.56) | 0.662 |
| **MHR** | Reference | 1.17 (0.84 ~ 1.62) | 1.21 (0.84 ~ 1.73) | 1.10 (0.74 ~ 1.64) | 0.691 |
| **PHR** | Reference | 0.85 (0.62 ~ 1.16) | 0.60 (0.42 ~ 0.87) | 0.63 (0.42 ~ 0.94) | 0.008 |
| **LHR** | Reference | 0.74 (0.55 ~ 0.99) | 0.57 (0.41 ~ 0.80) | 0.48 (0.33 ~ 0.70) | <.001 |
| **NMR** | Reference | 1.12 (0.83 ~ 1.50) | 0.96 (0.71 ~ 1.31) | 0.95 (0.69 ~ 1.31) | 0.573 |

Adjusted for gender, age, ethnicity, education, and poverty income ratio.

Note: NLR, Neutrophil-to-Lymphocyte Ratio; PLR, Platelet-to-Lymphocyte Ratio; PNR, Platelet-to-Neutrophil Ratio; SII, Systemic Immune-Inflammation Index; SIRI, Systemic Inflammation Response Index; NHR, Neutrophil-to-High-Density Lipoprotein Ratio; MHR, Monocyte-to-High-Density Lipoprotein Ratio; PHR, Platelet-to-High-Density Lipoprotein Ratio; LHR, Lymphocyte-to-High-Density Lipoprotein Ratio; NMR, Neutrophil-to-Monocyte Ratio.

| **Table S6:** Subsequent variable selection for models integrating SIRI and baseline covariates (via Boruta, Lasso, and stepwise regression) | | |
| --- | --- | --- |
| **All-cause mortality** | | |
| Boruta | LASSO | Cox |
| Age  SIRI  UA  SBP  DBP  BMI  Ethnicity  TC  HbA1c  Drinking  EGFR | Age  SIRI  BMI  UA  Cancer  Education  EGFR  Diabetes  Drinking  CVD  Ethnicity  Smoking  DBP | Age  Education  Ethnicity  BMI  PIR  EGFR  SIRI  UA |
| **Cardiovascular mortality** | | |
| Boruta | LASSO | Cox |
| Age  SIRI  UA  BMI  EGFR  CVD  Drinking | Age  BMI  CVD  SIRI  Cancer  UA  Education  HbA1c  Drinking  Gender  Ethnicity  EGFR  PIR  DBP  Diabetes  TC | Gender  Age  Ethnicity  SIRI  UA  HbA1c |

| **Table S7:** Different machine learning models’ parameters of ROC curves in training dateset of the predictive model for all- mortality at 60 months. | | | | | | | | | |
| --- | --- | --- | --- | --- | --- | --- | --- | --- | --- |
| **Models** | **AUC**  **(SD)** | **Cutoff**  **(SD)** | **Accuracy**  **(SD)** | **Sensitivity**  **(SD)** | **Specificity**  **(SD)** | **Positive Predictive Value (SD)** | **Passive Predictive Value Value(SD)** | **F1**  **(SD)** | **Kappa**  **(SD)** |
| XGBoost | 0.796(0.006) | 0.511(0.018) | 0.731(0.009) | 0.689(0.050) | 0.770(0.030) | 0.742(0.008) | 0.723(0.022) | 0.713(0.023) | 0.460(0.019) |
| logistic | 0.742(0.007) | 0.445(0.022) | 0.683(0.003) | 0.758(0.032) | 0.611(0.035) | 0.651(0.014) | 0.727(0.018) | 0.700(0.005) | 0.368(0.004) |
| LightGBM | 0.789(0.007) | 0.393(0.044) | 0.753(0.000) | 0.818(0.028) | 0.691(0.026) | 0.717(0.013) | 0.800(0.021) | 0.764(0.005) | 0.507(0.001) |
| RandomForest | 0.738(0.004) | 0.479(0.012) | 0.678(0.000) | 0.745(0.070) | 0.615(0.066) | 0.651(0.021) | 0.721(0.038) | 0.692(0.018) | 0.359(0.002) |
| AdaBoost | 0.791(0.008) | 0.500(0.002) | 0.720(0.006) | 0.704(0.053) | 0.735(0.039) | 0.719(0.011) | 0.724(0.022) | 0.710(0.021) | 0.439(0.013) |
| DecisionTree | 0.732(0.000) | 0.493(0.002) | 0.665(0.001) | 0.716(0.025) | 0.615(0.022) | 0.640(0.002) | 0.695(0.008) | 0.676(0.010) | 0.331(0.003) |
| MLP | 0.722(0.001) | 0.493(0.010) | 0.676(0.007) | 0.731(0.044) | 0.624(0.054) | 0.652(0.023) | 0.710(0.019) | 0.688(0.007) | 0.354(0.012) |
| SVM | 0.669(0.018) | 0.533(0.000) | 0.635(0.007) | 0.640(0.035) | 0.629(0.048) | 0.624(0.014) | 0.647(0.001) | 0.631(0.010) | 0.269(0.014) |

| **Table S8:** Different machine learning models’ parameters of ROC curves in validation dateset of the predictive model for all- mortality at 60 months. | | | | | | | | | |
| --- | --- | --- | --- | --- | --- | --- | --- | --- | --- |
| **Models** | **AUC**  **(SD)** | **Cutoff**  **(SD)** | **Accuracy**  **(SD)** | **Sensitivity**  **(SD)** | **Specificity**  **(SD)** | **Positive Predictive Value (SD)** | **Passive Predictive Value Value(SD)** | **F1**  **(SD)** | **Kappa**  **(SD)** |
| XGBoost | 0.755(0.041) | 0.511(0.018) | 0.677(0.036) | 0.665(0.016) | 0.689(0.078) | 0.637(0.067) | 0.717(0.001) | 0.648(0.028) | 0.351(0.067) |
| logistic | 0.740(0.031) | 0.445(0.022) | 0.638(0.003) | 0.797(0.047) | 0.507(0.040) | 0.566(0.009) | 0.760(0.017) | 0.662(0.023) | 0.293(0.008) |
| LightGBM | 0.639(0.052) | 0.393(0.044) | 0.614(0.027) | 0.699(0.023) | 0.545(0.034) | 0.553(0.012) | 0.691(0.043) | 0.618(0.016) | 0.237(0.054) |
| RandomForest | 0.715(0.024) | 0.479(0.012) | 0.644(0.015) | 0.729(0.090) | 0.572(0.049) | 0.578(0.017) | 0.732(0.038) | 0.643(0.046) | 0.294(0.038) |
| AdaBoost | 0.746(0.035) | 0.500(0.002) | 0.671(0.048) | 0.692(0.017) | 0.657(0.099) | 0.626(0.077) | 0.724(0.007) | 0.653(0.035) | 0.345(0.087) |
| DecisionTree | 0.668(0.030) | 0.493(0.002) | 0.611(0.012) | 0.727(0.051) | 0.520(0.057) | 0.551(0.027) | 0.704(0.029) | 0.624(0.001) | 0.239(0.011) |
| MLP | 0.733(0.037) | 0.493(0.010) | 0.644(0.003) | 0.776(0.081) | 0.533(0.077) | 0.574(0.000) | 0.757(0.032) | 0.658(0.029) | 0.300(0.002) |
| SVM | 0.661(0.014) | 0.533(0.000) | 0.632(0.015) | 0.685(0.023) | 0.589(0.011) | 0.573(0.000) | 0.699(0.032) | 0.624(0.010) | 0.269(0.031) |

| **Table S9:** Different machine learning models’ parameters of ROC curves in training dateset of the predictive model for all- mortality at 120 months. | | | | | | | | | |
| --- | --- | --- | --- | --- | --- | --- | --- | --- | --- |
| **Models** | **AUC**  **(SD)** | **Cutoff**  **(SD)** | **Accuracy**  **(SD)** | **Sensitivity**  **(SD)** | **Specificity**  **(SD)** | **Positive Predictive Value (SD)** | **Passive Predictive Value Value(SD)** | **F1**  **(SD)** | **Kappa**  **(SD)** |
| XGBoost | 0.760(0.004) | 0.472(0.004) | 0.700(0.003) | 0.714(0.006) | 0.688(0.010) | 0.673(0.004) | 0.728(0.000) | 0.692(0.001) | 0.401(0.005) |
| logistic | 0.707(0.001) | 0.469(0.012) | 0.657(0.002) | 0.683(0.010) | 0.633(0.005) | 0.625(0.001) | 0.690(0.006) | 0.653(0.004) | 0.315(0.005) |
| LightGBM | 0.585(0.030) | 0.608(0.044) | 0.599(0.006) | 0.798(0.011) | 0.422(0.000) | 0.553(0.005) | 0.699(0.011) | 0.653(0.007) | 0.214(0.011) |
| RandomForest | 0.712(0.004) | 0.477(0.013) | 0.654(0.011) | 0.715(0.057) | 0.599(0.072) | 0.618(0.025) | 0.703(0.018) | 0.661(0.010) | 0.311(0.017) |
| AdaBoost | 0.753(0.005) | 0.497(0.000) | 0.692(0.001) | 0.755(0.004) | 0.636(0.006) | 0.650(0.002) | 0.743(0.000) | 0.699(0.001) | 0.388(0.002) |
| DecisionTree | 0.727(0.003) | 0.503(0.003) | 0.668(0.002) | 0.661(0.014) | 0.675(0.016) | 0.646(0.005) | 0.689(0.003) | 0.653(0.004) | 0.335(0.002) |
| MLP | 0.699(0.001) | 0.470(0.002) | 0.656(0.006) | 0.663(0.006) | 0.649(0.018) | 0.629(0.011) | 0.682(0.001) | 0.646(0.003) | 0.312(0.012) |
| SVM | 0.581(0.004) | 0.473(0.009) | 0.571(0.012) | 0.612(0.088) | 0.533(0.102) | 0.544(0.018) | 0.607(0.008) | 0.572(0.029) | 0.144(0.015) |

| **Table S10:** Different machine learning models’ parameters of ROC curves in validation dateset of the predictive model for all- mortality at 120 months. | | | | | | | | | |
| --- | --- | --- | --- | --- | --- | --- | --- | --- | --- |
| **Models** | **AUC**  **(SD)** | **Cutoff**  **(SD)** | **Accuracy**  **(SD)** | **Sensitivity**  **(SD)** | **Specificity**  **(SD)** | **Positive Predictive Value (SD)** | **Passive Predictive Value Value(SD)** | **F1**  **(SD)** | **Kappa**  **(SD)** |
| XGBoost | 0.724(0.015) | 0.472(0.004) | 0.662(0.010) | 0.666(0.034) | 0.657(0.054) | 0.664(0.020) | 0.662(0.000) | 0.664(0.007) | 0.324(0.021) |
| logistic | 0.703(0.003) | 0.469(0.012) | 0.659(0.003) | 0.690(0.057) | 0.626(0.065) | 0.653(0.016) | 0.670(0.014) | 0.669(0.018) | 0.317(0.008) |
| LightGBM | 0.591(0.008) | 0.608(0.044) | 0.605(0.024) | 0.777(0.043) | 0.431(0.001) | 0.578(0.019) | 0.661(0.039) | 0.663(0.028) | 0.208(0.044) |
| RandomForest | 0.687(0.001) | 0.477(0.013) | 0.637(0.015) | 0.672(0.114) | 0.599(0.085) | 0.630(0.006) | 0.656(0.045) | 0.645(0.051) | 0.272(0.029) |
| AdaBoost | 0.717(0.007) | 0.497(0.000) | 0.649(0.010) | 0.686(0.040) | 0.610(0.062) | 0.641(0.018) | 0.660(0.002) | 0.662(0.009) | 0.296(0.021) |
| DecisionTree | 0.691(0.013) | 0.503(0.003) | 0.637(0.019) | 0.626(0.000) | 0.647(0.037) | 0.642(0.020) | 0.632(0.018) | 0.634(0.009) | 0.273(0.037) |
| MLP | 0.697(0.003) | 0.470(0.002) | 0.659(0.003) | 0.673(0.047) | 0.644(0.041) | 0.656(0.006) | 0.663(0.014) | 0.663(0.020) | 0.317(0.006) |
| SVM | 0.577(0.022) | 0.473(0.009) | 0.542(0.025) | 0.590(0.043) | 0.496(0.093) | 0.544(0.033) | 0.542(0.016) | 0.563(0.002) | 0.085(0.050) |

| **Table S11:** Different machine learning models’ parameters of ROC curves in training dateset of the predictive model for all- mortality at 150 months. | | | | | | | | | |
| --- | --- | --- | --- | --- | --- | --- | --- | --- | --- |
| **Models** | **AUC**  **(SD)** | **Cutoff**  **(SD)** | **Accuracy**  **(SD)** | **Sensitivity**  **(SD)** | **Specificity**  **(SD)** | **Positive Predictive Value (SD)** | **Passive Predictive Value Value(SD)** | **F1**  **(SD)** | **Kappa**  **(SD)** |
| XGBoost | 0.747(0.007) | 0.436(0.025) | 0.689(0.010) | 0.692(0.032) | 0.687(0.041) | 0.621(0.024) | 0.752(0.012) | 0.654(0.001) | 0.369(0.052) |
| logistic | 0.710(0.003) | 0.460(0.013) | 0.667(0.008) | 0.622(0.011) | 0.701(0.023) | 0.606(0.018) | 0.715(0.002) | 0.614(0.004) | 0.311(0.022) |
| LightGBM | 0.627(0.002) | 0.648(0.008) | 0.603(0.003) | 0.786(0.000) | 0.468(0.003) | 0.522(0.006) | 0.748(0.001) | 0.627(0.004) | 0.231(0.018) |
| RandomForest | 0.707(0.002) | 0.425(0.011) | 0.646(0.015) | 0.729(0.059) | 0.585(0.068) | 0.567(0.024) | 0.748(0.022) | 0.635(0.007) | 0.301(0.037) |
| AdaBoost | 0.741(0.007) | 0.495(0.000) | 0.683(0.005) | 0.719(0.012) | 0.656(0.001) | 0.607(0.007) | 0.760(0.004) | 0.658(0.009) | 0.373(0.036) |
| DecisionTree | 0.732(0.001) | 0.435(0.007) | 0.664(0.004) | 0.688(0.024) | 0.646(0.025) | 0.590(0.012) | 0.737(0.011) | 0.635(0.003) | 0.360(0.027) |
| MLP | 0.705(0.001) | 0.425(0.014) | 0.658(0.009) | 0.668(0.053) | 0.651(0.055) | 0.588(0.023) | 0.728(0.019) | 0.623(0.011) | 0.332(0.001) |
| SVM | 0.450(0.069) | 0.958(0.533) | 0.546(0.033) | 0.356(0.356) | 0.683(0.317) | NaN(NaN) | 0.603(0.024) | NaN(NaN) | -0.016(0.016) |

| **Table S12:** Different machine learning models’ parameters of ROC curves in validation dateset of the predictive model for all- mortality at 150 months. | | | | | | | | | |
| --- | --- | --- | --- | --- | --- | --- | --- | --- | --- |
| **Models** | **AUC**  **(SD)** | **Cutoff**  **(SD)** | **Accuracy**  **(SD)** | **Sensitivity**  **(SD)** | **Specificity**  **(SD)** | **Positive Predictive Value (SD)** | **Passive Predictive Value Value(SD)** | **F1**  **(SD)** | **Kappa**  **(SD)** |
| XGBoost | 0.737(0.018) | 0.436(0.025) | 0.687(0.023) | 0.675(0.064) | 0.694(0.011) | 0.653(0.028) | 0.717(0.025) | 0.663(0.045) | 0.369(0.052) |
| logistic | 0.709(0.009) | 0.460(0.013) | 0.659(0.009) | 0.598(0.011) | 0.712(0.011) | 0.639(0.027) | 0.674(0.005) | 0.618(0.018) | 0.311(0.022) |
| LightGBM | 0.619(0.007) | 0.648(0.008) | 0.606(0.013) | 0.781(0.002) | 0.457(0.013) | 0.552(0.022) | 0.709(0.005) | 0.646(0.016) | 0.231(0.018) |
| RandomForest | 0.699(0.017) | 0.425(0.011) | 0.649(0.017) | 0.719(0.074) | 0.586(0.036) | 0.597(0.019) | 0.715(0.030) | 0.652(0.042) | 0.301(0.037) |
| AdaBoost | 0.734(0.016) | 0.495(0.000) | 0.687(0.018) | 0.702(0.010) | 0.674(0.024) | 0.648(0.034) | 0.725(0.001) | 0.674(0.023) | 0.373(0.036) |
| DecisionTree | 0.697(0.007) | 0.435(0.007) | 0.680(0.012) | 0.712(0.049) | 0.651(0.022) | 0.635(0.016) | 0.728(0.015) | 0.671(0.031) | 0.360(0.027) |
| MLP | 0.711(0.011) | 0.425(0.014) | 0.668(0.003) | 0.656(0.045) | 0.677(0.043) | 0.636(0.000) | 0.698(0.001) | 0.645(0.022) | 0.332(0.001) |
| SVM | 0.436(0.018) | 0.958(0.533) | 0.495(0.028) | 0.320(0.320) | 0.664(0.336) | NaN(NaN) | 0.527(0.003) | NaN(NaN) | -0.016(0.016) |

| **Table S13:** Different machine learning models’ parameters of ROC curves in training dateset of the predictive model for cardiovascular mortality at 60 months. | | | | | | | | | |
| --- | --- | --- | --- | --- | --- | --- | --- | --- | --- |
| **Models** | **AUC**  **(SD)** | **Cutoff**  **(SD)** | **Accuracy**  **(SD)** | **Sensitivity**  **(SD)** | **Specificity**  **(SD)** | **Positive Predictive Value (SD)** | **Passive Predictive Value Value(SD)** | **F1**  **(SD)** | **Kappa**  **(SD)** |
| XGBoost | 0.732(0.002) | 0.197(0.015) | 0.618(0.038) | 0.769(0.065) | 0.578(0.066) | 0.322(0.012) | 0.909(0.013) | 0.452(0.000) | 0.230(0.016) |
| logistic | 0.663(0.009) | 0.172(0.012) | 0.531(0.046) | 0.824(0.046) | 0.455(0.071) | 0.282(0.011) | 0.911(0.008) | 0.419(0.006) | 0.164(0.024) |
| LightGBM | 0.780(0.005) | 0.205(0.003) | 0.668(0.027) | 0.784(0.073) | 0.637(0.053) | 0.359(0.008) | 0.922(0.018) | 0.491(0.007) | 0.293(0.005) |
| RandomForest | 0.652(0.003) | 0.209(0.003) | 0.568(0.061) | 0.700(0.070) | 0.534(0.095) | 0.283(0.018) | 0.874(0.004) | 0.400(0.006) | 0.153(0.030) |
| DecisionTree | 0.587(0.001) | 0.246(0.002) | 0.442(0.002) | 0.832(0.002) | 0.342(0.005) | 0.246(0.002) | 0.887(0.002) | 0.380(0.003) | 0.092(0.001) |
| AdaBoost | 0.723(0.007) | 0.474(0.001) | 0.561(0.031) | 0.865(0.028) | 0.482(0.047) | 0.302(0.009) | 0.933(0.006) | 0.447(0.006) | 0.205(0.019) |
| MLP | 0.598(0.007) | 0.429(0.045) | 0.484(0.124) | 0.756(0.208) | 0.413(0.210) | 0.255(0.014) | 0.900(0.055) | 0.373(0.012) | 0.101(0.022) |
| SVM | 0.516(0.087) | 0.160(0.048) | 0.326(0.123) | 0.903(0.097) | 0.179(0.177) | 0.225(0.022) | 0.937(0.063) | 0.358(0.021) | 0.044(0.044) |

| **Table S14:** Different machine learning models’ parameters of ROC curves in validation dateset of the predictive model for cardiovascular mortality at 60 months. | | | | | | | | | |
| --- | --- | --- | --- | --- | --- | --- | --- | --- | --- |
| **Models** | **AUC**  **(SD)** | **Cutoff**  **(SD)** | **Accuracy**  **(SD)** | **Sensitivity**  **(SD)** | **Specificity**  **(SD)** | **Positive Predictive Value (SD)** | **Passive Predictive Value Value(SD)** | **F1**  **(SD)** | **Kappa**  **(SD)** |
| XGBoost | 0.598(0.016) | 0.197(0.015) | 0.509(0.024) | 0.569(0.098) | 0.497(0.052) | 0.209(0.001) | 0.831(0.028) | 0.304(0.013) | 0.037(0.023) |
| logistic | 0.613(0.047) | 0.172(0.012) | 0.443(0.042) | 0.709(0.091) | 0.382(0.069) | 0.213(0.010) | 0.850(0.028) | 0.326(0.002) | 0.047(0.006) |
| LightGBM | 0.542(0.049) | 0.205(0.003) | 0.539(0.048) | 0.485(0.015) | 0.553(0.064) | 0.208(0.031) | 0.819(0.001) | 0.289(0.028) | 0.029(0.035) |
| RandomForest | 0.599(0.041) | 0.209(0.003) | 0.515(0.006) | 0.654(0.213) | 0.486(0.041) | 0.224(0.031) | 0.862(0.076) | 0.331(0.063) | 0.070(0.091) |
| DecisionTree | 0.563(0.005) | 0.246(0.002) | 0.410(0.009) | 0.812(0.012) | 0.314(0.021) | 0.219(0.010) | 0.876(0.009) | 0.345(0.014) | 0.062(0.002) |
| AdaBoost | 0.610(0.013) | 0.474(0.001) | 0.464(0.039) | 0.736(0.030) | 0.401(0.050) | 0.226(0.021) | 0.865(0.008) | 0.345(0.021) | 0.075(0.018) |
| MLP | 0.539(0.050) | 0.429(0.045) | 0.443(0.102) | 0.641(0.259) | 0.403(0.184) | 0.196(0.005) | 0.848(0.061) | 0.292(0.037) | 0.014(0.037) |
| SVM | 0.474(0.068) | 0.160(0.048) | 0.269(0.060) | 0.867(0.133) | 0.124(0.117) | 0.190(0.015) | 0.902(0.098) | 0.311(0.029) | -0.004(0.007) |

| **Table S15:** Different machine learning models’ parameters of ROC curves in training dateset of the predictive model for cardiovascular mortality at 120 months. | | | | | | | | | |
| --- | --- | --- | --- | --- | --- | --- | --- | --- | --- |
| **Models** | **AUC**  **(SD)** | **Cutoff**  **(SD)** | **Accuracy**  **(SD)** | **Sensitivity**  **(SD)** | **Specificity**  **(SD)** | **Positive Predictive Value (SD)** | **Passive Predictive Value Value(SD)** | **F1**  **(SD)** | **Kappa**  **(SD)** |
| XGBoost | 0.693(0.003) | 0.179(0.012) | 0.558(0.021) | 0.798(0.035) | 0.498(0.034) | 0.283(0.007) | 0.909(0.009) | 0.417(0.002) | 0.176(0.007) |
| logistic | 0.643(0.004) | 0.169(0.005) | 0.509(0.011) | 0.778(0.011) | 0.443(0.017) | 0.257(0.004) | 0.890(0.002) | 0.387(0.004) | 0.125(0.006) |
| LightGBM | 0.565(0.005) | 0.203(0.009) | 0.444(0.001) | 0.810(0.016) | 0.353(0.006) | 0.237(0.003) | 0.883(0.006) | 0.366(0.006) | 0.085(0.005) |
| RandomForest | 0.635(0.006) | 0.214(0.004) | 0.538(0.004) | 0.710(0.019) | 0.495(0.010) | 0.258(0.003) | 0.873(0.004) | 0.379(0.006) | 0.124(0.004) |
| AdaBoost | 0.666(0.002) | 0.434(0.000) | 0.588(0.007) | 0.689(0.015) | 0.563(0.012) | 0.281(0.000) | 0.879(0.002) | 0.399(0.003) | 0.162(0.000) |
| DecisionTree | 0.580(0.007) | 0.236(0.004) | 0.441(0.002) | 0.810(0.016) | 0.350(0.003) | 0.236(0.004) | 0.882(0.007) | 0.365(0.007) | 0.083(0.007) |
| MLP | 0.601(0.017) | 0.382(0.045) | 0.568(0.022) | 0.620(0.054) | 0.555(0.040) | 0.257(0.001) | 0.856(0.008) | 0.362(0.010) | 0.114(0.003) |
| SVM | 0.509(0.052) | 0.210(0.010) | 0.623(0.087) | 0.395(0.211) | 0.679(0.161) | 0.230(0.009) | 0.824(0.017) | 0.272(0.071) | 0.052(0.027) |

| **Table S16:** Different machine learning models’ parameters of ROC curves in validation dateset of the predictive model for cardiovascular mortality at 120 months. | | | | | | | | | |
| --- | --- | --- | --- | --- | --- | --- | --- | --- | --- |
| **Models** | **AUC**  **(SD)** | **Cutoff**  **(SD)** | **Accuracy**  **(SD)** | **Sensitivity**  **(SD)** | **Specificity**  **(SD)** | **Positive Predictive Value (SD)** | **Passive Predictive Value Value(SD)** | **F1**  **(SD)** | **Kappa**  **(SD)** |
| XGBoost | 0.604(0.003) | 0.179(0.012) | 0.532(0.029) | 0.637(0.100) | 0.507(0.059) | 0.229(0.012) | 0.861(0.016) | 0.336(0.028) | 0.085(0.019) |
| logistic | 0.584(0.013) | 0.169(0.005) | 0.486(0.027) | 0.664(0.090) | 0.445(0.055) | 0.216(0.012) | 0.855(0.014) | 0.325(0.024) | 0.060(0.016) |
| LightGBM | 0.566(0.025) | 0.203(0.009) | 0.443(0.007) | 0.809(0.068) | 0.358(0.010) | 0.225(0.018) | 0.893(0.029) | 0.352(0.028) | 0.083(0.029) |
| RandomForest | 0.600(0.029) | 0.214(0.004) | 0.534(0.007) | 0.666(0.036) | 0.503(0.001) | 0.236(0.015) | 0.868(0.008) | 0.349(0.022) | 0.100(0.022) |
| AdaBoost | 0.595(0.011) | 0.434(0.000) | 0.551(0.017) | 0.557(0.075) | 0.549(0.038) | 0.221(0.014) | 0.844(0.009) | 0.316(0.027) | 0.066(0.021) |
| DecisionTree | 0.583(0.029) | 0.236(0.004) | 0.443(0.007) | 0.809(0.068) | 0.358(0.010) | 0.225(0.018) | 0.893(0.029) | 0.352(0.028) | 0.083(0.029) |
| MLP | 0.578(0.055) | 0.382(0.045) | 0.541(0.041) | 0.556(0.093) | 0.537(0.028) | 0.218(0.044) | 0.840(0.031) | 0.313(0.061) | 0.060(0.077) |
| SVM | 0.512(0.006) | 0.210(0.010) | 0.610(0.100) | 0.401(0.173) | 0.660(0.164) | 0.219(0.017) | 0.828(0.011) | 0.266(0.034) | 0.047(0.006) |

| **Table S17:** Different machine learning models’ parameters of ROC curves in training dateset of the predictive model for cardiovascular mortality at 150 months. | | | | | | | | | |
| --- | --- | --- | --- | --- | --- | --- | --- | --- | --- |
| **Models** | **AUC**  **(SD)** | **Cutoff**  **(SD)** | **Accuracy**  **(SD)** | **Sensitivity**  **(SD)** | **Specificity**  **(SD)** | **Positive Predictive Value (SD)** | **Passive Predictive Value Value(SD)** | **F1**  **(SD)** | **Kappa**  **(SD)** |
| XGBoost | 0.693(0.007) | 0.189(0.017) | 0.624(0.031) | 0.648(0.061) | 0.620(0.051) | 0.275(0.014) | 0.889(0.012) | 0.385(0.003) | 0.175(0.009) |
| logistic | 0.640(0.006) | 0.155(0.008) | 0.501(0.030) | 0.791(0.037) | 0.436(0.048) | 0.238(0.001) | 0.905(0.003) | 0.365(0.003) | 0.119(0.009) |
| LightGBM | 0.563(0.028) | 0.272(0.001) | 0.450(0.004) | 0.760(0.056) | 0.382(0.015) | 0.214(0.003) | 0.878(0.024) | 0.333(0.009) | 0.070(0.017) |
| RandomForest | 0.640(0.002) | 0.194(0.004) | 0.530(0.045) | 0.711(0.076) | 0.490(0.071) | 0.237(0.011) | 0.886(0.016) | 0.354(0.003) | 0.113(0.009) |
| AdaBoost | 0.662(0.000) | 0.428(0.003) | 0.579(0.032) | 0.694(0.052) | 0.554(0.049) | 0.257(0.013) | 0.891(0.011) | 0.374(0.006) | 0.149(0.011) |
| MLP | 0.519(0.047) | 0.280(0.080) | 0.445(0.099) | 0.670(0.088) | 0.394(0.142) | 0.200(0.012) | 0.838(0.018) | 0.306(0.005) | 0.037(0.033) |
| SVM | 0.436(0.001) | 0.206(0.007) | 0.789(0.022) | 0.061(0.028) | 0.951(0.028) | 0.230(0.020) | 0.820(0.005) | 0.092(0.034) | 0.017(0.001) |
| DecisionTree | 0.703(0.004) | 0.189(0.009) | 0.611(0.013) | 0.702(0.030) | 0.590(0.023) | 0.275(0.003) | 0.900(0.003) | 0.396(0.008) | 0.182(0.001) |

| **Table S18:** Different machine learning models’ parameters of ROC curves in validation dateset of the predictive model for cardiovascular mortality at 150 months. | | | | | | | | | |
| --- | --- | --- | --- | --- | --- | --- | --- | --- | --- |
| **Models** | **AUC**  **(SD)** | **Cutoff**  **(SD)** | **Accuracy**  **(SD)** | **Sensitivity**  **(SD)** | **Specificity**  **(SD)** | **Positive Predictive Value (SD)** | **Passive Predictive Value Value(SD)** | **F1**  **(SD)** | **Kappa**  **(SD)** |
| XGBoost | 0.660(0.025) | 0.189(0.017) | 0.603(0.046) | 0.588(0.017) | 0.605(0.062) | 0.275(0.002) | 0.852(0.022) | 0.374(0.001) | 0.136(0.033) |
| logistic | 0.654(0.024) | 0.155(0.008) | 0.513(0.032) | 0.747(0.063) | 0.457(0.049) | 0.258(0.024) | 0.878(0.028) | 0.381(0.018) | 0.118(0.007) |
| LightGBM | 0.602(0.011) | 0.272(0.001) | 0.481(0.009) | 0.814(0.011) | 0.397(0.018) | 0.254(0.014) | 0.893(0.021) | 0.386(0.015) | 0.115(0.009) |
| RandomForest | 0.625(0.019) | 0.194(0.004) | 0.541(0.022) | 0.671(0.052) | 0.505(0.044) | 0.255(0.020) | 0.861(0.005) | 0.370(0.029) | 0.110(0.007) |
| AdaBoost | 0.638(0.014) | 0.428(0.003) | 0.577(0.020) | 0.632(0.013) | 0.562(0.030) | 0.267(0.013) | 0.858(0.017) | 0.375(0.015) | 0.128(0.008) |
| MLP | 0.517(0.038) | 0.280(0.080) | 0.438(0.093) | 0.633(0.081) | 0.393(0.131) | 0.212(0.035) | 0.805(0.001) | 0.313(0.028) | 0.021(0.032) |
| SVM | 0.428(0.034) | 0.206(0.007) | 0.762(0.003) | 0.032(0.032) | 0.948(0.033) | 0.071(0.071) | 0.795(0.019) | NaN(NaN) | -0.028(0.000) |
| DecisionTree | 0.540(0.019) | 0.189(0.009) | 0.530(0.003) | 0.517(0.009) | 0.533(0.006) | 0.219(0.021) | 0.814(0.016) | 0.307(0.022) | 0.034(0.004) |

**Table S19 - Cox proportional hazards analysis of SIRI for all-cause and cardiovascular mortality in the cohort excluding patients died in 2 years under fully-adjusted model.**

| **Subgroup** | **Quartile** | | | | **P for trend** |
| --- | --- | --- | --- | --- | --- |
|  | Q1 | Q2 | Q3 | Q4 |  |
| **All-cause mortality** | | | | | |
| **SIRI** | Reference | 1.18 (0.92 ~ 1.50) | 1.52 (1.20 ~ 1.92) | 2.06 (1.62 ~ 2.62) | <.001 |
| **Cardiovascular mortality** | | | | | |
| **SIRI** | Reference | 1.51 (1.05 ~ 2.18) | 1.37 (0.93 ~ 2.00) | 1.70 (1.14 ~ 2.51) | <.001 |
| Adjusted for gender, age, ethnicity, education, and poverty income ratio, systolic blood pressure, diastolic blood pressure, eGFR, BMI, uric acid, glycated hemoglobin (HbA1c), total cholesterol, HDL-C, smoking, drinking, hypertension, diabetes, liver disease, cancer, and CVD.  SIRI, Systemic Inflammation Response Index | | | | | |

**Table S20 - Cox proportional hazards analysis of SIRI for all-cause and cardiovascular mortality in the cohort of NHANES 1999-2000 under fully-adjusted model.**

| **Subgroup** | **Quartile** | | | | **P for trend** |
| --- | --- | --- | --- | --- | --- |
|  | Q1 | Q2 | Q3 | Q4 |  |
| **All-cause mortality** | | | | | |
| **SIRI** | Reference | 2.01 (1.13 ~ 3.59) | 1.49 (0.79 ~ 2.82) | 2.12 (1.14 ~ 3.95) | <.001 |
| **Cardiovascular mortality** | | | | | |
| **SIRI** | Reference | 1.21 (0.51 ~ 2.86) | 1.13 (0.43 ~ 2.93) | 1.30 (0.51 ~ 3.30) | 0.625 |
| Adjusted for gender, age, ethnicity, education, and poverty income ratio, systolic blood pressure, diastolic blood pressure, eGFR, BMI, uric acid, glycated hemoglobin (HbA1c), total cholesterol, HDL-C, smoking, drinking, hypertension, diabetes, liver disease, cancer, and CVD.  SIRI, Systemic Inflammation Response Index | | | | | |

| **Table S21 -** Baseline characteristics of Gaoyou cohort. | | | | |
| --- | --- | --- | --- | --- |
| Variables | Total (n = 364) | 0 (n = 348) | 1 (n = 16) | *P* |
|  |  |  |  |  |
| SIRI | 0.68 ± 0.61 | 0.67 ± 0.61 | 0.88 ± 0.62 | **0.043** |
| SBP | 152.06 ± 19.57 | 151.88 ± 19.29 | 156.08 ± 25.34 | 0.522 |
| DBP | 91.58 ± 9.60 | 91.72 ± 9.61 | 88.54 ± 9.25 | 0.196 |
| UA | 5.51 ± 1.67 | 5.49 ± 1.66 | 5.76 ± 2.06 | 0.531 |
| HbA1c | 7.00 ± 1.73 | 6.97 ± 1.68 | 7.76 ± 2.50 | 0.074 |
| HDL-c | 48.56 ± 11.58 | 48.52 ± 11.70 | 49.47 ± 8.77 | 0.748 |
| TC | 213.12 ± 46.29 | 211.93 ± 45.64 | 239.03 ± 53.88 | **0.022** |
| Gender, n(%) |  |  |  | 0.946 |
| Male | 179 (49.18) | 171 (49.14) | 8 (50.00) |  |
| Female | 185 (50.82) | 177 (50.86) | 8 (50.00) |  |
| Age, n(%) |  |  |  | **<.001** |
| < 60 | 249 (68.41) | 245 (70.40) | 4 (25.00) |  |
| ≥ 60 | 115 (31.59) | 103 (29.60) | 12 (75.00) |  |
| Education, n(%) |  |  |  | 1.000 |
| Below high school | 323 (88.74) | 308 (88.51) | 15 (93.75) |  |
| High school | 29 (7.97) | 28 (8.05) | 1 (6.25) |  |
| Above high school | 12 (3.30) | 12 (3.45) | 0 (0.00) |  |
| Income, n(%) |  |  |  | 0.081 |
| ＜200 | 20 (5.49) | 17 (4.89) | 3 (18.75) |  |
| 200－500 | 40 (10.99) | 38 (10.92) | 2 (12.50) |  |
| 500－1500 | 167 (45.88) | 158 (45.40) | 9 (56.25) |  |
| 1500－300 | 78 (21.43) | 77 (22.13) | 1 (6.25) |  |
| ＞3000 | 59 (16.21) | 58 (16.67) | 1 (6.25) |  |
| BMI, n(%) |  |  |  | 0.082 |
| < 28 | 260 (71.43) | 245 (70.40) | 15 (93.75) |  |
| ≥ 28 | 104 (28.57) | 103 (29.60) | 1 (6.25) |  |
| Hypertension, n(%) |  |  |  | **0.004** |
| No | 17 (4.67) | 13 (3.74) | 4 (25.00) |  |
| Yes | 347 (95.33) | 335 (96.26) | 12 (75.00) |  |
| EGFR, n(%) |  |  |  | 0.259 |
| < 60 | 281 (77.20) | 271 (77.87) | 10 (62.50) |  |
| ≥ 60 | 83 (22.80) | 77 (22.13) | 6 (37.50) |  |
| Diabetes, n(%) |  |  |  | 0.374 |
| No | 293 (80.49) | 282 (81.03) | 11 (68.75) |  |
| Yes | 71 (19.51) | 66 (18.97) | 5 (31.25) |  |
| Smoking, n(%) |  |  |  | 0.532 |
| No | 247 (67.86) | 235 (67.53) | 12 (75.00) |  |
| Yes | 117 (32.14) | 113 (32.47) | 4 (25.00) |  |
| Drinking, n(%) |  |  |  | 0.247 |
| No | 288 (79.12) | 273 (78.45) | 15 (93.75) |  |
| Yes | 76 (20.88) | 75 (21.55) | 1 (6.25) |  |
| CVD, n(%) |  |  |  | **0.034** |
| No | 322 (88.46) | 311 (89.37) | 11 (68.75) |  |
| Yes | 42 (11.54) | 37 (10.63) | 5 (31.25) |  |
| Stroke, n(%) |  |  |  | **0.044** |
| No | 345 (94.78) | 332 (95.40) | 13 (81.25) |  |
| Yes | 19 (5.22) | 16 (4.60) | 3 (18.75) |  |
| Note: BMI, Body Mass Index; SBP, Systolic Blood Pressure; DBP, Diastolic Blood Pressure; UA, Uric Acid; HbA1c, Hemoglobin A1c; HDL-C, High-Density Lipoprotein Cholesterol; TC, Total Cholesterol; PIR, Poverty Income Ratio; eGFR, Estimated Glomerular Filtration Rate; SIRI, Systemic Inflammation Response Index; | | | | |

**Table S22 -** Cox proportional hazards analysis of SIRI for all-cause and cardiovascular mortality in the Gaoyou cohort under fully-adjusted model.

| **Subgroup** | **Quartile** | | | | **P for trend** |
| --- | --- | --- | --- | --- | --- |
|  | Q1 | Q2 | Q3 | Q4 |  |
| **All-cause mortality** | | | | | |
| **SIRI** | Reference | 1.48 (0.25 ~ 8.87) | 2.57 (0.50 ~ 13.24) | 3.07 (0.62 ~ 15.22) | <.001 |
| **Cardiovascular mortality** | | | | | |
| **SIRI** | Reference | 1.97 (0.36 ~ 10.78) | 3.08 (0.62 ~ 15.27) | 4.09 (0.87 ~ 19.25) | 0.033 |
| Adjusted for gender, age, education, systolic blood pressure, diastolic blood pressure, eGFR, BMI, uric acid, glycated hemoglobin (HbA1c), total cholesterol, HDL-C, smoking, drinking, hypertension, diabetes, liver disease, cancer, and CVD.  SIRI, Systemic Inflammation Response Index | | | | | |

| **Table S23:** ROCs and C-index of the prognostic model in the NHANES 2001-2018. | | | | |
| --- | --- | --- | --- | --- |
| SIRI | ROC-60 | ROC-120 | ROC-150 | C-index |
| All-cause mortality | 0.723 | 0.726 | 0.753 | 0.6922 |
| Cardiovascular mortality | 0.721 | 0.713 | 0.752 | 0.6911 |
| NLR | ROC-60 | ROC-120 | ROC-150 | C-index |
| All-cause mortality | 0.719 | 0.719 | 0.748 | 0.6899 |
| Cardiovascular mortality | 0.716 | 0.707 | 0.743 | 0.6901 |
| PLR | ROC-60 | ROC-120 | ROC-150 | C-index |
| All-cause mortality | 0.698 | 0.697 | 0.722 | 0.6728 |
| Cardiovascular mortality | 0.711 | 0.692 | 0.731 | 0.6855 |
| PNR | ROC-60 | ROC-120 | ROC-150 | C-index |
| All-cause mortality | 0.710 | 0.713 | 0.733 | 0.6827 |
| Cardiovascular mortality | 0.712 | 0.712 | 0.741 | 0.6831 |
| SII | ROC-60 | ROC-120 | ROC-150 | C-index |
| All-cause mortality | 0.700 | 0.701 | 0.732 | 0.6750 |
| Cardiovascular mortality | 0.706 | 0.696 | 0.736 | 0.6809 |
| NHR | ROC-60 | ROC-120 | ROC-150 | C-index |
| All-cause mortality | 0.694 | 0.708 | 0.738 | 0.6727 |
| Cardiovascular mortality | 0.702 | 0.701 | 0.742 | 0.6788 |
| MHR | ROC-60 | ROC-120 | ROC-150 | C-index |
| All-cause mortality | 0.692 | 0.705 | 0.735 | 0.6688 |
| Cardiovascular mortality | 0.703 | 0.705 | 0.744 | 0.6796 |
| PHR | ROC-60 | ROC-120 | ROC-150 | C-index |
| All-cause mortality | 0.694 | 0.696 | 0.732 | 0.6679 |
| Cardiovascular mortality | 0.705 | 0.695 | 0.735 | 0.6791 |
| LHR | ROC-60 | ROC-120 | ROC-150 | C-index |
| All-cause mortality | 0.693 | 0.695 | 0.728 | 0.6676 |
| Cardiovascular mortality | 0.705 | 0.695 | 0.735 | 0.6788 |
| NMR | ROC-60 | ROC-120 | ROC-150 | C-index |
| All-cause mortality | 0.692 | 0.697 | 0.730 | 0.6683 |
| Cardiovascular mortality | 0.706 | 0.694 | 0.734 | 0.6798 |
| Note: NLR, Neutrophil-to-Lymphocyte Ratio; PLR, Platelet-to-Lymphocyte Ratio; PNR, Platelet-to-Neutrophil Ratio; SII, Systemic Immune-Inflammation Index; SIRI, Systemic Inflammation Response Index; NHR, Neutrophil-to-High-Density Lipoprotein Ratio; MHR, Monocyte-to-High-Density Lipoprotein Ratio; PHR, Platelet-to-High-Density Lipoprotein Ratio; LHR, Lymphocyte-to-High-Density Lipoprotein Ratio; NMR, Neutrophil-to-Monocyte Ratio. | | | | |

| **Table S24:** ROCs and C-index of the prognostic model in the NHANES 1999-2000. | | | | |
| --- | --- | --- | --- | --- |
| SIRI | ROC-60 | ROC-120 | ROC-150 | C-index |
| All-cause mortality | 0.765 | 0.752 | 0.775 | 0.6942 |
| Cardiovascular mortality | 0.708 | 0.734 | 0.718 | 0.6626 |
| NLR | ROC-60 | ROC-120 | ROC-150 | C-index |
| All-cause mortality | 0.764 | 0.744 | 0.767 | 0.6914 |
| Cardiovascular mortality | 0.704 | 0.728 | 0.711 | 0.6613 |
| PLR | ROC-60 | ROC-120 | ROC-150 | C-index |
| All-cause mortality | 0.742 | 0.730 | 0.763 | 0.6827 |
| Cardiovascular mortality | 0.700 | 0.728 | 0.710 | 0.6603 |
| PNR | ROC-60 | ROC-120 | ROC-150 | C-index |
| All-cause mortality | 0.739 | 0.729 | 0.764 | 0.6821 |
| Cardiovascular mortality | 0.702 | 0.728 | 0.712 | 0.6620 |
| SII | ROC-60 | ROC-120 | ROC-150 | C-index |
| All-cause mortality | 0.752 | 0.744 | 0.768 | 0.6890 |
| Cardiovascular mortality | 0.705 | 0.729 | 0.709 | 0.6609 |
| NHR | ROC-60 | ROC-120 | ROC-150 | C-index |
| All-cause mortality | 0.758 | 0.747 | 0.770 | 0.6901 |
| Cardiovascular mortality | 0.701 | 0.730 | 0.719 | 0.6611 |
| MHR | ROC-60 | ROC-120 | ROC-150 | C-index |
| All-cause mortality | 0.734 | 0.731 | 0.769 | 0.6809 |
| Cardiovascular mortality | 0.698 | 0.725 | 0.703 | 0.6615 |
| PHR | ROC-60 | ROC-120 | ROC-150 | C-index |
| All-cause mortality | 0.726 | 0.731 | 0.771 | 0.6836 |
| Cardiovascular mortality | 0.665 | 0.709 | 0.733 | 0.6720 |
| LHR | ROC-60 | ROC-120 | ROC-150 | C-index |
| All-cause mortality | 0.726 | 0.726 | 0.768 | 0.6790 |
| Cardiovascular mortality | 0.691 | 0.719 | 0.715 | 0.6616 |
| NMR | ROC-60 | ROC-120 | ROC-150 | C-index |
| All-cause mortality | 0.730 | 0.727 | 0.756 | 0.6808 |
| Cardiovascular mortality | 0.699 | 0.728 | 0.710 | 0.6601 |
| Note: NLR, Neutrophil-to-Lymphocyte Ratio; PLR, Platelet-to-Lymphocyte Ratio; PNR, Platelet-to-Neutrophil Ratio; SII, Systemic Immune-Inflammation Index; SIRI, Systemic Inflammation Response Index; NHR, Neutrophil-to-High-Density Lipoprotein Ratio; MHR, Monocyte-to-High-Density Lipoprotein Ratio; PHR, Platelet-to-High-Density Lipoprotein Ratio; LHR, Lymphocyte-to-High-Density Lipoprotein Ratio; NMR, Neutrophil-to-Monocyte Ratio. | | | | |

| **Table S25:** ROCs and C-index of the prognostic model in the Gaoyou cohort. | | |
| --- | --- | --- |
| SIRI | ROC-50 | C-index |
| All-cause mortality | 0.805 | 0.8085 |
| Cardiovascular mortality | 0.862 | 0.8603 |
| NLR | ROC-50 | C-index |
| All-cause mortality | 0.800 | 0.8031 |
| Cardiovascular mortality | 0.851 | 0.8560 |
| PLR | ROC-50 | C-index |
| All-cause mortality | 0.800 | 0.8020 |
| Cardiovascular mortality | 0.853 | 0.8557 |
| PNR | ROC-50 | C-index |
| All-cause mortality | 0.781 | 0.7869 |
| Cardiovascular mortality | 0.854 | 0.8562 |
| SII | ROC-50 | C-index |
| All-cause mortality | 0.803 | 0.8059 |
| Cardiovascular mortality | 0.835 | 0.8372 |
| NHR | ROC-50 | C-index |
| All-cause mortality | 0.802 | 0.8026 |
| Cardiovascular mortality | 0.846 | 0.8473 |
| MHR | ROC-50 | C-index |
| All-cause mortality | 0.801 | 0.8027 |
| Cardiovascular mortality | 0.837 | 0.8390 |
| PHR | ROC-50 | C-index |
| All-cause mortality | 0.803 | 0.8051 |
| Cardiovascular mortality | 0.843 | 0.8466 |
| LHR | ROC-50 | C-index |
| All-cause mortality | 0.803 | 0.8059 |
| Cardiovascular mortality | 0.851 | 0.8548 |
| NMR | ROC-50 | C-index |
| All-cause mortality | 0.803 | 0.8035 |
| Cardiovascular mortality | 0.850 | 0.8547 |
| Note: NLR, Neutrophil-to-Lymphocyte Ratio; PLR, Platelet-to-Lymphocyte Ratio; PNR, Platelet-to-Neutrophil Ratio; SII, Systemic Immune-Inflammation Index; SIRI, Systemic Inflammation Response Index; NHR, Neutrophil-to-High-Density Lipoprotein Ratio; MHR, Monocyte-to-High-Density Lipoprotein Ratio; PHR, Platelet-to-High-Density Lipoprotein Ratio; LHR, Lymphocyte-to-High-Density Lipoprotein Ratio; NMR, Neutrophil-to-Monocyte Ratio. | | |

| A.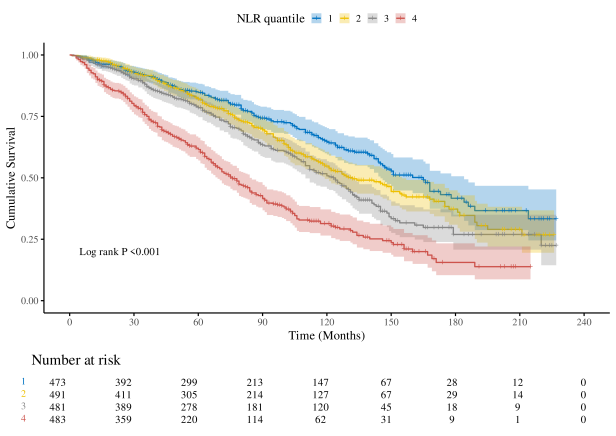 | B.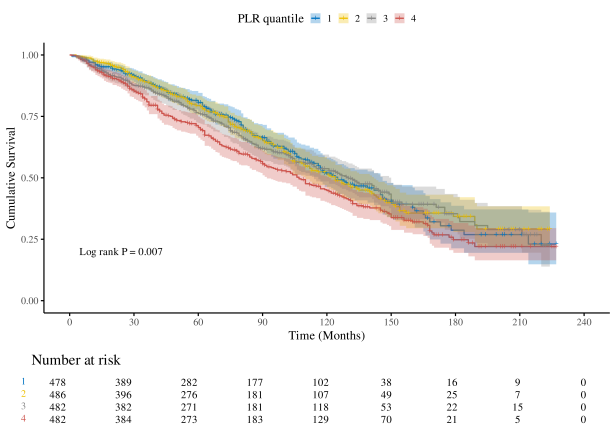 |
| --- | --- |
| C.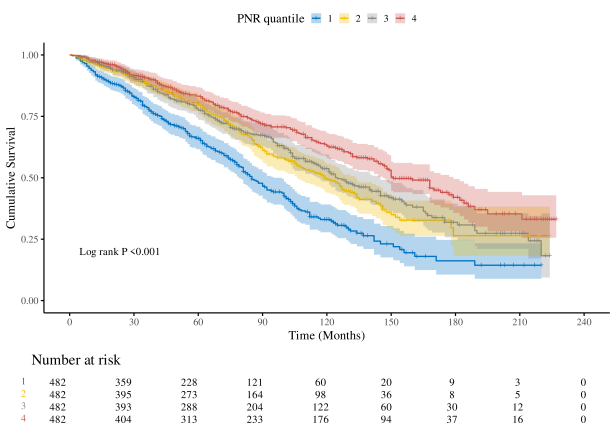 | D.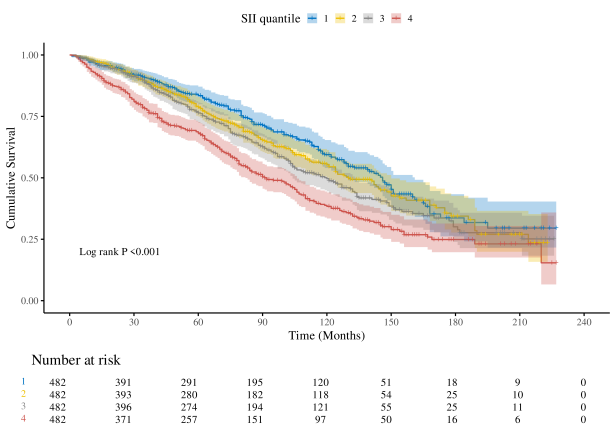 |
| E.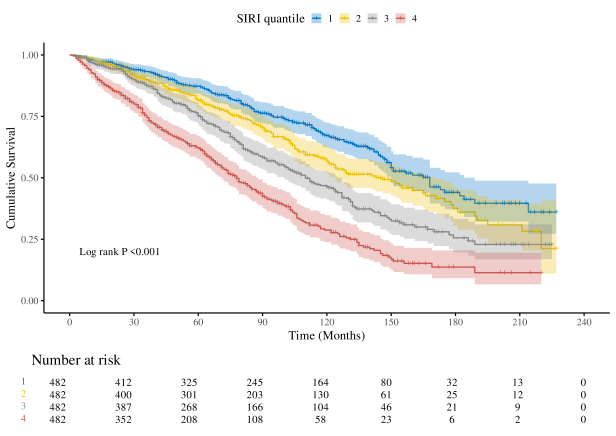 | F.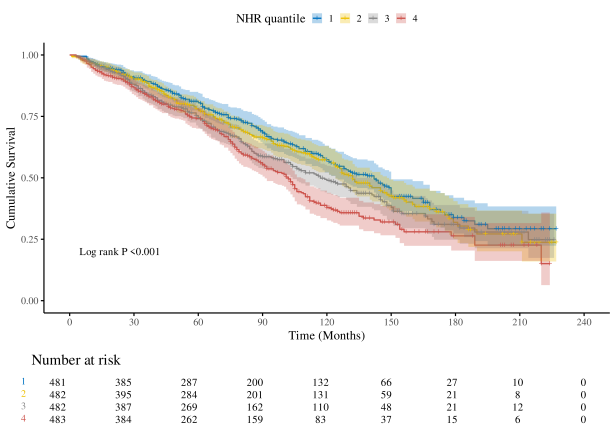 |
| G.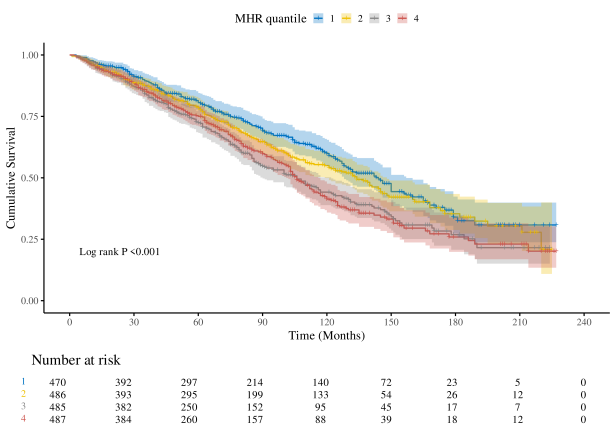 | H.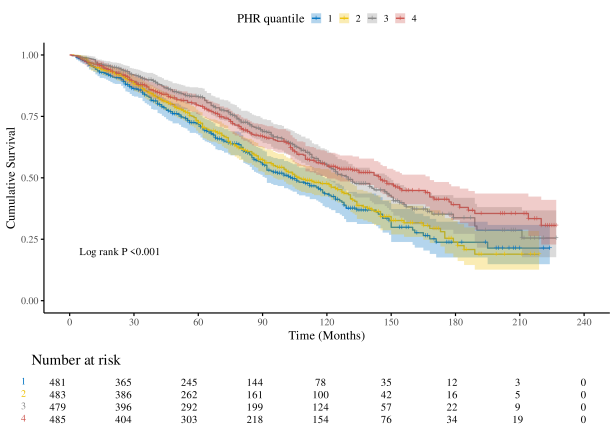 |
| I.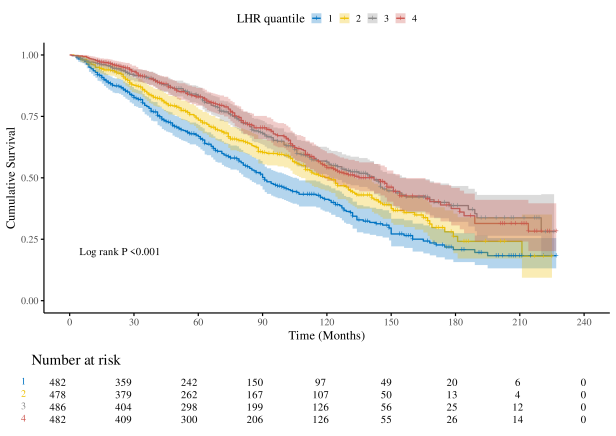 | J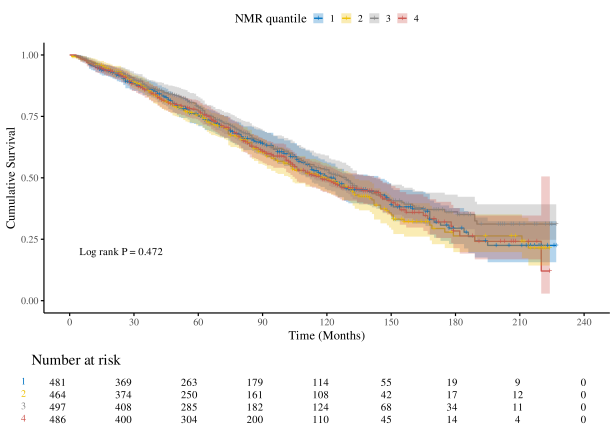 |
| **Figure S1 - Kaplan-Meier survival curves stratified by quartiles of inflammatory and nutritional parameters for all-cause mortality.**  (A) NLR, Neutrophil-to-Lymphocyte Ratio; (B) PLR, Platelet-to-Lymphocyte Ratio; (C) PNR, Platelet-to-Neutrophil Ratio; (D) SII, Systemic Immune-Inflammation Index; (E) SIRI, Systemic Inflammation Response Index; (F) NHR, Neutrophil-to-High-Density Lipoprotein Ratio; (G) MHR, Monocyte-to-High-Density Lipoprotein Ratio; (H) PHR, Platelet-to-High-Density Lipoprotein Ratio; (I) LHR, Lymphocyte-to-High-Density Lipoprotein Ratio; (J) NMR, Neutrophil-to-Monocyte Ratio. | |

| A.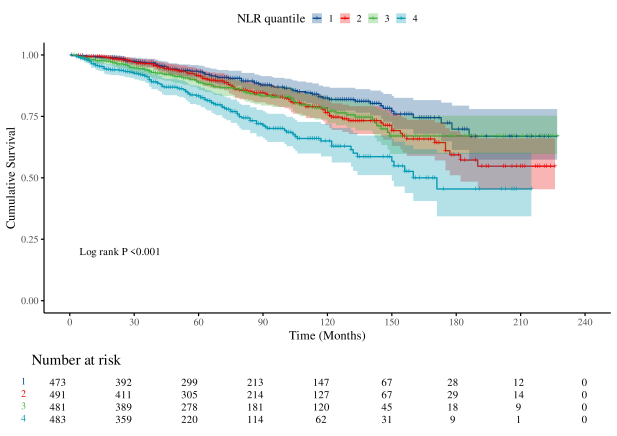 | B.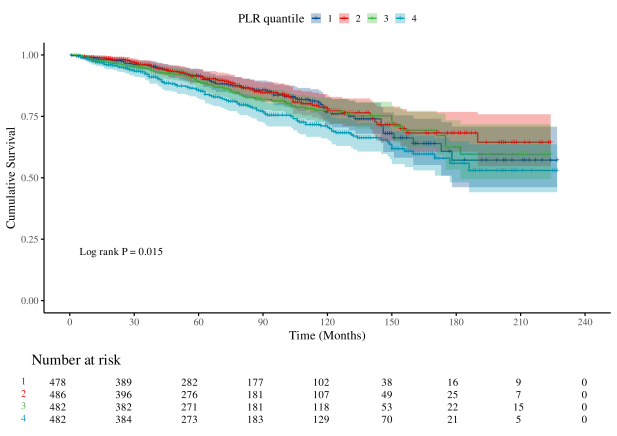 |
| --- | --- |
| C.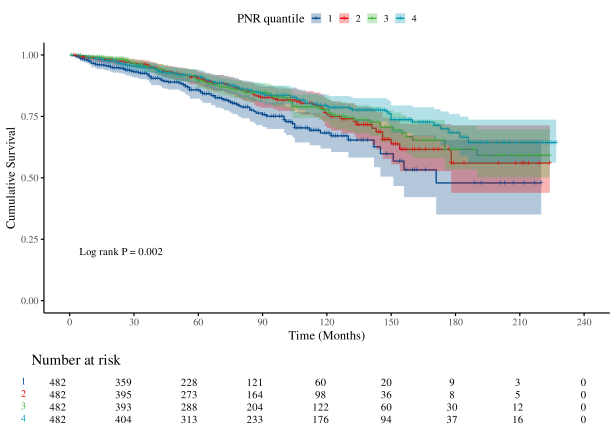 | D.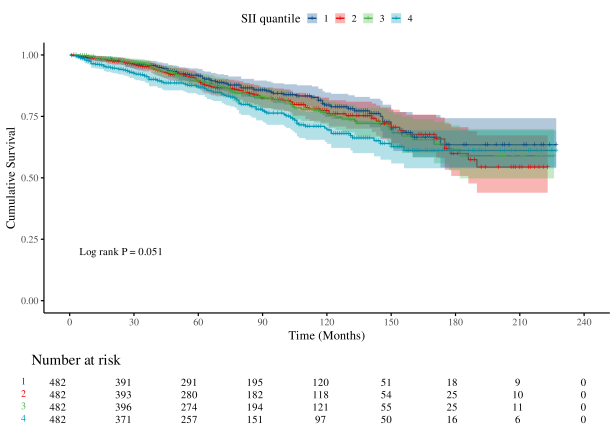 |
| E.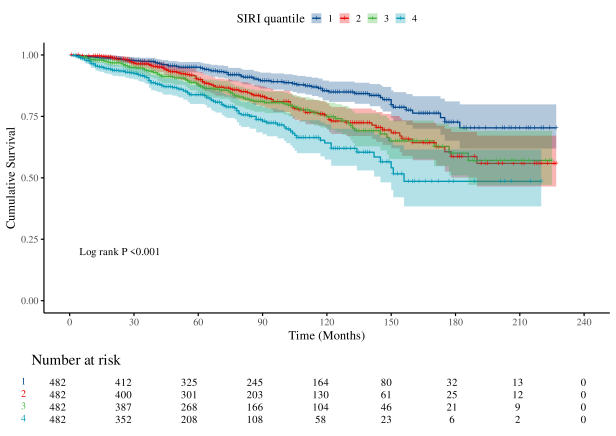 | F.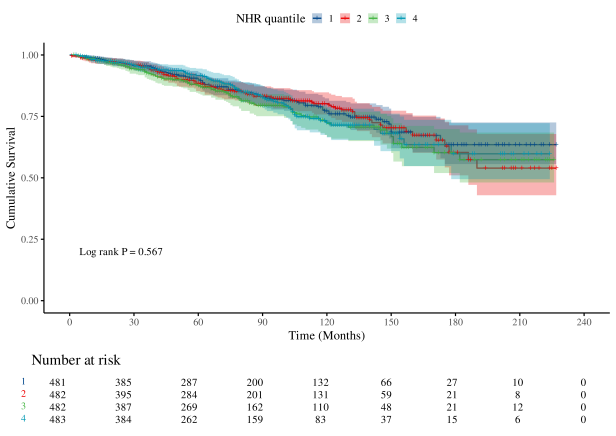 |
| G.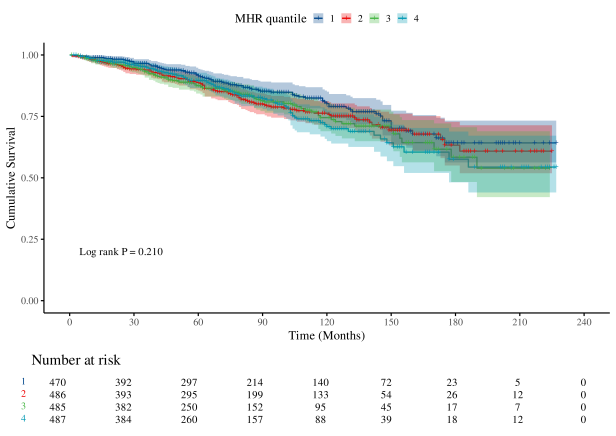 | H.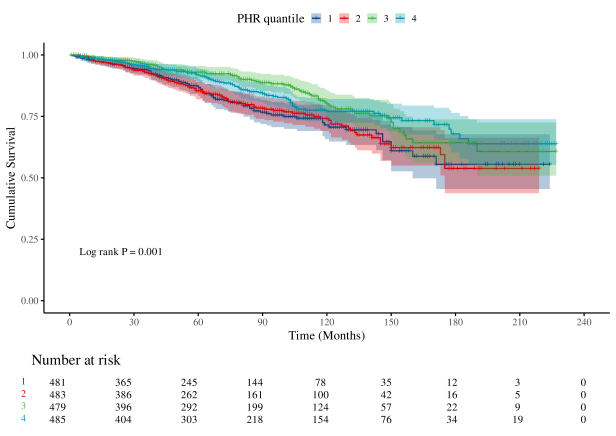 |
| I.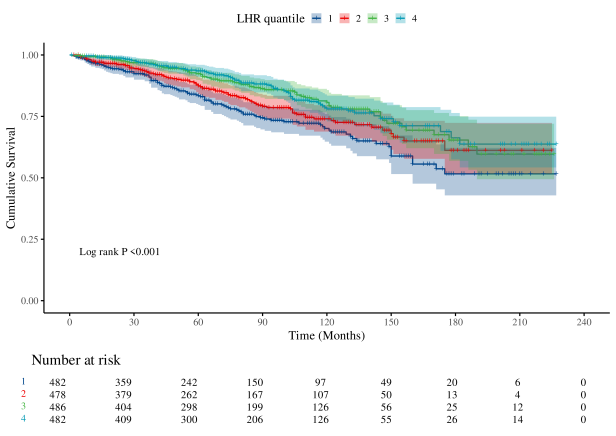 | J.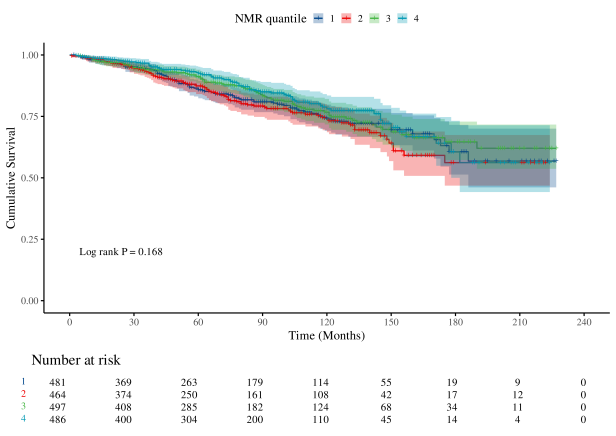 |
| **Figure S2 - Kaplan-Meier survival curves stratified by quartiles of inflammatory and nutritional parameters for cardiovascular mortality.**  (A) NLR, Neutrophil-to-Lymphocyte Ratio; (B) PLR, Platelet-to-Lymphocyte Ratio; (C) PNR, Platelet-to-Neutrophil Ratio; (D) SII, Systemic Immune-Inflammation Index; (E) SIRI, Systemic Inflammation Response Index; (F) NHR, Neutrophil-to-High-Density Lipoprotein Ratio; (G) MHR, Monocyte-to-High-Density Lipoprotein Ratio; (H) PHR, Platelet-to-High-Density Lipoprotein Ratio; (I) LHR, Lymphocyte-to-High-Density Lipoprotein Ratio; (J) NMR, Neutrophil-to-Monocyte Ratio. | |

| 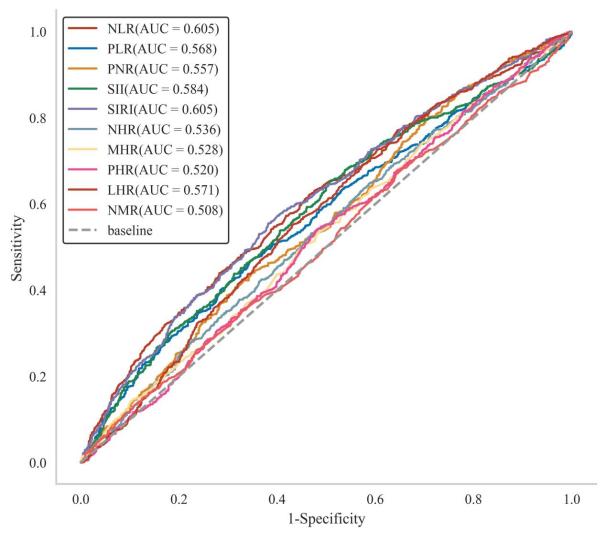 | 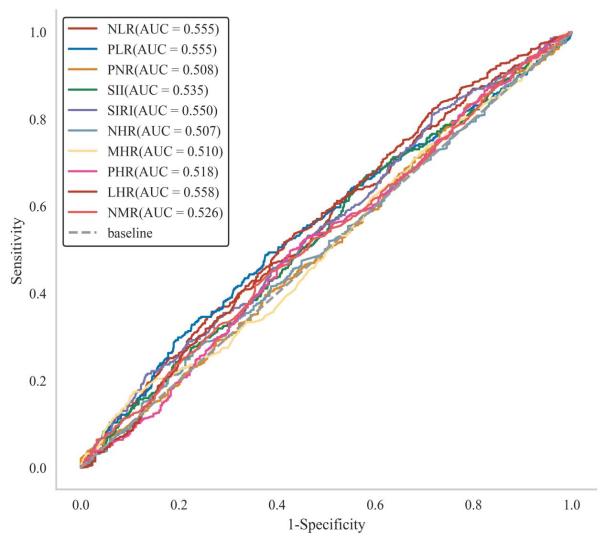 |
| --- | --- |
| 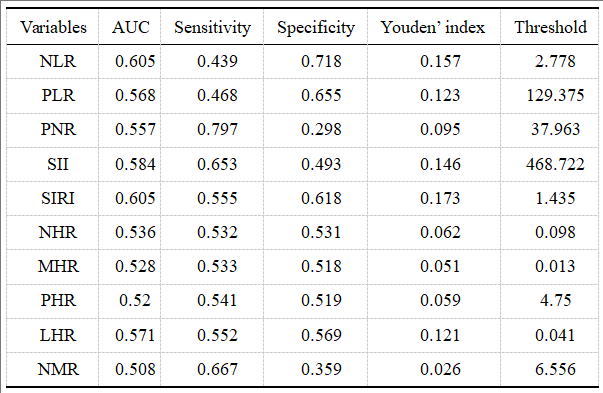 | 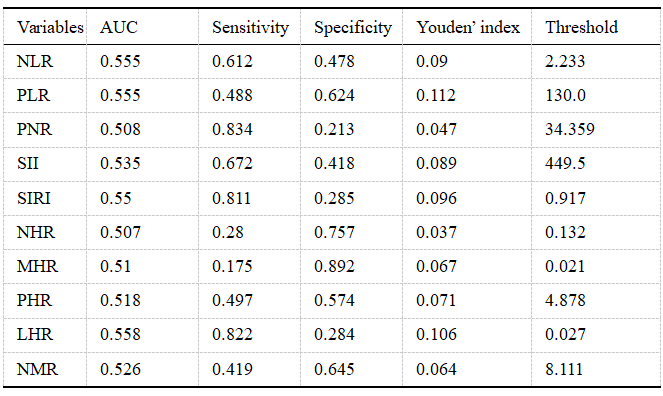 |
| **Figure S3**: Single-Variable ROC Curves of ten inflammatory and nutritional parameters for predicting (A) All-cause mortality and (B) Cardiovascular mortality. | |

| A.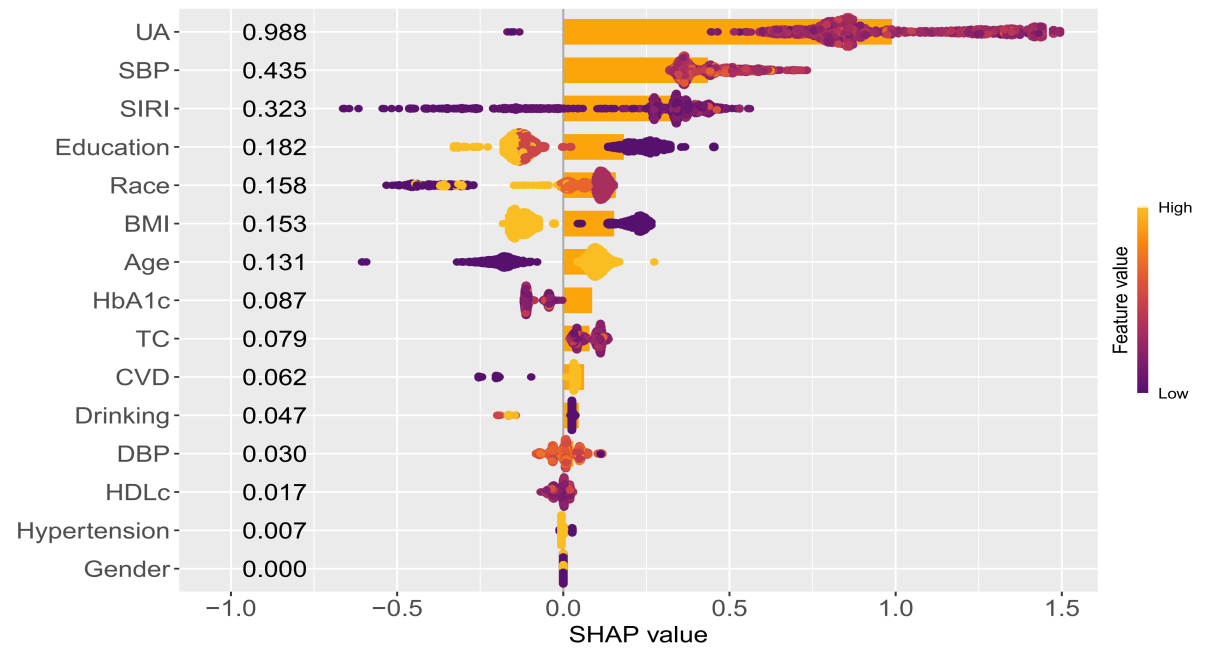 |
| --- |
| B.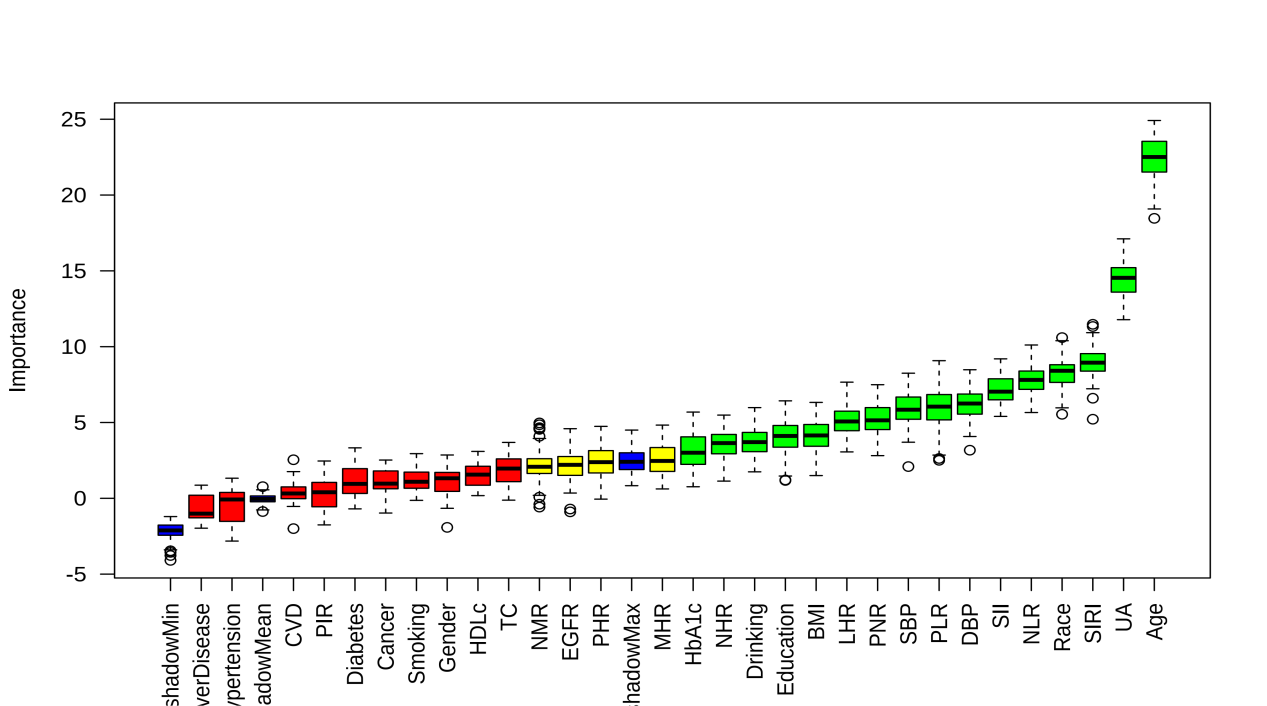 |
| C.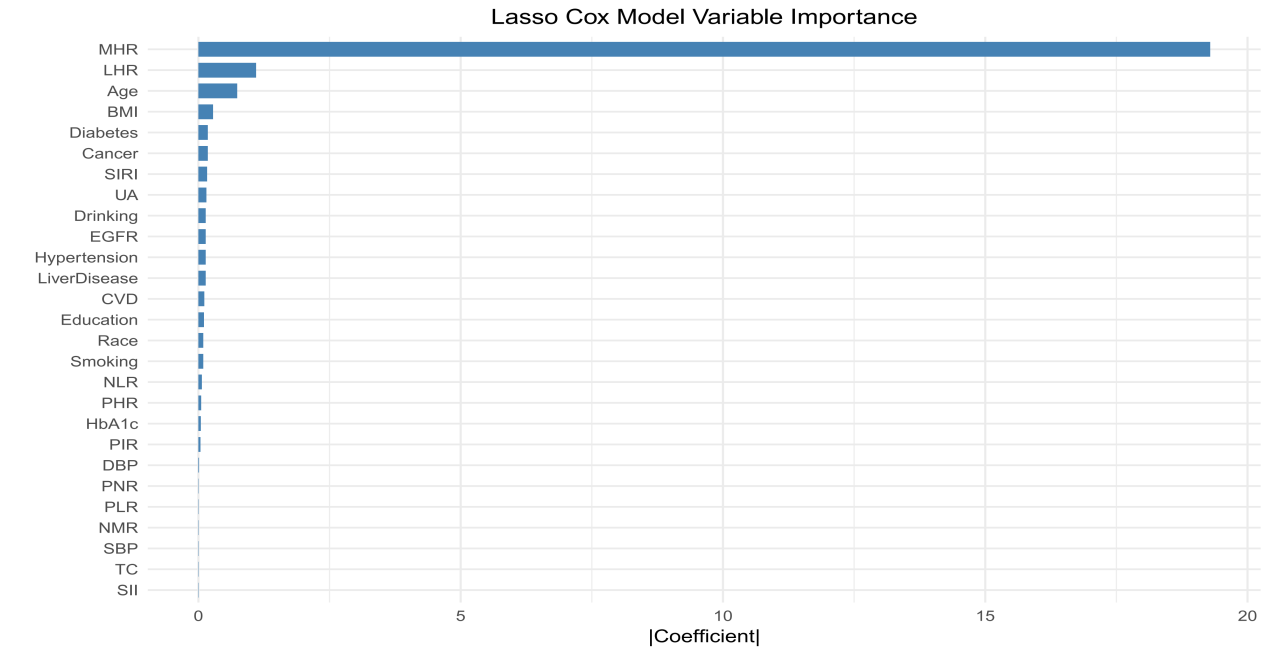 |
| **Figure S4:** (A) SHAP value analysis; (B) Boruta algorithm; (C) Lasso regression on the model incorporating all baseline variables and ten inflammatory and nutritional markers for predicting all-cause mortality. |

| A.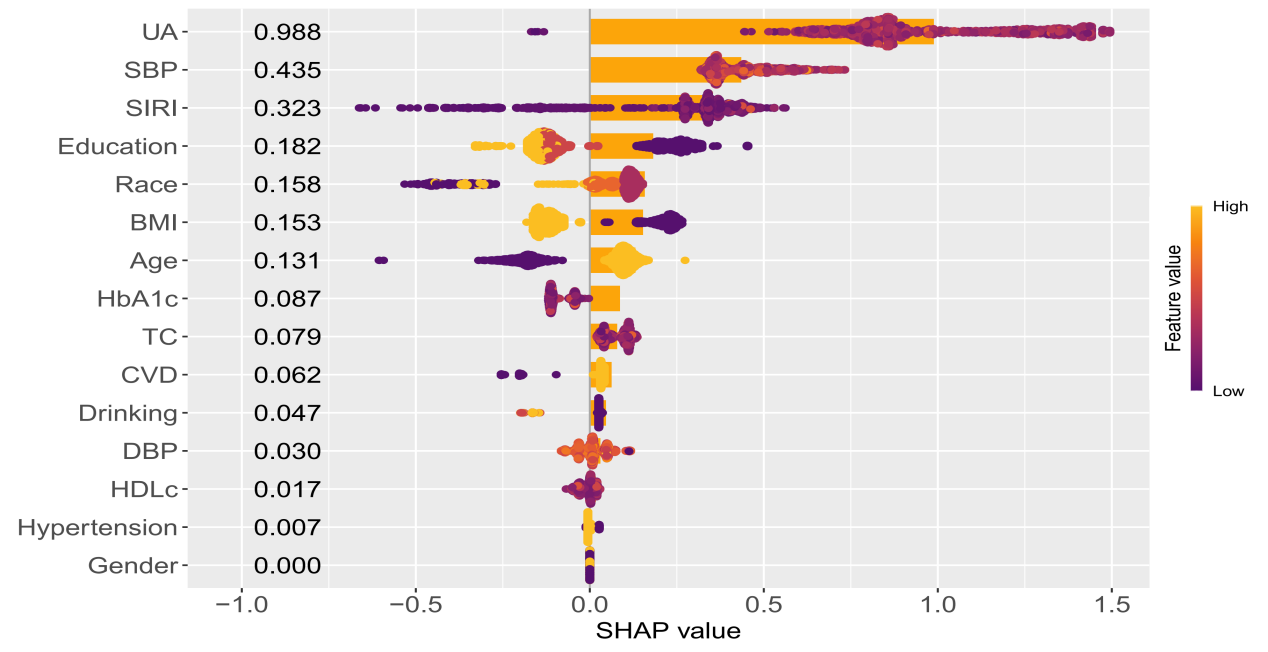 |  |
| --- | --- |
| B.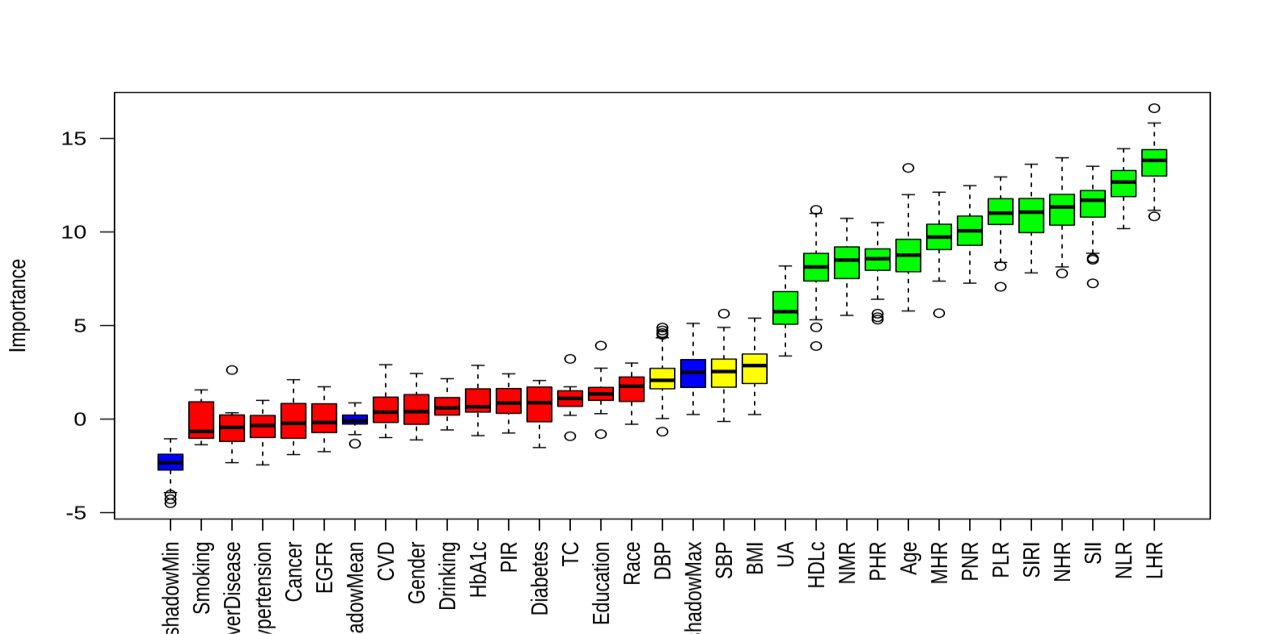 |  |
| C.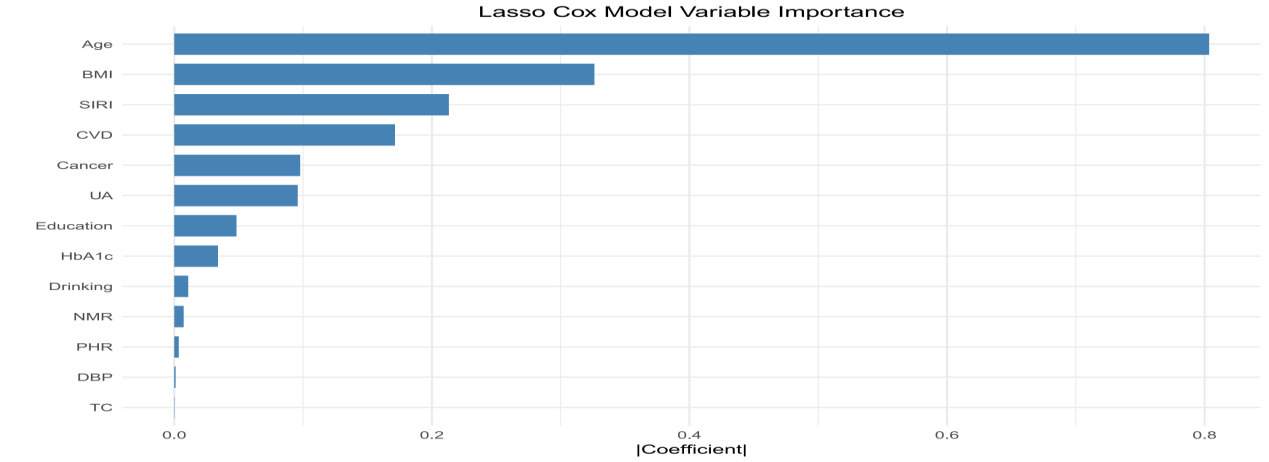 |  |
| **Figure S5:** (A) SHAP value analysis; (B) Boruta algorithm; (C) Lasso regression on the model incorporating all baseline variables and ten inflammatory and nutritional markers for predicting cardiovascular mortality. |  |
| A.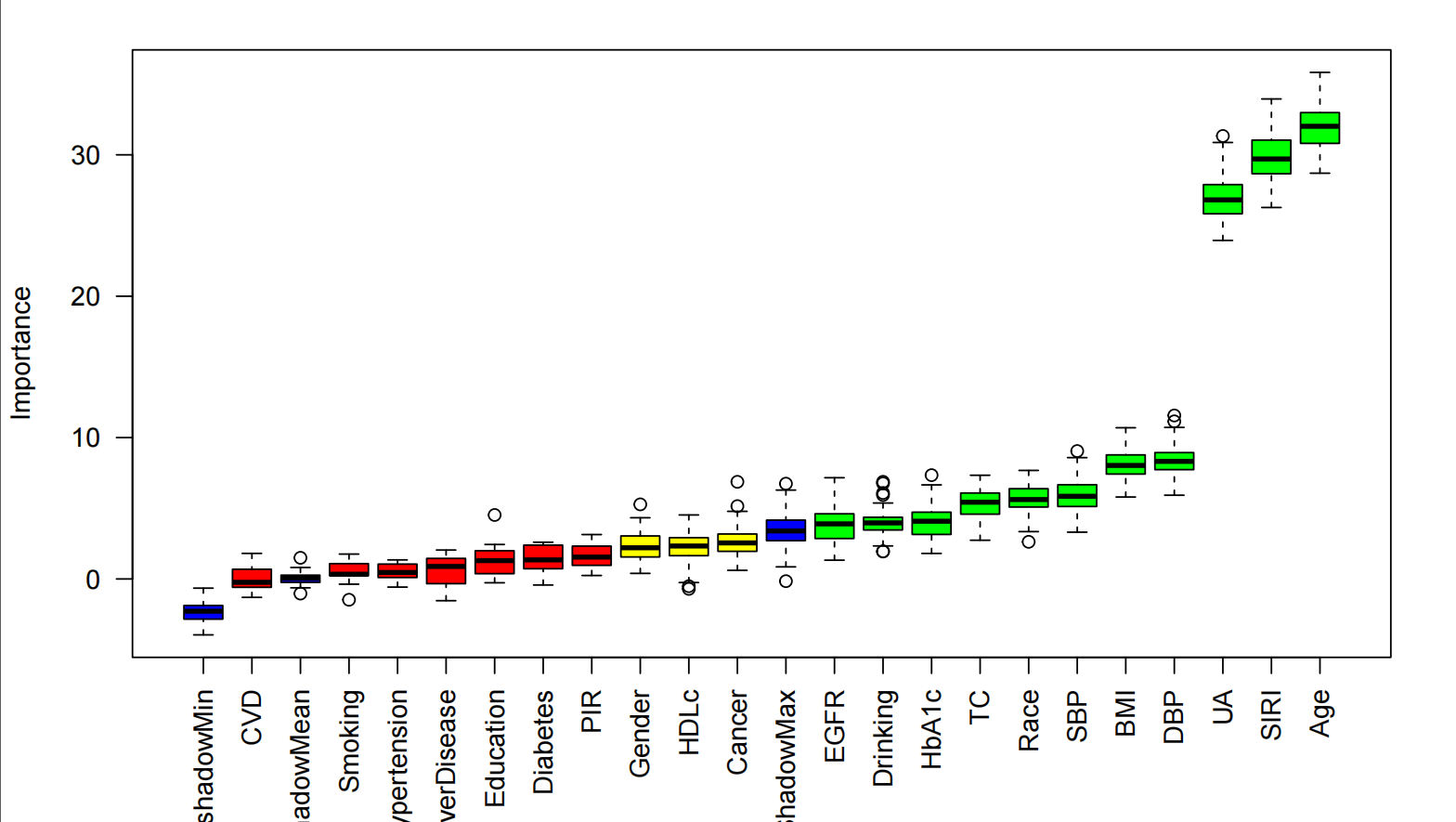 | |
| B.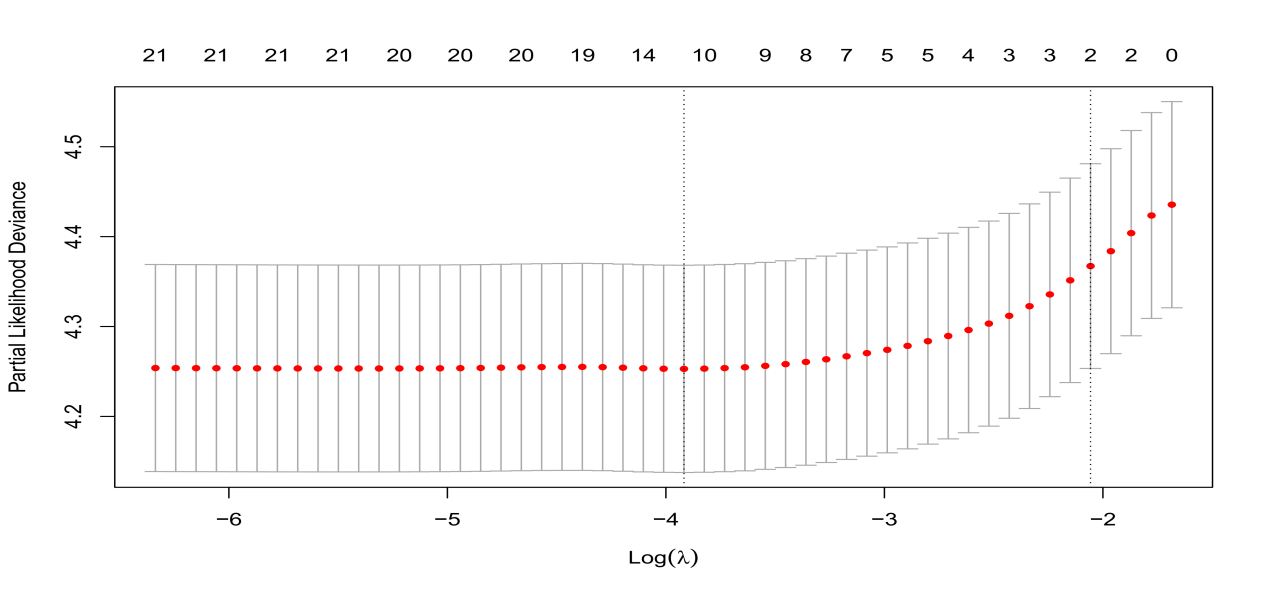 | |
| C.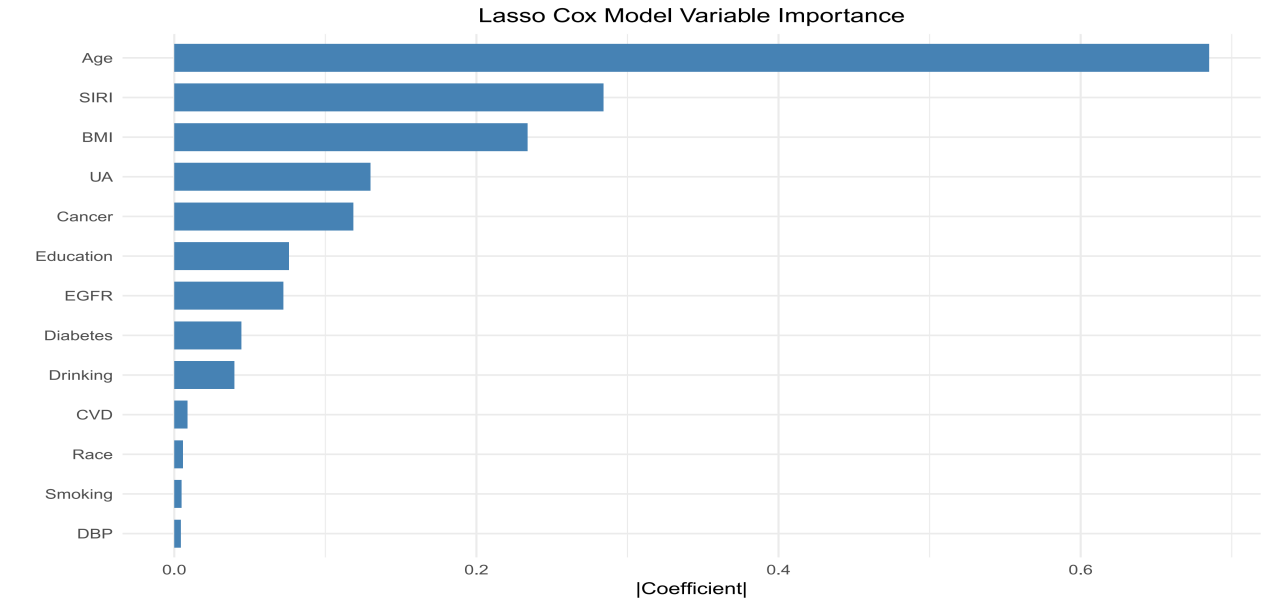 | |
| **Figure S6:** (A) Lasso regression; (B) Cross-validation diagram; (C) Boruta algorithm on the model incorporating SIRI and all baseline variables for predicting all-cause mortality. | |
| A.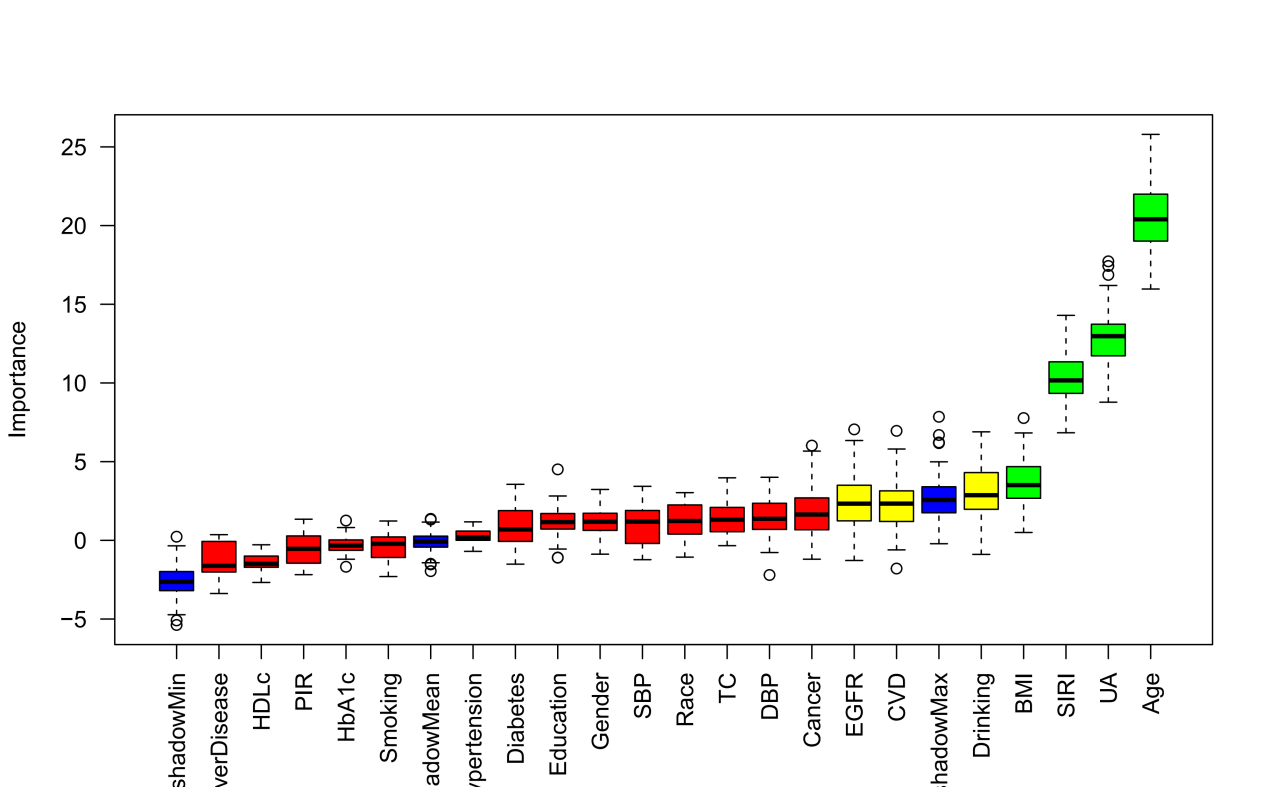 | |
| B.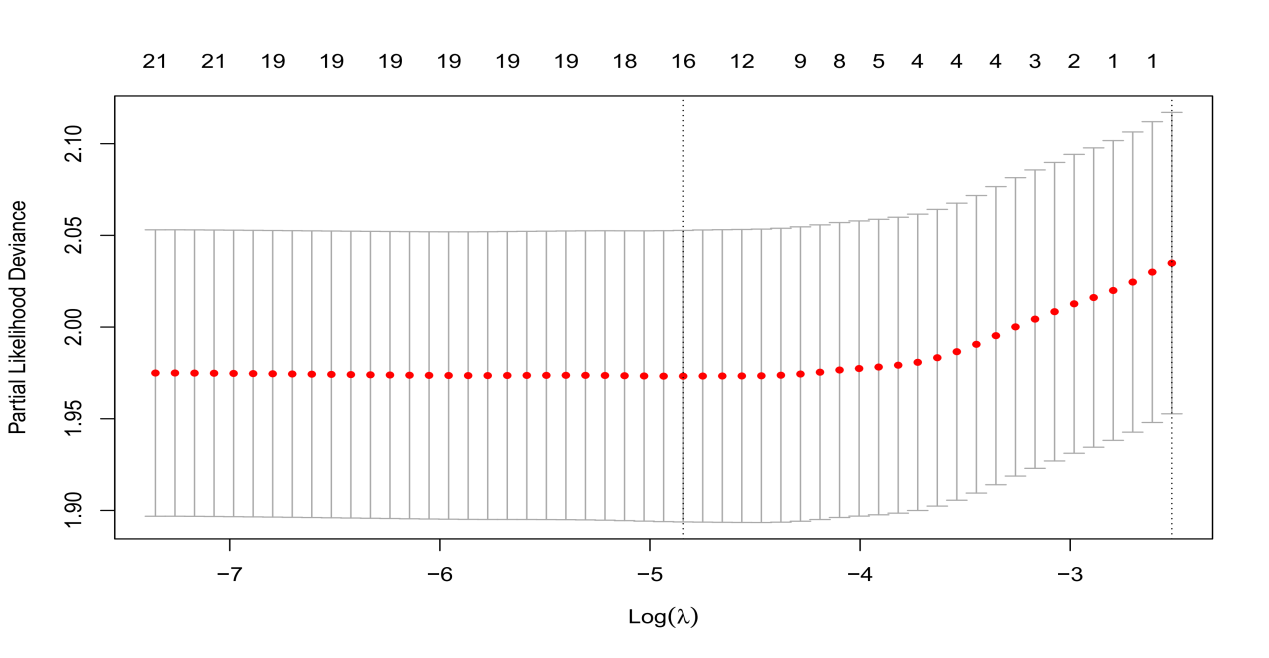 | |
| C.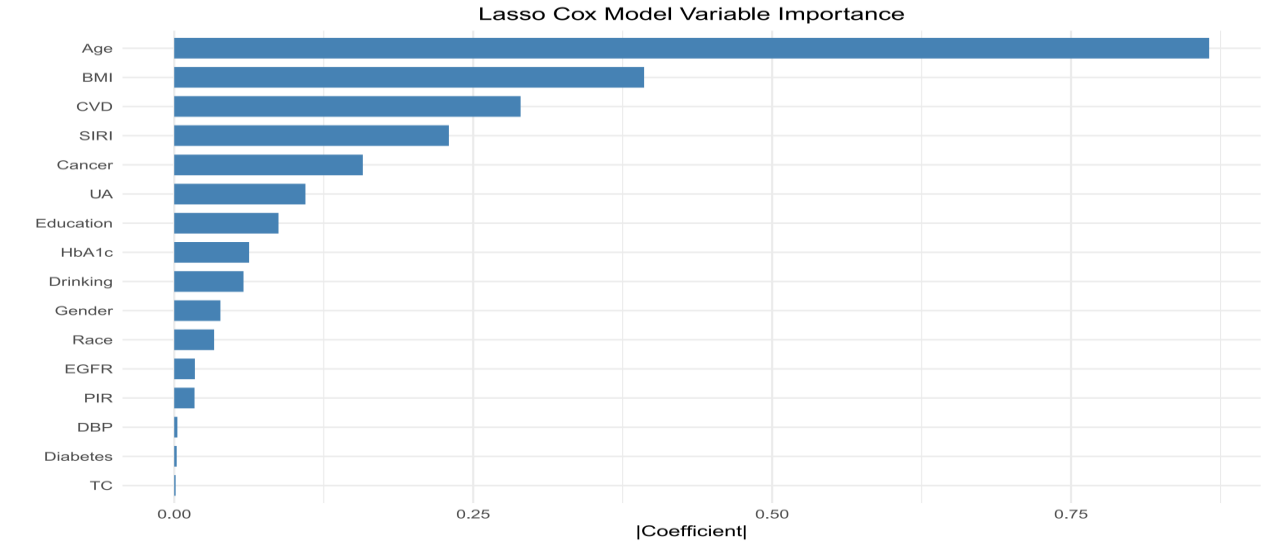 | |
| **Figure S7:** (A) Lasso regression; (B) Cross-validation diagram; (C) Boruta algorithm on the model incorporating SIRI and all baseline variables for predicting cardiovascular mortality. | |

| A.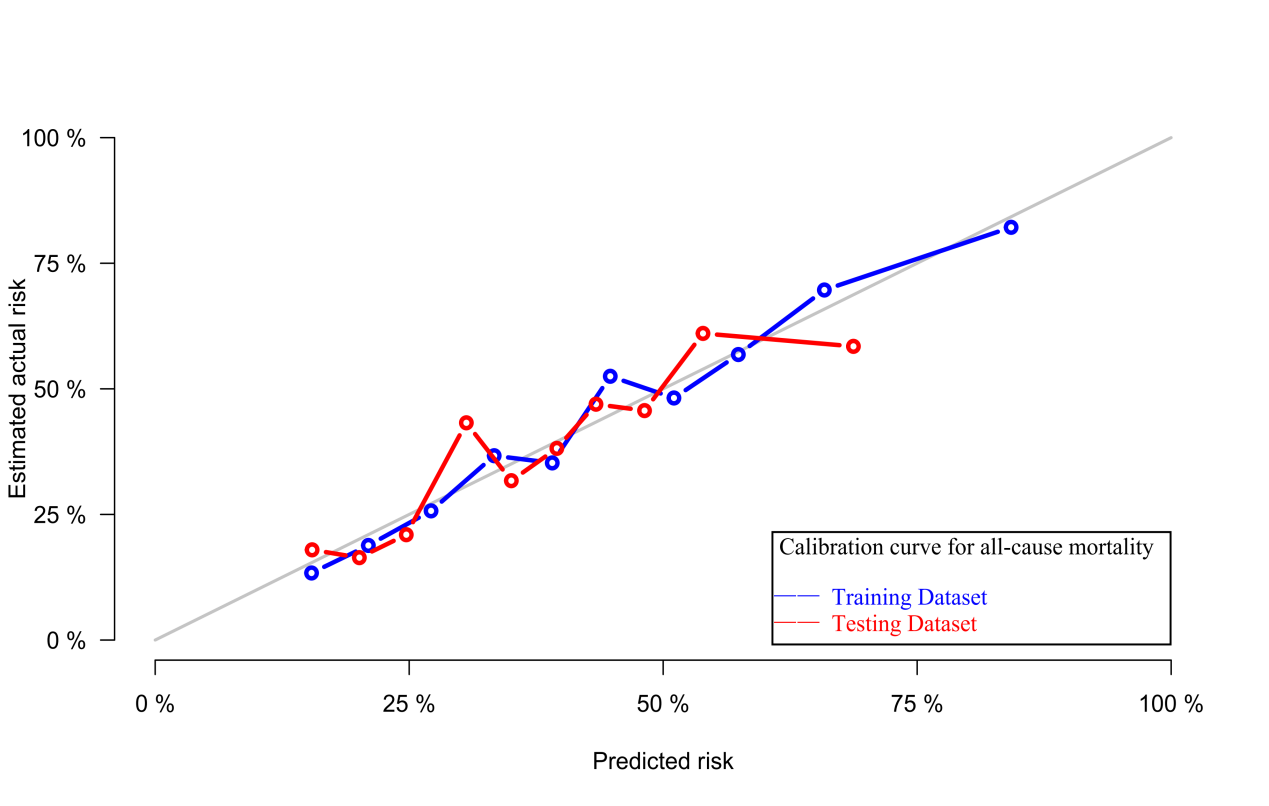 |
| --- |
| B.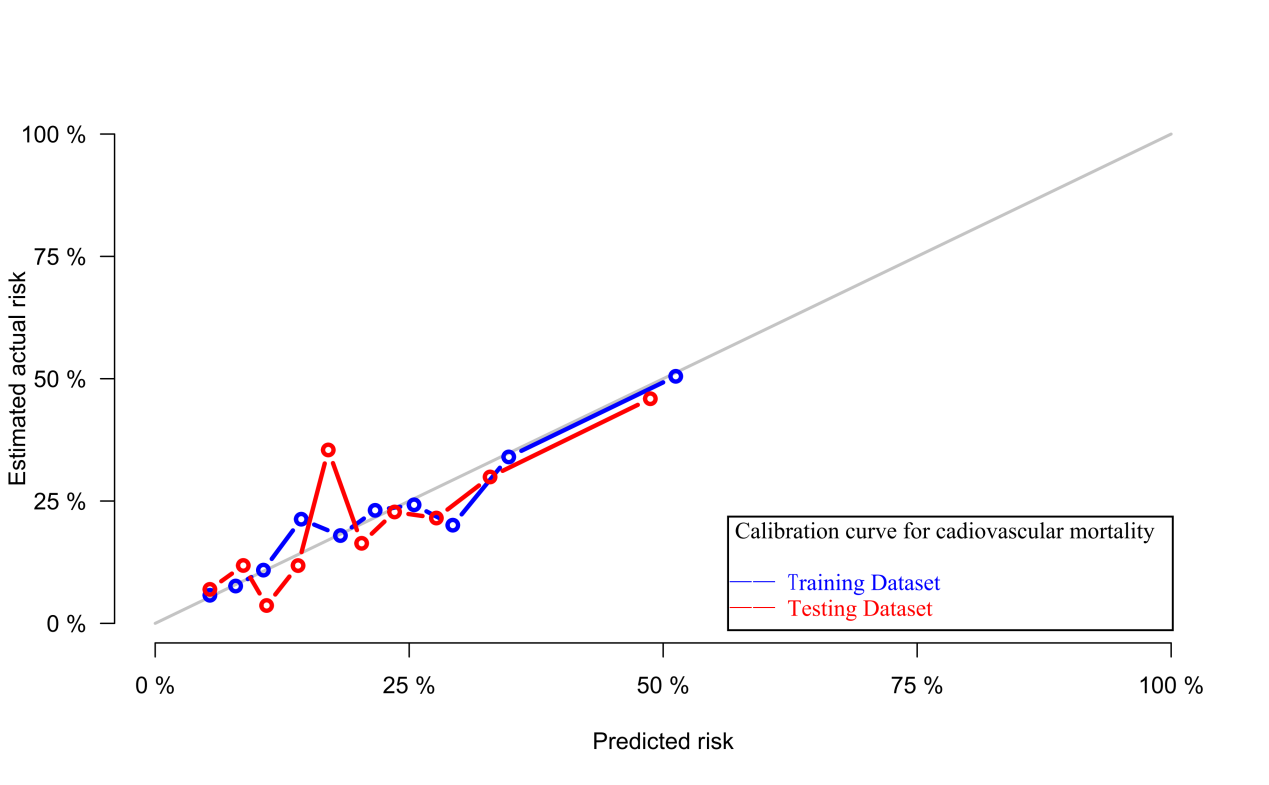 |
| **Figure S8:** (A) Calibration chart of the formed prognostic model in **predicting all-cause mortality at 60 months,120 months, 150 months time points.** (B) Calibration chart of the formed prognostic model in **predicting cardiovascular mortality at 60 months,120 months, 150 months time points.** |

| A.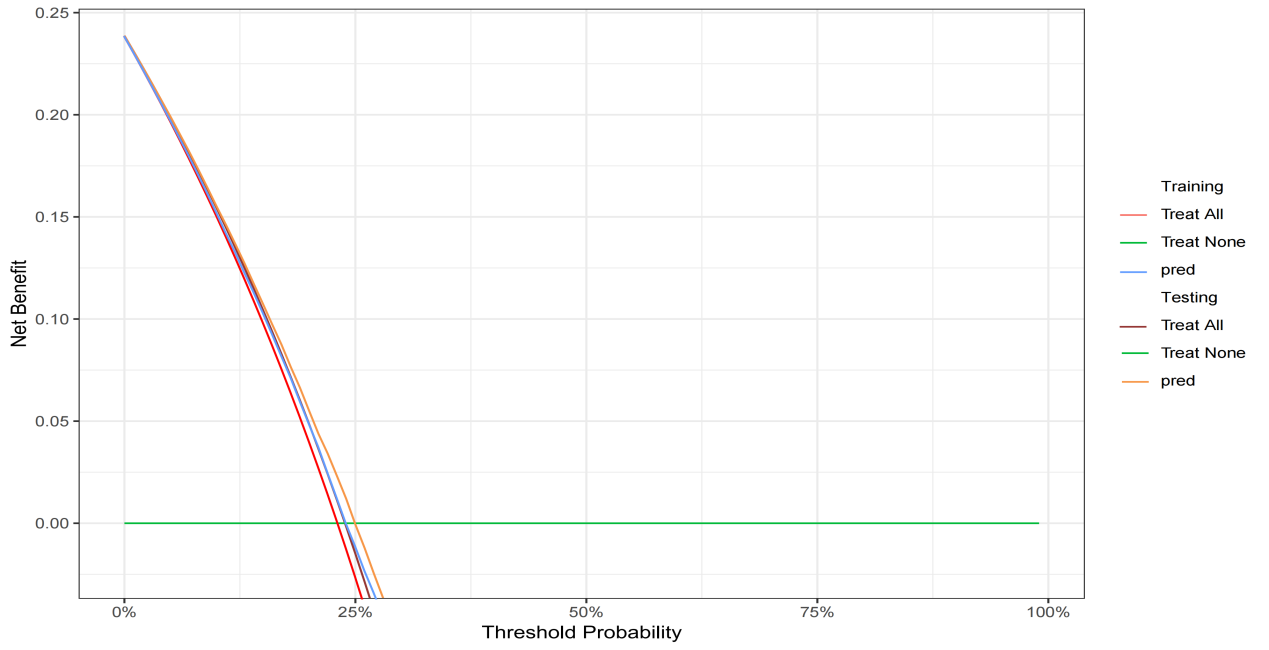 | | |
| --- | --- | --- |
| B.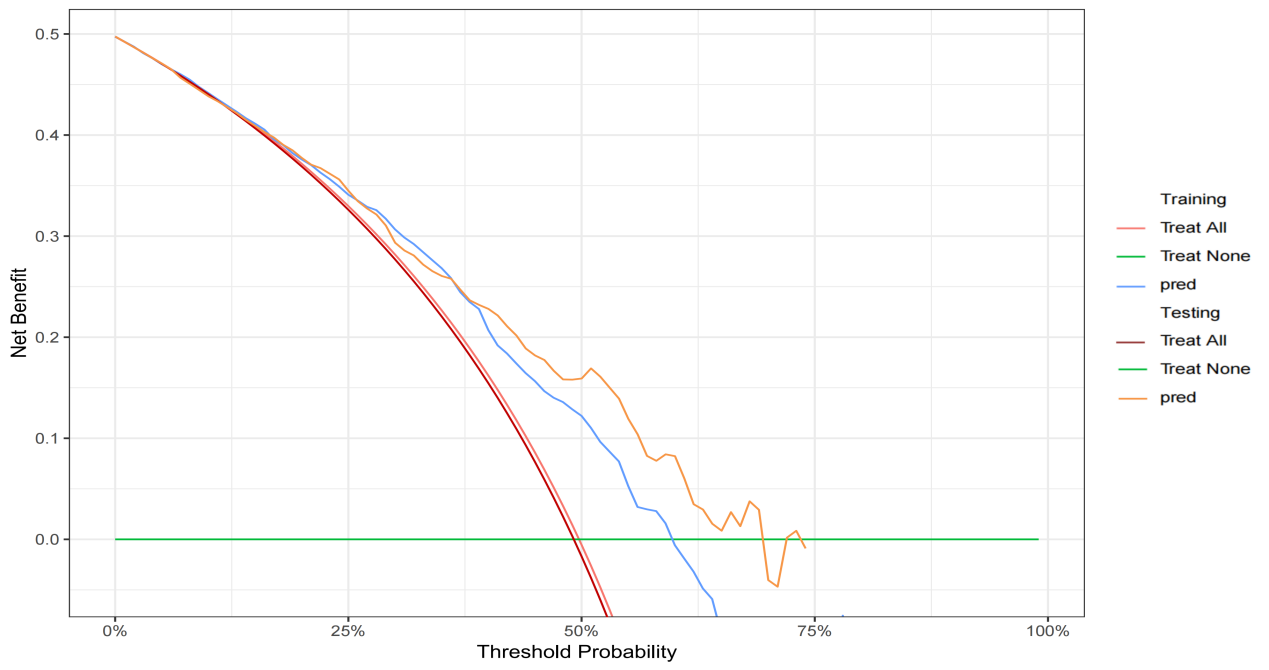 | | |
| C.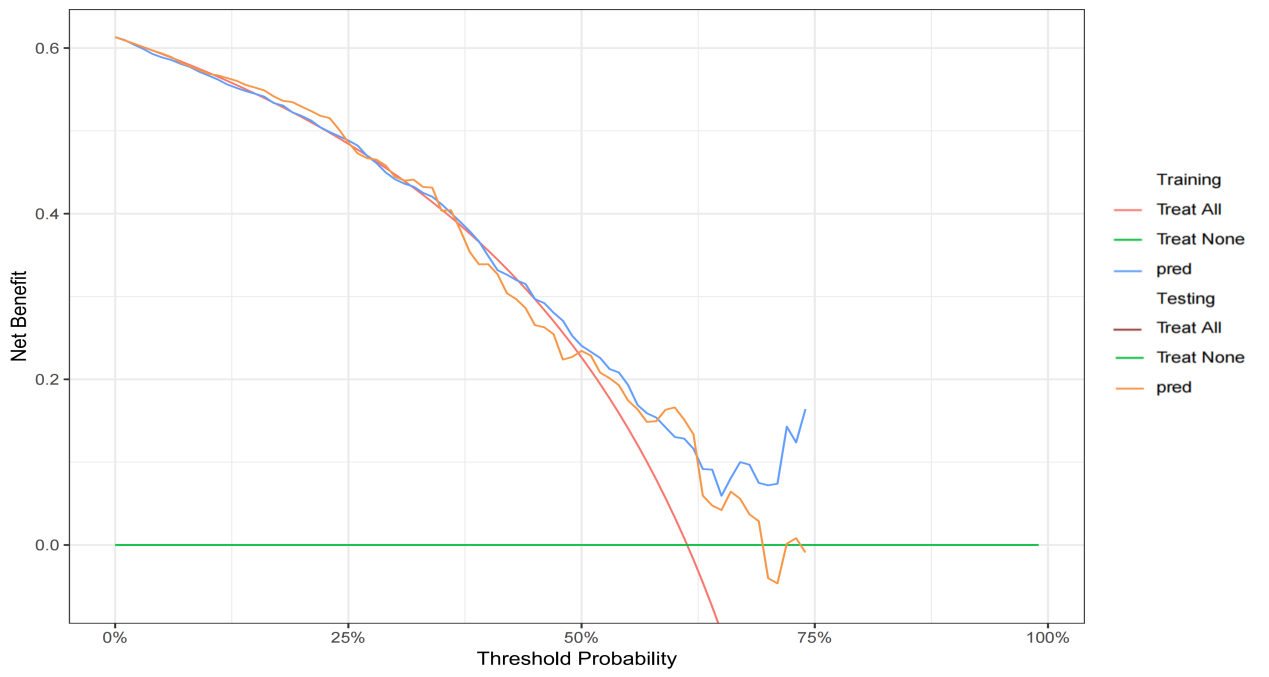 | | |
| **Figure S9:** (A) Decision curve analysis (DCA) of the formed prognostic model in **predicting all-cause mortality at 60 months.** (B) DCA of the formed prognostic model in **predicting all-cause mortality at 120 months. (C)** DCA of the formed prognostic model in **predicting all-cause mortality at 150 months.** | | |
| A.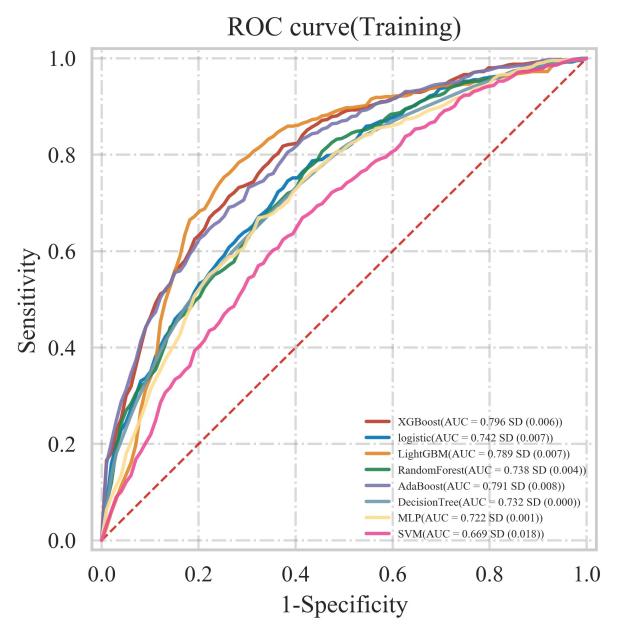 | B.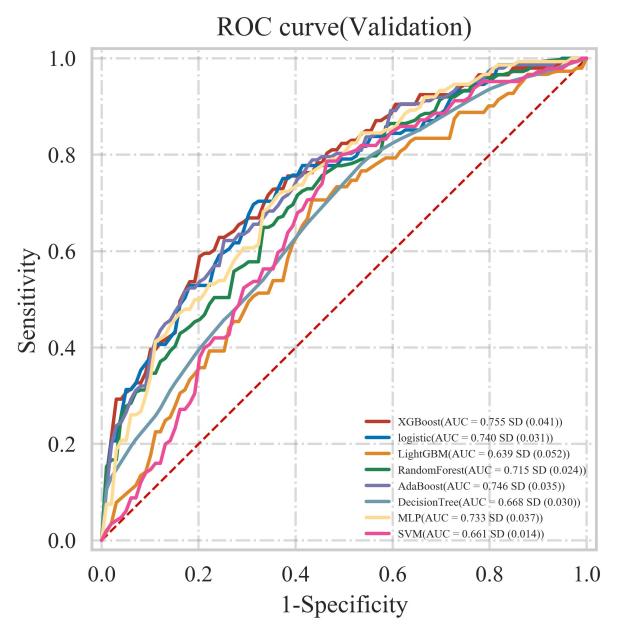 | |
| C.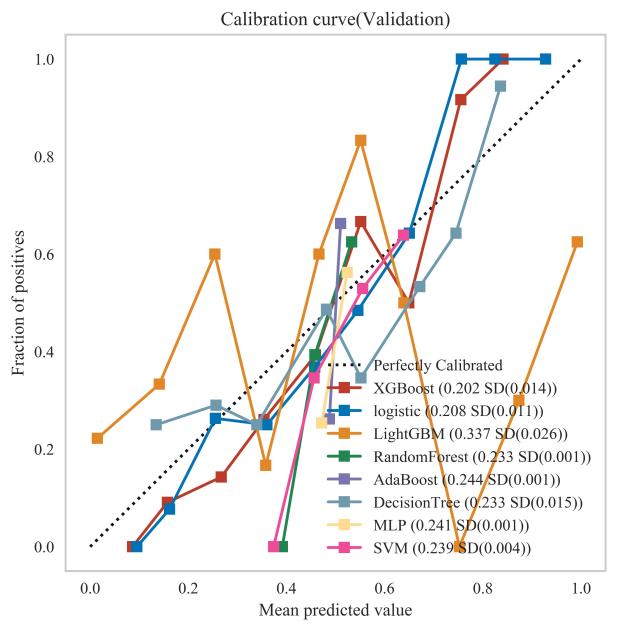 | D.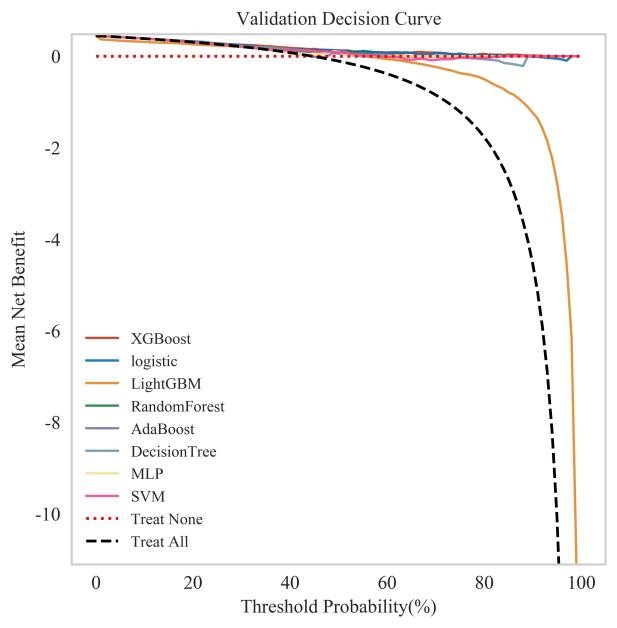 | |
| **Figure S10:** (A) ROC curves in training dataset; (B) ROC curves in validation dataset; (C) Calibration cures; (D) Validation decision curves of the predictive model for all-mortality at 60 months. | | |

| A.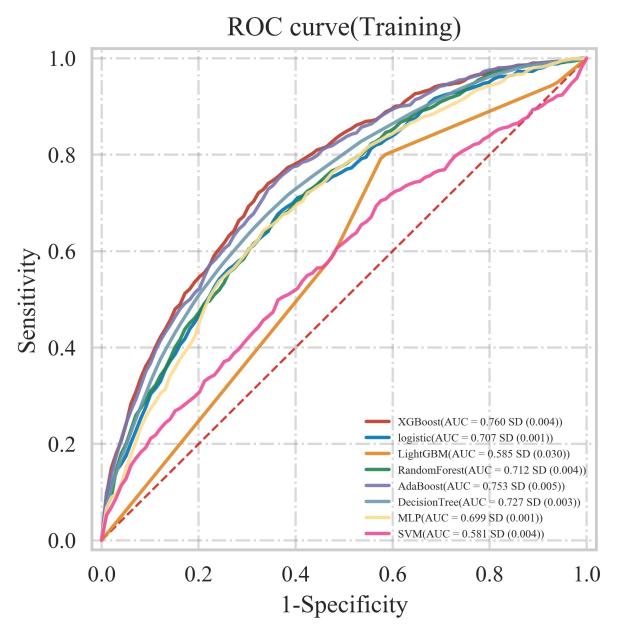 | B.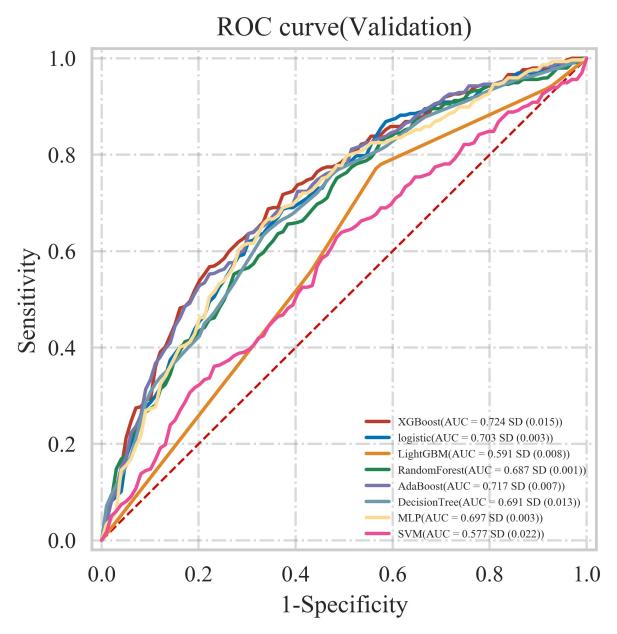 |
| --- | --- |
| C.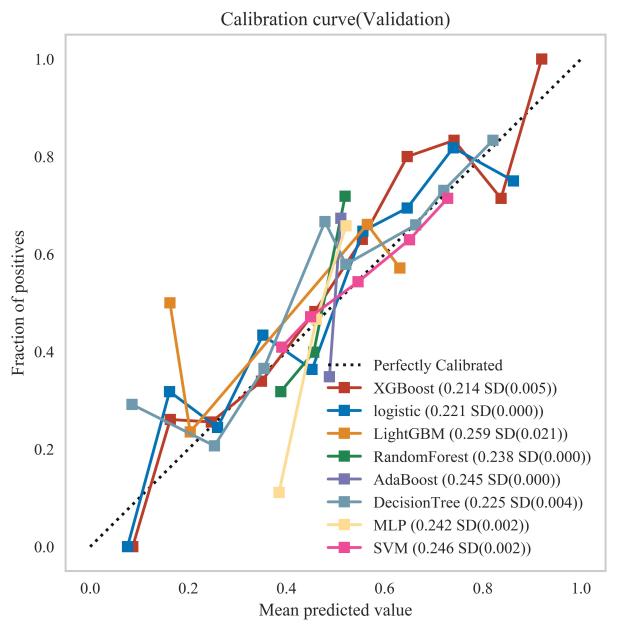 | D.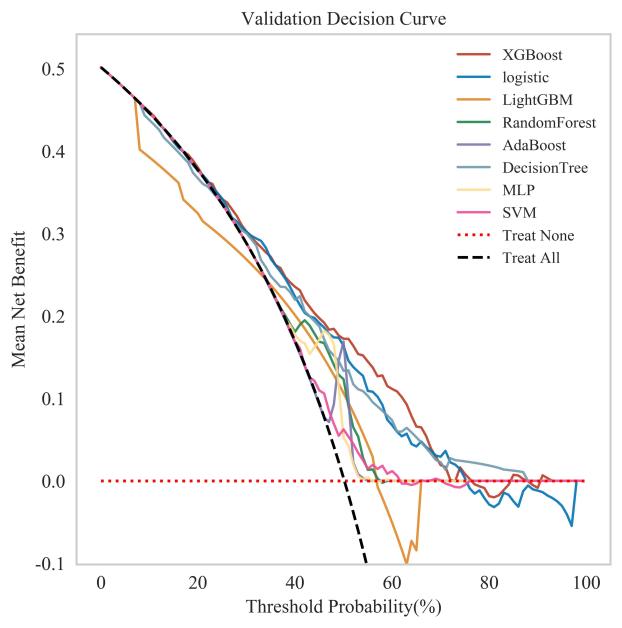 |
| **Figure S11:** (A) ROC curves in training dataset; (B) ROC curves in validation dataset; (C) Calibration cures; (D) Validation decision curves of the predictive model for all-mortality at 120 months. | |

| A.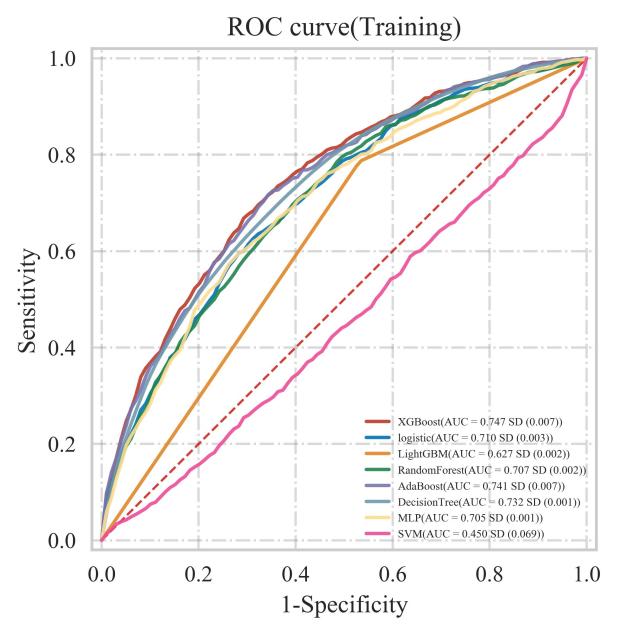 | B.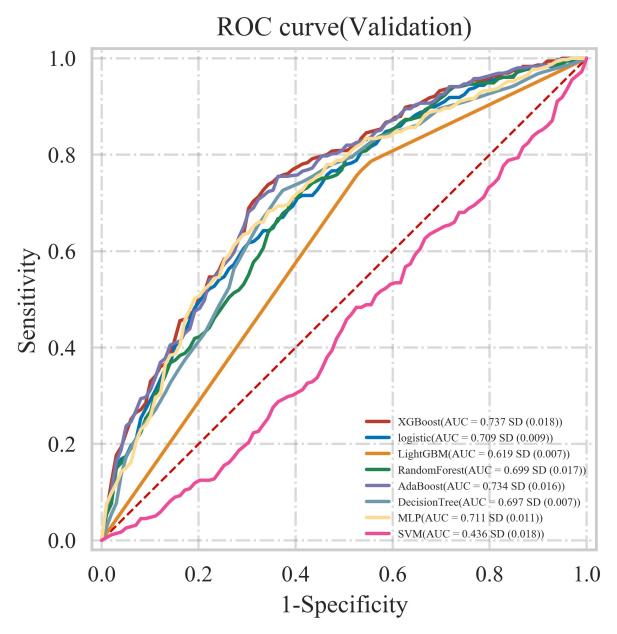 |
| --- | --- |
| C.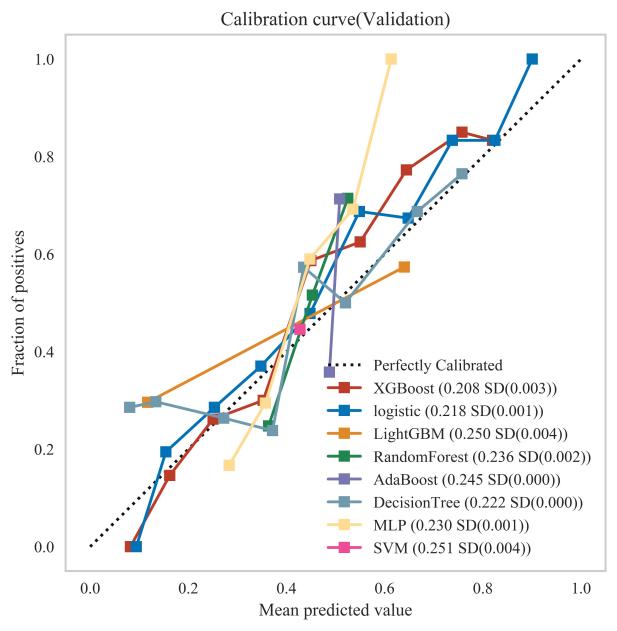 | D.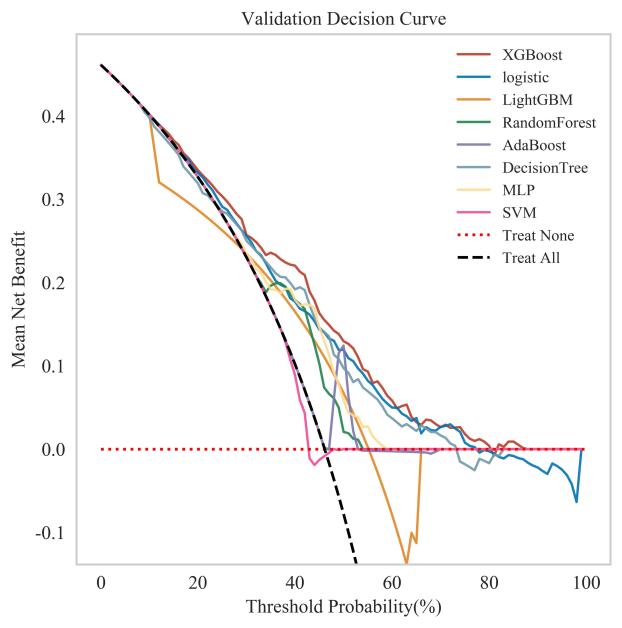 |
| **Figure S12:** (A) ROC curves in training dataset; (B) ROC curves in validation dataset; (C) Calibration cures; (D) Validation decision curves of the predictive model for all-mortality at 150 months. | |

| A.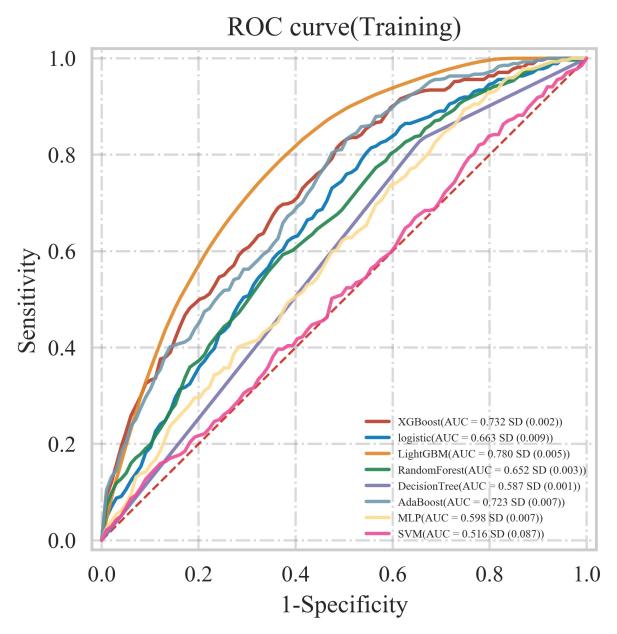 | B.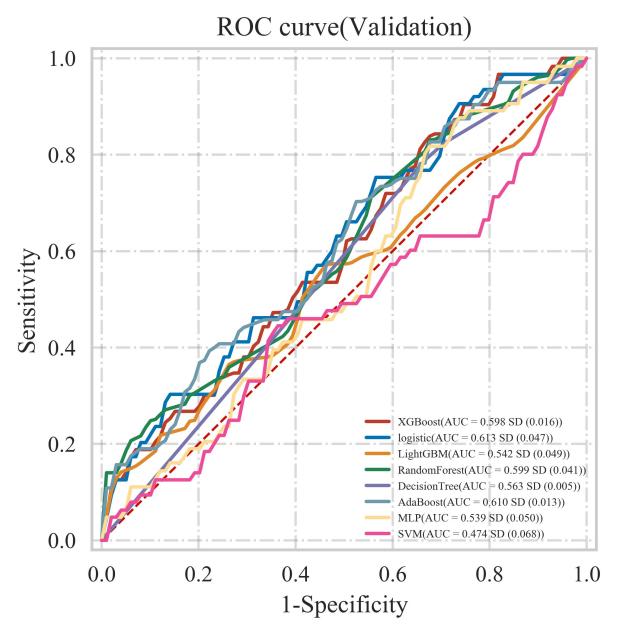 |
| --- | --- |
| C.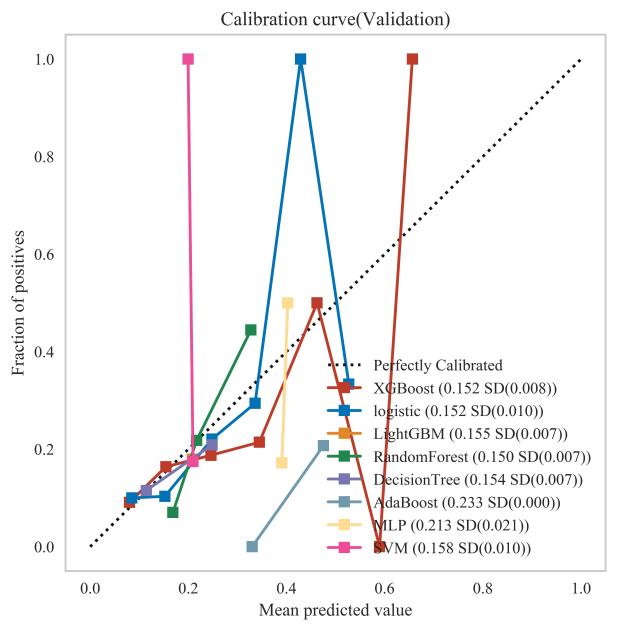 | D.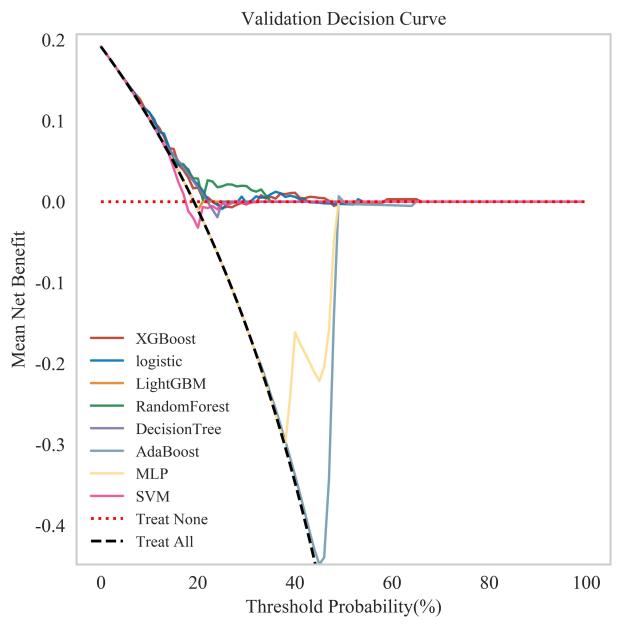 |
| **Figure S13:** (A) ROC curves in training dataset; (B) ROC curves in validation dataset; (C) Calibration cures; (D) Validation decision curves of the predictive model for cardiovascular mortality at 60 months. | |

| A.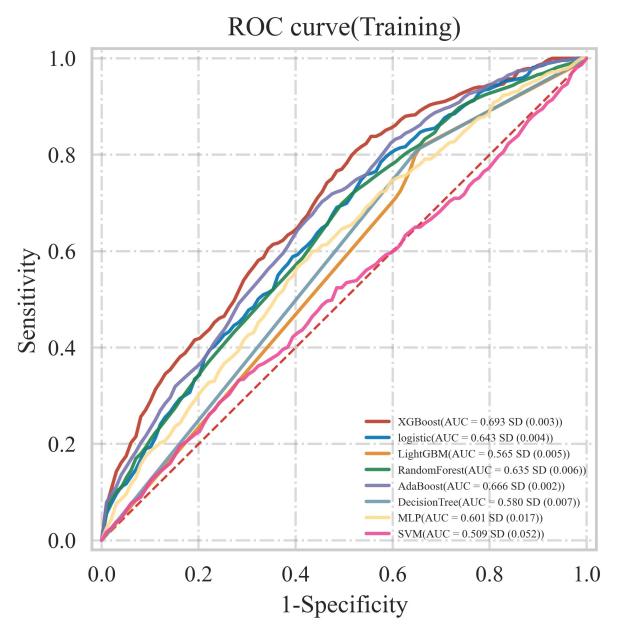 | B.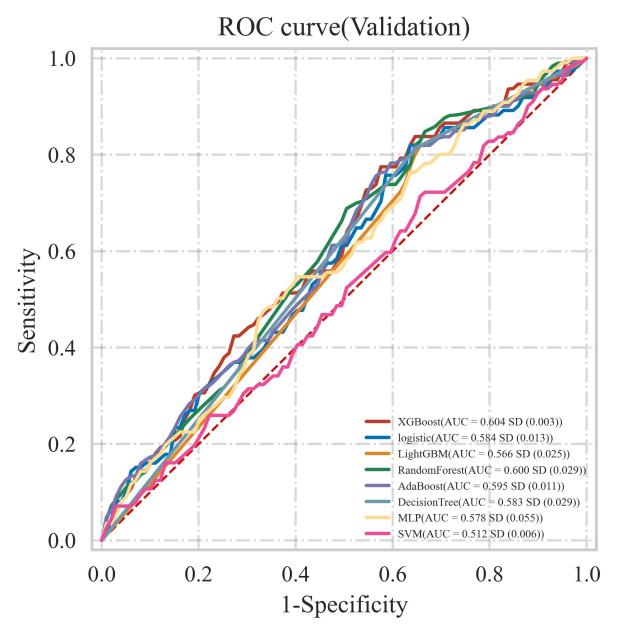 |
| --- | --- |
| C.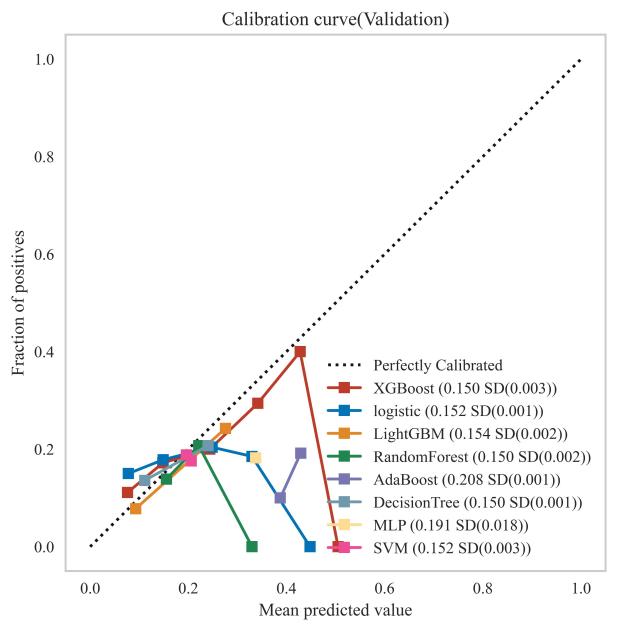 | D.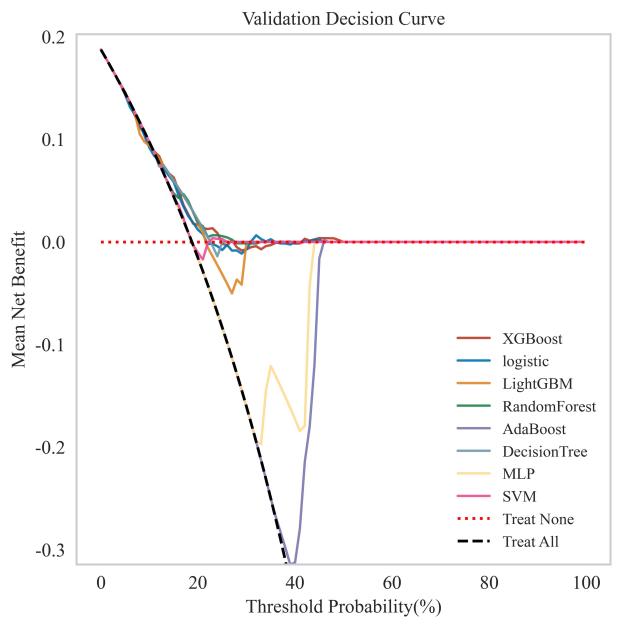 |
| **Figure S14:** (A) ROC curves in training dataset; (B) ROC curves in validation dataset; (C) Calibration cures; (D) Validation decision curves of the predictive model for cardiovascular mortality at 120 months. | |

| A.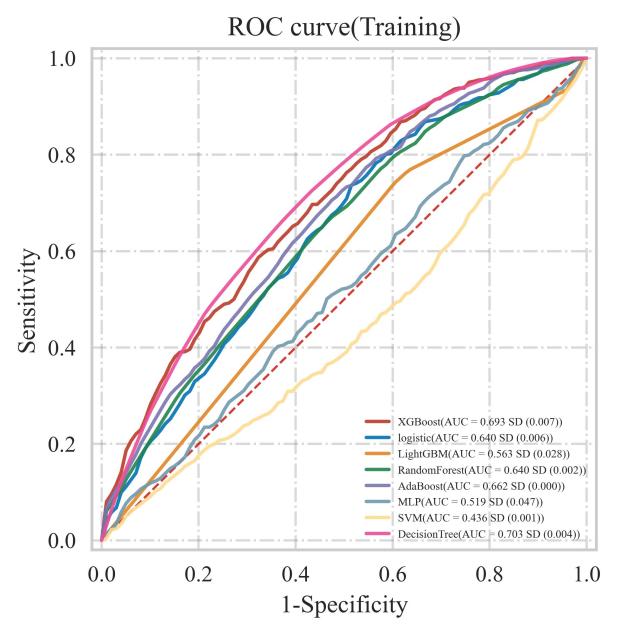 | B.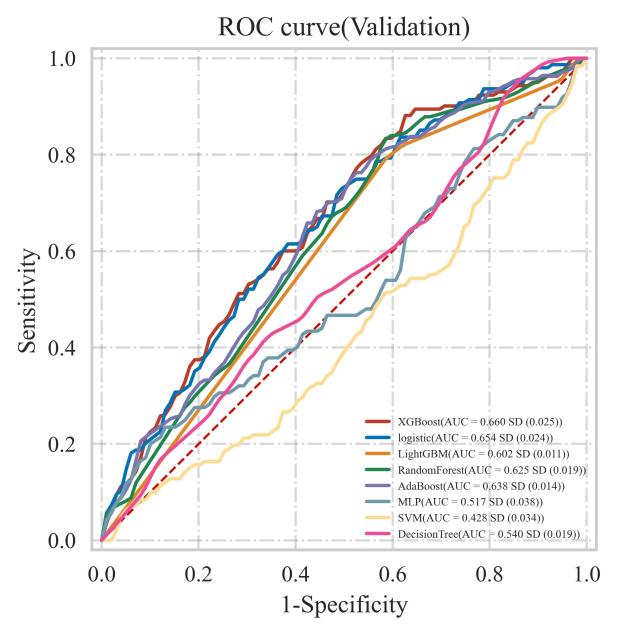 |
| --- | --- |
| C.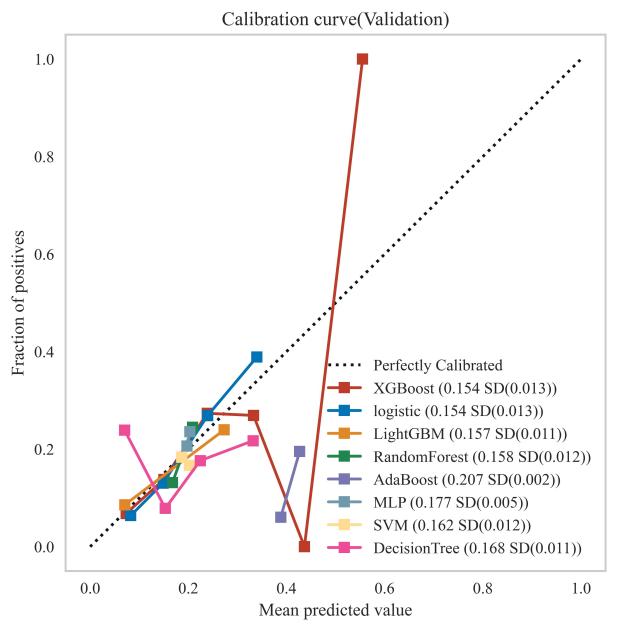 | D.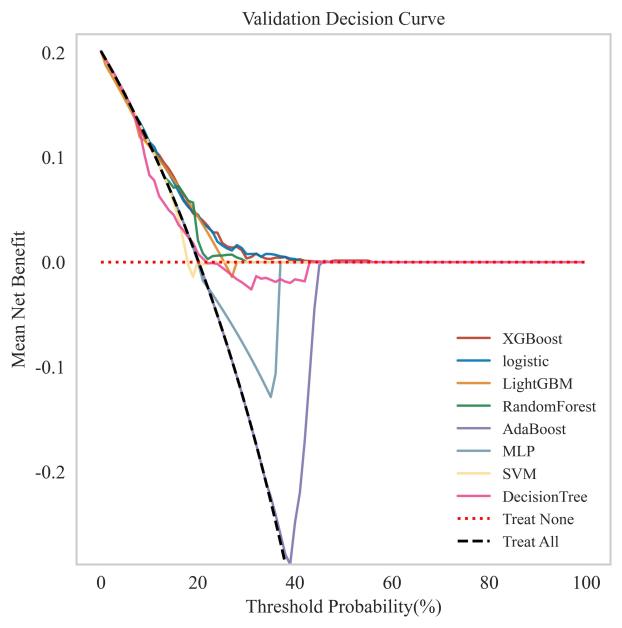 |
| **Figure S15:** (A) ROC curves in training dataset; (B) ROC curves in validation dataset; (C) Calibration cures; (D) Validation decision curves of the predictive model for cardiovascular mortality at 150 months. | |

| A.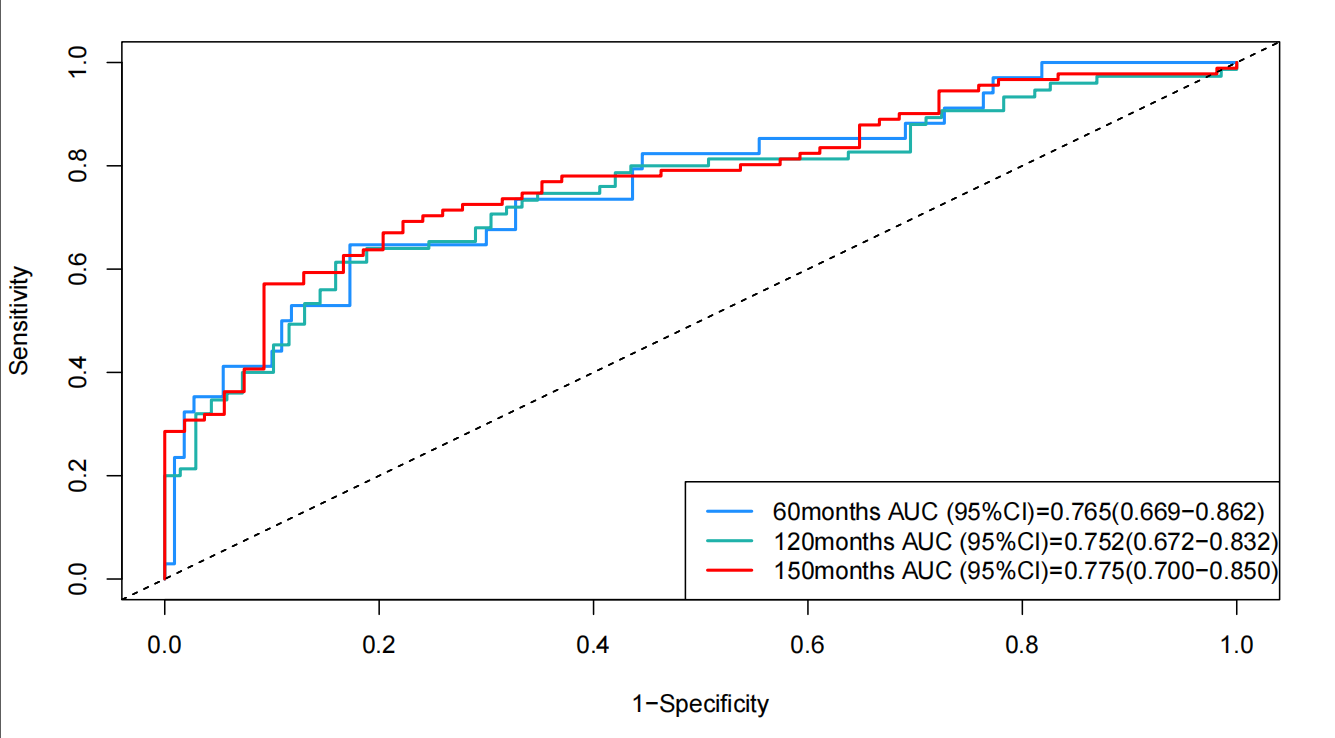 |
| --- |
| B.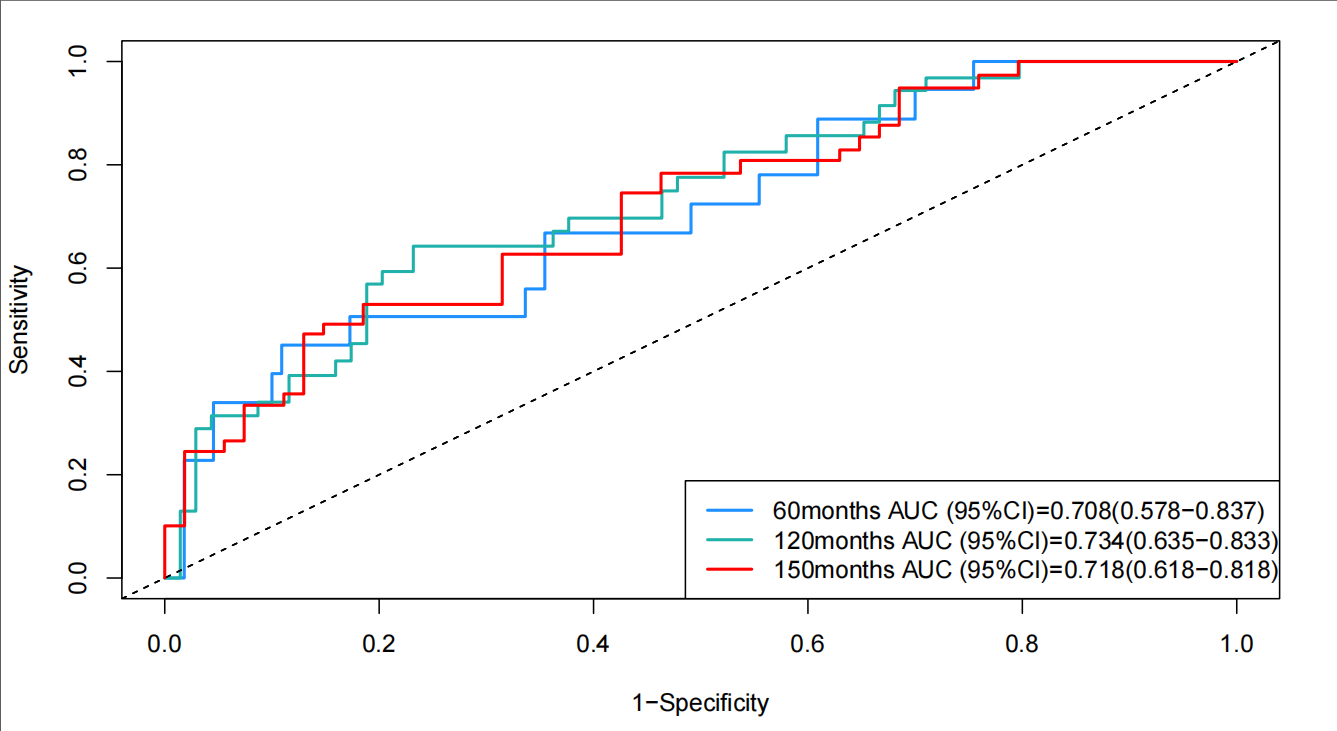 |
| C.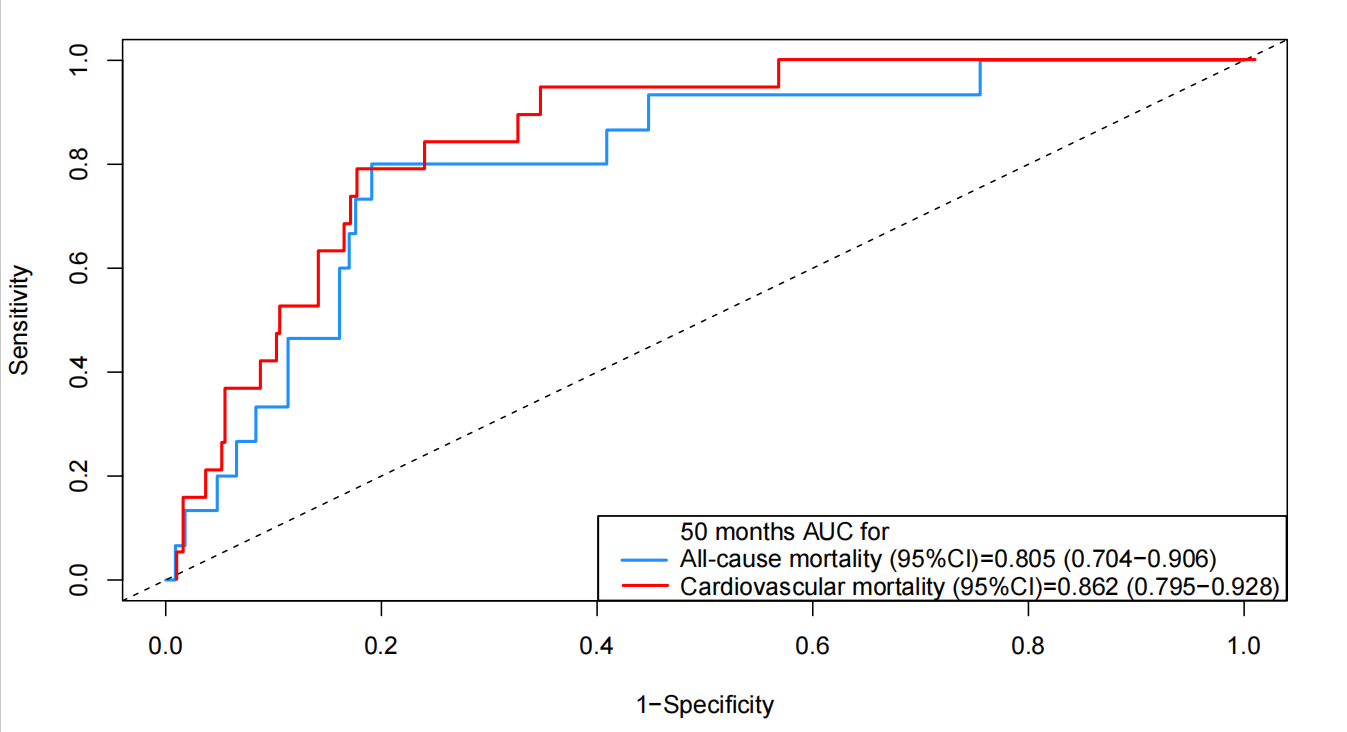 |
| **Figure S16:** (A) ROC curves of the formed prognostic model in **predicting NHANES 1999-2000 population’s all-cause mortality at 60 months,120 months, 150 months time points.** (B) ROC curves of the formed prognostic model in **predicting NHANES 1999-2000 population’s cardiovascular mortality at 60 months,120 months, 150 months time points. (C)** ROC curves of the formed prognostic model in **predicting Gaoyou cohort’s all-cause and cardiovascular mortality at 50 months.** |
